# Rotaxane Co^II^ Complexes as Field‐Induced Single‐Ion Magnets

**DOI:** 10.1002/anie.202103596

**Published:** 2021-06-14

**Authors:** Martina Cirulli, Enrico Salvadori, Zhi‐Hui Zhang, Michael Dommett, Floriana Tuna, Heiko Bamberger, James E. M. Lewis, Amanpreet Kaur, Graham J. Tizzard, Joris van Slageren, Rachel Crespo‐Otero, Stephen M. Goldup, Maxie M. Roessler

**Affiliations:** ^1^ School of Biological and Chemical Sciences Queen Mary University of London Mile End Road London E1 4NS UK; ^2^ Department of Chemistry University of Torino Via Giuria 7 10125 Torino Italy; ^3^ Chemistry University of Southampton Highfield SO 17 1BJ UK; ^4^ Department of Chemistry and Photon Science Institute University of Manchester Oxford Road Manchester M13 0PL UK; ^5^ Institut für Physikalische Chemie Universität Stuttgart Pfaffenwaldring 55 70569 Stuttgart Germany; ^6^ Department of Chemistry Imperial College London Molecular Sciences Research Hub Wood Lane London W12 0BZ UK; ^7^ EPSRC National Crystallographic Service University of Southampton Highfield Southampton SO17 1BJ UK

**Keywords:** electron paramagnetic resonance, magnetism, rotaxanes, single ion magnets, zero-field splitting

## Abstract

Mechanically chelating ligands have untapped potential for the engineering of metal ion properties. Here we demonstrate this principle in the context of Co^II^‐based single‐ion magnets. Using multi‐frequency EPR, susceptibility and magnetization measurements we found that these complexes show some of the highest zero field splittings reported for five‐coordinate Co^II^ complexes to date. The predictable coordination behaviour of the interlocked ligands allowed the magnetic properties of their Co^II^ complexes to be evaluated computationally a priori and our combined experimental and theoretical approach enabled us to rationalize the observed trends. The predictable magnetic behaviour of the rotaxane Co^II^ complexes demonstrates that interlocked ligands offer a new strategy to design metal complexes with interesting functionality.

## Graphical abstract

Mechanically interlocked ligands enable predictable metal ion coordination, offering a new strategy to engineer their properties. A series of field‐induced Co^II^ single‐ion magnets illustrates this principle. The predictable coordination behaviour of the rotaxane enables remarkably accurate calculation of their zero‐field splittings, which are in agreement with experimental observations, and can be rationalized in terms of metal–ligand bonding.

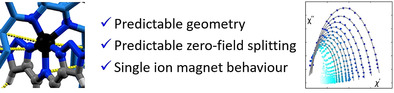

## Introduction

Interlocked molecules[^1^, ^2^, ^3^, ^4^, ^5^] contain cavities within which donor atoms can be positioned to bind metal ions.[^6^, ^7^, ^8^, ^9^, ^10^, ^11^, ^12^, ^13^] Indeed, some of the first observations of the properties of the mechanical bond, including its ability to kinetically stabilize metal complexes,[^6^, ^14^, ^15^, ^16^, ^17^, ^18^] were made by Sauvage and co‐workers over 30 years ago.[^19^, ^20^, ^21^] More recently,^[22]^ we demonstrated that rotaxane‐based ligands can be used to produce complexes the non‐interlocked equivalent of which are inaccessible, including examples reminiscent of the distorted “entatic states” of metalloproteins,[^23^, ^24^] suggesting that interlocked ligands could allow engineering of the properties of metal ions. However, to date, mechanically chelated metal ions with desirable catalytic behaviour,[^25^, ^26^] luminescent properties,[^17^, ^27^] or unusual reactivity[^14^, ^16^, ^18^, ^19^, ^20^, ^21^] have only been reported based on coordination environments that can be accessed with and without the mechanical bond. Thus, the ability to engineer the fundamental properties of metal ions by controlling their coordination environment using the mechanical bond has not been demonstrated in prototypical functional systems.

Since their discovery in 1991,^[28]^ single‐molecule magnets (SMMs) have received significant attention due to their potential applications in spintronics, data storage and quantum computing.^[29]^ The slow relaxation of the magnetization that defines SMMs is typically dictated by an energy barrier (*U*) which arises from the magnetic anisotropy, characterized by the zero‐field splitting term (*D*), of a non‐zero spin ground state. Tailoring the size and sign of *D* therefore represents a promising strategy to design molecules with a large *U*.^[30]^ Single‐ion magnets (SIMs) containing only one metal center are of particular interest due to the possibility of predicting anisotropy based on ligand field theory.[^31^, ^32^] However, most transition‐metal SIMs are discovered serendipitously, not least because *D* relies on subtle geometric effects and accurately predicting the geometry or even stoichiometry of heteroleptic complexes formed from a mixture of ligands remains challenging.^[33]^

Here, for the first time, we show that mechanically chelating ligands represent an untapped platform for the design of SIMs. By focusing on rotaxane‐based Co^II^ complexes, we apply computational approaches for the accurate correlation of magnetic anisotropy[^34^, ^35^] and the electronic structure of Co^II^ ions[^31^, ^32^, ^36^] to demonstrate that the geometry of the complexes formed can be predicted with sufficient precision to identify interesting magnetic properties a priori.

## Results and Discussion

**Figure 1:**
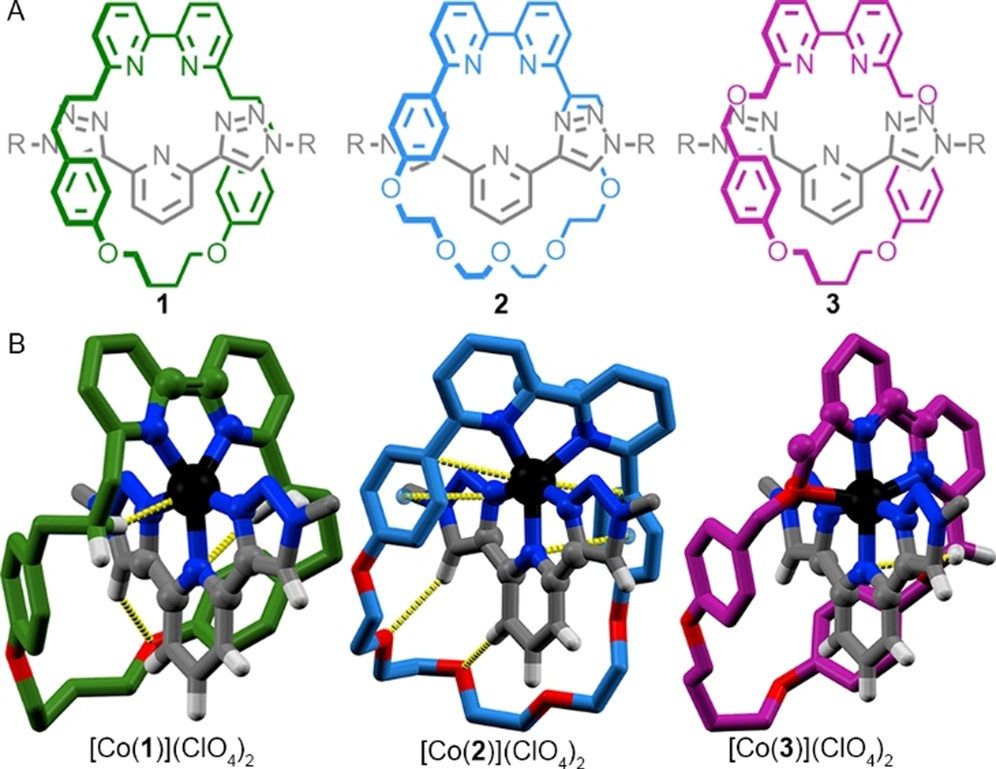
A) Ligands **1**–**3**, *R*=3,5‐bis(t‐butyl)phenyl. B) SCXRD structures of [Co(**1**)](ClO_4_)_2_ (HS isomer), [Co(**2**)](ClO_4_)_2_ and [Co(**3**)](ClO_4_)_2_ in sticks representation with the coordination sphere of the de novo models superimposed in ball and stick. SCXRD colors as (A) except N dark blue, O red, Co black, H white. Anions and majority of H atoms omitted for clarity. Selected intercomponent interactions highlighted.

We compared our previously reported solid‐state structure of [Co(**1**)](ClO_4_)_2_ (see Figure 1 A for ligand structures) derived from single crystal X‐ray diffraction (SCXRD) analysis (Figure 1 B)^[22]^ with modelled structures generated de novo (Gaussian09,^[37]^ CAM‐B3LYP/6‐31G*/LAN2DZ[Co], see the Supporting Information for details). It should be noted that the rotaxane framework enforces the formation of a pseudo‐heteroleptic complex and prevents binding of additional ligands, to give a predictable, if relatively rare, 5‐coordinate all‐neutral N‐donor distorted square‐based pyramidal (sbpy)^[38]^ binding mode, which is not observed with the non‐interlocked ligands.

Two independent structures with different bond lengths (RMSD=0.12 Å, Table S1) were observed in the asymmetric unit of [Co(**1**)](ClO_4_)_2_, suggesting that high spin (HS) and low spin (LS) configurations co‐exist in the solid state. The coordination spheres of the de novo HS and LS models of [Co(**1**)]^2+^ agree remarkably well, both in terms of geometry and bond lengths (RMSD=0.05 and 0.04 Å respectively, Table S10), with one of the structures observed by SCXRD (Figure 1 B for the HS structure), supporting this proposal. Furthermore, a relatively small energetic preference for the HS configuration was predicted computationally for both the de novo structures (5.9 kJ mol^−1^) and models of [Co(**1**)]^2+^ derived from the corresponding SCXRD geometries (2.7 kJ mol^−1^, see the Supporting Information for details), although it should be noted that predicting accurate energy gaps of multiconfigurational complexes is a challenge for DFT modelling. Consistent with this, the EPR spectra of polycrystalline [Co(**1**)](ClO_4_)_2_ show that the complex exhibits both HS and LS configurations in the solid state (vide infra), in line with the EPR data previously obtained on frozen solutions.^[22]^

Having validated our de novo computational approach in the case of [Co(**1**)](ClO_4_)_2_, we modelled complexes based on interlocked ligands containing other readily available macrocycle components,^[39]^ one of which (**2**) is more rigid and the other (**3**) contains a potentially weakly coordinating ether unit near to the bipyridine ligand (Figure 1 A). [Co(**2**)]^2+^ was predicted to display an all‐neutral N, 5‐coordinate environment similar to that of [Co(**1**)](ClO_4_)_2_ but in this case, π‐Co and π‐π interactions result in distortion of the sbpy geometry and a twisting of the macrocycle relative to the axle (Figure 1 B). In the case of [Co(**3**)]^2+^, the weakly coordinating ether O was predicted to bind to the metal ion in lowest energy model obtained, resulting in a distorted‐octahedral geometry, although once again the N donors are arranged in a sbpy geometry (Figure 1 B).

Rotaxane‐based ligands **2** and **3** were synthesized from the respective macrocyles^[35]^ and alkyne and azide axle precursors using an active template[^40^, ^41^, ^42^] Cu‐mediated alkyne‐azide cycloaddition reaction[^43^, ^44^, ^45^, ^46^] and the corresponding Co^II^ complexes prepared (see the Supporting Information). The asymmetric units of the solid‐state structures obtained by SCXRD of [Co(**2**)](ClO_4_)_2_ and [Co(**3**)](ClO_4_)_2_ contain two very similar structures (bond length RMSD=0.007 and 0.002 Å respectively, Table S1). The SCXRD sbpy^[38]^ geometry of [Co(**2**)](ClO_4_)_2_ agrees well with the HS de novo model (bond length RMSD=0.02 Å, Table S10), with similar π‐Co and π‐π interactions observed in both, confirming that the modelling captures these inter‐component interactions with reasonable accuracy. Similarly, although the calculated and observed octahedral^[47]^ structures of [Co(**3**)](ClO_4_)_2_ differ to a greater degree (bond length RMSD=0.17 Å, Table S10), perhaps in part due to the more disordered nature of the SCXRD structure (Figure 1 B, Table S10), the modelling accurately predicts the observed weak but structurally important Co‐O interaction. Both the de novo models of [Co(**2**)]^2+^ and [Co(**3**)]^2+^ (10.9 and 18 kJ mol^−1^, respectively) and models constructed from the SCXRD coordinates (46.0 and 11.8 kJ mol^−1^, respectively) suggest a larger HS‐LS gap than in the case of [Co(**1**)]^2+^, consistent with a single geometry being observed in their SCXRD structures.

The agreement between de novo models of [Co(**1**–**3**)]^2+^ and their SCXRD‐derived structures confirms that the predictable nature of the coordination environment provided by a mechanically chelating ligand, combined with the ability of simple computational models to accurately capture weak, geometry distorting interactions, allows the structure of such complexes to be predicted with reasonable precision. The computationally predicted values of *D* obtained for the de novo HS models of [Co(**1**–**3**)](ClO_4_)_2_ agree remarkably well with models derived from the SCXRD geometries (Table 1) and, excitingly, large negative values of *D* were predicted for all three complexes. Thus, we turned to the experimental evaluation of the magnetic properties of [Co(**1**–**3**)](ClO_4_)_2_.

**Table 1:** Experimental and calculated (SA‐5‐CASSCF[7,5]) parameters for the HS states of Co(**1**–**3**)](ClO_4_)_2_.

	[Co(**1**)]^2+^	[Co(**2**)]^2+^	[Co(**3**)]^2+^
Calculated values			
*D* [cm^−1^] de novo	−80.0	−71.2	−80.0
*D* [cm^−1^] *SCXRD‐derived*	−65.3	−58.1	−95.9
Experimental values			
*D*^[a]^ [cm^−1^]	−78	−59	−95
HS:LS populations^[a]^	0.55:0.45^[b]^	0.95:0.05^[c]^	0.90:0.10^[c]^
*g*_1_^[d]^	2.02	2.42	1.70
*g*_2_^[d]^	2.40	2.00	2.33
*g*_3_^[d]^	2.56	2.67	2.56
*E*/*D*^[d]^	0.20	0.11	0.22
*g*_av_^[d]^	2.33	2.36	2.20
*g*_av_^[a]^	2.33	2.31	2.33
*U*_eff_ [cm^−1^]^[e]^	156	118	190
*τ*_0_ [s]	3.4×10^−5^	9.8×10^−3^	1.1×10^−5^

**Figure 2:**
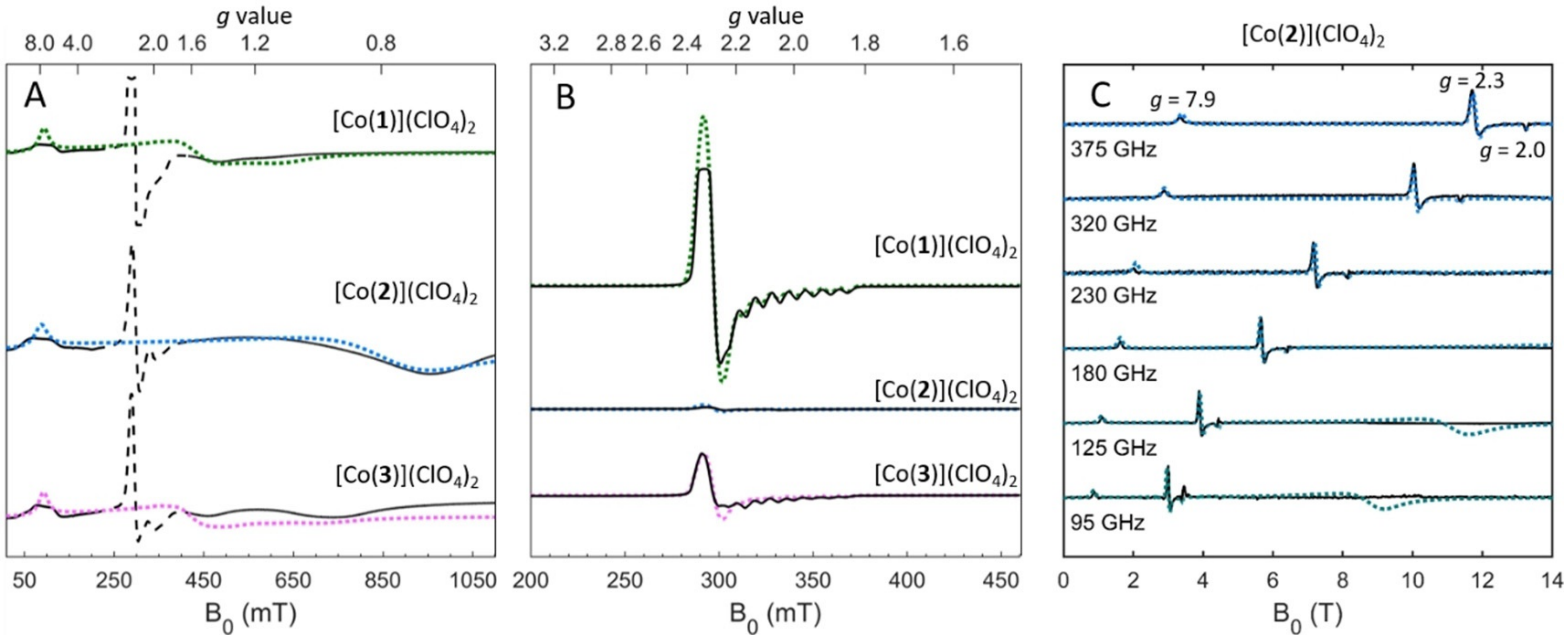
Summary of EPR spectroscopic data. Experimental data in black, simulated data in green, blue and magenta for [Co(**1**–**3**)](ClO_4_)_2_, respectively. X‐band data at 10 K (A) and 100 K (B). In (A) LS species are shown as dashed lines as they are saturated at 10 K. C) High‐field EPR spectra and simulated data for [Co(**2**)](ClO_4_)_2_ at 4.5 K. Simulations for the HS states in (A) and (C) were performed with PHI^[51]^ with parameters listed in Table 1. Simulation parameters for the LS states (B) are given in Supporting Information (Table S3).

[a] Determined by static magnetic data. [b] Varies with temperature, values obtained from magnetization vs. H measurements at 2–7 K (Figure S26). [c] First estimated from the magnetization vs. H at 2–7 K but does not vary with temperature. [d] Determined by EPR for the high‐spin *S*=3/2 state. High‐spin EPR signals in Figure 2 A were simulated using *D* obtained from magnetic data. For a definition of the SCXRD‐derived models see Figure 5 and accompanying text. [e] *U*_eff_ values (2*D*) were calculated from experimentally obtained *D*.

Powders and frozen‐solutions of [Co(**1**–**3**)](ClO_4_)_2_ were investigated to evaluate their spin configurations and provide an estimate of *D*. X‐band EPR spectra of polycrystalline samples at 10 K (Figure 2 A) display features characteristic of both LS (dotted signals at ca. 300 mT) and HS (solid black lines) configurations. This signal assignment is confirmed by the persistence of the LS signal at 100 K (Figure 2 B) at which the HS species relaxes too fast to be detectable. The difference in the LS signal intensities in **1**–**3** at 100 K is in keeping with the calculated HS‐LS energy gap and unambiguously points to a ligand effect on the spin state population.

The LS species exhibit similar *g* values and hyperfine splitting for all complexes, where an eight‐line hyperfine splitting on *g*_z_, due to the interaction of the unpaired electron with the Co^II^ nucleus (*I*=7/2), is visible (Figure 2 B, see Table S3 for simulation parameters). The 10 K EPR spectra (Figure 2 A) span a very broad field range, indicating significant magnetic anisotropy in the HS state, for which two distinct sets of signals are visible in all three complexes. Although the transition at low fields occurs at the same effective *g* value[^48^, ^49^] (*g*_eff_=7.9) in all complexes, the high‐field signals are dependent on the nature of the ligand. The signal at *g*_eff_=7.9 must arise from a transition within the *M*_s_=±3/2 doublet, characterized by *g*_z_ >2.5,^[50]^ demonstrating that *D* is negative (see the Supporting Information). EPR spectra of frozen solutions (Figure S23) were similar to those of polycrystalline samples, showing that no coupling occurs in the solid state, presumably due to the steric bulk of the ligands preventing close approach of the Co^II^ ions.

**Figure 3:**
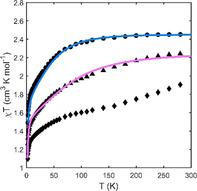
Plot of *χT* vs. T for [Co(**1**)](ClO_4_)_2_ (⧫), [Co(**2**)](ClO_4_)_2_ (•), [Co(**3**)](ClO_4_)_2_ (▴) at 1000 Oe (100 mT). Solid lines show fits obtained with *PHI*. No fit is provided for **1** due to the change in spin population with temperature (see Figure S26 for fit of magnetization vs. H).

To determine the magnitude of *D*, high‐field EPR (HFEPR) measurements were carried out on pelletized samples at 4.5 K (Figure 3 C for [Co(**2**)](ClO_4_)_2_, Figure S24 for [Co(**1**)](ClO_4_)_2_ and [Co(**3**)](ClO_4_)_2_). The absence of additional EPR lines in the HFEPR spectra shows that no inter‐doublet transitions occur up to 375 GHz, setting |*D*|≥15 cm^−1^ but preventing accurate determination of *D*. The large (negative) *D* value is also responsible for the absence of *M*_s_=±1/2 intra‐doublet signals. The assignment of the *g*_eff_=7.9 signal to an intra‐Kramer transition is confirmed by the linear trend of the peak positions in the entire experimental frequency range (Figure S24D).

The EPR data show that the ligand environment not only influences the population of the LS state, with [Co(**1**)](ClO_4_)_2_ ≫ [Co(**3**)](ClO_4_)_2_ > [Co(**2**)](ClO_4_)_2_, as predicted by modelling, but that it affects the coordination environment of the HS state, in line with the SCXRD data. Importantly, the EPR data demonstrate experimentally that the HS configuration in complexes [Co(**1**–**3**)](ClO_4_)_2_ exhibits a large negative zero‐field splitting (*D*<−15 cm^−1^), in line with predictions for the de novo models.

The static magnetic properties of [Co(**1**–**3**)](ClO_4_)_2_ were assessed by direct current (DC) susceptibility and magnetization measurements on the pelletized samples previously measured by HFEPR (Figure 3 and Figures S25–27). The measured room temperature *χT* values of 1.90, 2.45, 2.24 cm^3^ K mol^−1^ for [Co(**1**–**3**)](ClO_4_)_2_, respectively, are higher than the value (*χT*=1.88 cm^3^ K mol^−1^) expected for *S*=3/2 systems with *g*=2 in the spin‐only approximation, but lie in the typical range for five‐coordinated Co^II^ complexes with second‐order SOC.[^52^, ^53^] The magnitude and thermal dependence of *χT* (Figure 3) for [Co(**2**–**3**)](ClO_4_)_2_ confirms the HS state as the dominant state of these complexes, and the slightly smaller *χT* measured for **3** compared to **2** is consistent with the higher amount of LS present in this sample (Figure 2 B).

For [Co(**1**)](ClO_4_)_2_, the *χT* vs. *T* data point to an incomplete spin‐crossover (SCO) behaviour, with a transition from a mixture of states at low T to a progressive conversion to the HS state at *T*>100 K, suggesting that the LS configuration is lower in energy. Although the modelling suggests that the HS state is favored, it should be noted that the calculated energy gap HS‐LS is small (2.7 kJ mol^−1^) and was obtained in the gas phase and is thus inconclusive. Although many 4‐ and 6‐coordinate Co^II^ complexes are known to undergo a SCO transition, including reversible switching between SCO and SIM,^[54]^ 5‐coordinate Co^II^ SCO compounds are rare, especially in conjunction with SIM behaviour,^[55]^ making [Co(**1**)](ClO_4_)_2_ only the second SCO complex characterized by a neutral CoN_5_ coordination.^[56]^

The susceptibility decrease at low temperatures was attributed to magnetic anisotropy rather than antiferromagnetic impurities or interactions between the spins (in agreement with EPR analysis),^[57]^ as the field dependent magnetization data collected at 100 K show a perfectly linear trend (Figure S25). The reduced magnetization plots (Figure S26) also indicate magnetic anisotropy due to the absence of a single master curve.[^58^, ^59^]

To determine the magnetic parameters of [Co(**1**–**3**)](ClO_4_)_2_, the *χT* vs. *T* plots (Figure 3) and the magnetization vs. *H* at multiple temperatures (Figure S26) were simultaneously fitted with PHI.^[51]^ The best fits yielded *g*_average_ of 2.33, 2.31 and 2.33 and *D* values of −78, −59 and −95 cm^−1^ for [Co(**1**–**3**)](ClO_4_)_2_, respectively (Table 1, see also Figure S27 for fits with different *D* values), considerably larger than values obtained for other pentacoordinate mononuclear Co^II^ complexes[^60^, ^61^, ^62^] (Table S4). Because the spin population varies with temperature for [Co(**1**)](ClO_4_)_2_, only the fit of the magnetization was used to estimate the parameters (i.e. the *χT* vs. T plot was not fitted for [Co(**1**)](ClO_4_)_2_ in Figure 3), and the best fits were obtained by including a contribution of the LS species at 0.45, 0.05 and 0.1 for [Co(**1**–**3**)](ClO_4_)_2_, respectively (Table 1). Comparison of solution and solid‐state EPR, the magnetization data at 100 K and solution susceptibility values (2.05, 2.56 and 2.29 cm^3^ K mol^−1^ for [Co(**1**–**3**)](ClO_4_)_2_, respectively, see the Supporting Information), all indicate that no *J* coupling needs to be considered. In contrast to EPR, the inclusion of the rhombic zero‐field parameter *E* in the magnetic data did not lead to a better fit, (due to the lower sensitivity of SQUID measurements). The large negative *D* revealed by DC measurements for [Co(**1**–**3**)](ClO_4_)_2_ (Table 1), which are in good agreement with the values obtained from X‐band and HFEPR simulations, and the variability of *D* with the rotaxane framework thus justify their further investigation as potential “tuneable” SIMs.

**Figure 4:**
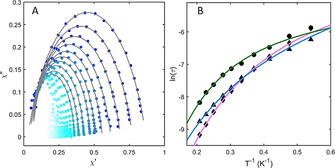
A) Frequency dependence of the out‐of‐phase magnetic susceptibility of **2** at 1.8–10 K under an applied field of 250 mT, with fits in grey. See Figure S31 for a complete set of data for **1**–**3**. (B) Temperature dependence of the relaxation times (*τ* for complexes **1** (⧫), **2** (•) and **3** (▴) with fits (solid lines). See the Supporting Information and Table S8 for fitting parameters.

The frequency dependence of the in‐phase (χ′) and the out‐of‐phase (χ′′) magnetic susceptibility for [Co(**1**–**3**)](ClO_4_)_2_ was measured in the temperature range 1.8–10 K under an oscillating (1–1500 Hz) field of 1.55 Oe (0.155 mT). In the absence of an external static field, none of the complexes displayed maxima in the out‐of‐phase susceptibility. Application of a static field reduces the quantum tunneling of magnetization (QTM) effect by removing the degeneracy between microstates and thus out‐of‐phase maxima for all complexes are observed (Figure S28–S30). These shift to low frequency with the increase in the magnetic field strength, reaching a maximum shift under an applied field of 100–250 mT, considered to be the optimal field under which to observe the slow magnetic relaxation. Strongly temperature and frequency dependent ac susceptibility signals characteristic of single ion magnet (SIM) behaviour were observed in all cases (Figure S31). This characteristic field‐induced SIM behaviour, displayed by all complexes, is exemplified for [Co(**2**)](ClO_4_)_2_ in Figure 4 A.

The fitting of the *χ*′ and *χ*′′ data (Figure 4 A and S31) with the extended Debye model^[63]^ (see the Supporting Information) yielded magnetization relaxation times (*τ*) and their distribution (*α*) at each temperature for [Co(**1**–**3**)](ClO_4_)_2_ (Tables S5–7). Reliable fits of the AC magnetic data were obtained for the temperature range between 1.8 and 4.8 K. The *α* values range between 0.16–0.30, 0.07–0.25 and 0.07–0.25 for [Co(**1**–**3**)](ClO_4_)_2_, respectively, showing a narrow distribution of the relaxation times. The curvature of the plot of ln(*τ*) vs. *T* ^−1^ (Figure 4 B) points to the presence of several relaxation pathways that cannot be fitted with an Orbach process alone. Contributions from additional relaxation pathways were therefore considered and the relaxation times fitted using to Equation 1 (see Table S8 for parameters):

(1) $$\tau^{-1}=aT+bT^{n}+\tau_{0}^{-1}e^{-Ueff/kT},$$

where the first term designates the direct process, the second the Raman process and the third the Orbach process; QTM was set to zero given its dependence on *H* ^−2^ (*H*=magnetic field).^[64]^

We fixed the energy barrier to the theoretically expected value of 2*D* using the experimentally obtained *D* (224 K (156 cm^−1^), 170 K (118 cm^−1^) and 273 K (190 cm^−1^) for [Co(**1**–**3**)](ClO_4_)_2_, respectively; all parameters obtained from the fitting are summarized in Table 1). For such large zero‐field splittings, the *D*‐values derived from dc magnetic measurements are quite reliable, resulting in reasonable fits of the data, with minimal contribution from the Orbach process (Figure S32). The direct process was considered in the fitting, due to the dependence of the direct coupling between the |+3/2⟩ and |−3/2⟩ states to *H* ^4^.[^64^, ^65^] The exponent of the Raman mechanism *n* was set as fit parameter and values in the range from 2.9–4.0 were obtained; even though *n*=9 is predicted for Kramers doublets in extended lattices whose dynamics are well described by the Debye model, much lower values have been reported for molecular compounds[^57^, ^66^, ^67^] due to the importance of optical phonons (essentially molecular vibrations) to the relaxation process.^[68]^ Fixing *n*=9 did not lead to satisfactory fits of the data.

**Figure 5:**
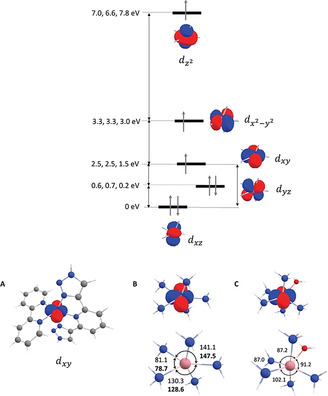
Splitting of the *d*‐orbitals obtained at the SA‐5‐CASSCF(7,5)/TZVP level of theory for the models with the NH_3_ ligands. The energies of the orbitals are shown for [Co(**1**)](ClO_4_)_2_ [Co(**2**)](ClO_4_)_2_ and [Co(**3**)](ClO_4_)_2_. A) The orientation of the *d*_xy_ orbital in the rotaxane framework. B) *d*_xy_ orbital and experimental angles for [Co(**1**–**2**)](ClO_4_)_2_. C) *d*_xy_ orbital and experimental angles for [Co(**3**)](ClO_4_)_2_. Note that the orbitals shown are CASSCF orbitals rather than pure *d*‐orbitals; the assignment of the *d*‐character is based on the *d* orbital with the largest contribution to the natural orbital.

To understand the origin of the variation in *D* and hence energy barrier to relaxation in complexes [Co(**1**–**3**)](ClO_4_)_2_, calculations using the experimental geometries from SCXRD data yielded the splitting of the *d*‐orbital energy levels (Figure 5). Energy levels were determined using state average CASSCF, implemented in Orca,^[69]^ taking into account relativistic and SOC effects with an active space considering 7 electrons and 5 roots in the state averaging (SA‐5‐CASSCF[7,5]).^[70]^

To probe the role of the rotaxane framework in determining *D*, we optimized the simplified model of the axle and macrocycle fragment common to the three molecules (Figure 5 A). The *D* term obtained for this moiety is −93.9 cm^−1^, a large negative value close to the experimental value for [Co(**1**–**3**)](ClO_4_)_2_. We further simplified the model to reflect a purely conformational constraint; the rotaxane framework was replaced by NH_3_ ligands (and one molecule of H_2_O in the case of **3**), with the ligand‐metal distances and related angles constrained to those obtained by SCXRD. Unsurprisingly, given the equivalent symmetry of their primary coordination sphere (Figure 1), all three complexes exhibit similar *d*‐orbital splittings (Figure 5). Comparable splitting diagrams have been reported for other distorted sbpy and elongated octahedral Co^II^ complexes that display large and negative *D* values.[^32^, ^36^, ^53^, ^71^] The calculated zero‐field splittings for these SCXRD‐derived models are in excellent agreement with experiments (Table 1), reproducing both the trend in |*D*| ([Co(**3**)](ClO_4_)_2_ > [Co(**1**)](ClO_4_)_2_ > [Co(**2**)](ClO_4_)_2_) and giving values in close to quantitative agreement with the experimental data for the complexes derived from **2** and **3**. Even the de novo models gave predicted values of *D* in good qualitative agreement with those observed (Table 1). These calculations show that the rotaxane framework itself is not directly relevant in determining *D*, but rather serves to impose a geometrically constrained environment on the metal ion.

Computational modelling allows the differences observed experimentally between the complexes to be rationalized. Large negative values of *D* can be obtained due to the presence of significant SOC‐induced mixing of low‐lying excited states with the ground state. This is because the *D* tensor terms ($D_{kl}$ ) depend on the matrix elements of the angular momentum operator, the effective SOCs $\left(\xi \right)$ and the energy gap between the first excited state (Q_1_) and the ground state (Q_0_) with $D_{kl}\propto \;-\frac{\xi^{2}}{{E(Q}_{1})-E\left(Q_{0}\right)}$ .[^60^, ^71^] Our calculations show that the transition from Q_0_ to Q_1_ represents the largest contribution to *D* for all complexes. With relatively similar SOC average matrix elements between Q_1_ and Q_0_ of 325, 305 and 295 cm^−1^ and considerably different Q_1_‐Q_0_ energy gaps (obtained with NEVPT2) of 790, 1169 and 415 cm^−1^ for [Co(**1**–**3**)](ClO_4_)_2,_ respectively, it is evident that the latter largely determines *D*. Further analysis illustrates how the geometrical restrictions enforced by the rotaxane scaffold influences the electronic properties of the metal ion and so modulate *D*.

For the three complexes, Q_0_ is highly multiconfigurational (see the Supporting Information), the electronic configuration shown in Figure 5 (*d_xz_*)^2^(*d_yz_*)^2^(*d_xy_*)^1^($d_{x^{2}-y^{2}}$ )^1^($d_{z^{2}}$ )^1^_,_ contributes only 50 % to Q_0_ for [Co(**1**–**2**)](ClO_4_)_2_. In contrast for [Co(**3**)](ClO_4_)_2_, the electronic configuration (*d_xz_*)^2^(*d_yz_*)^1^(*d_xy_*)^2^($d_{x^{2}-y^{2}}$ )^1^($d_{z^{2}}$ )^1^ represents 52 % of Q_0_. Q_1_ is dominated by the electronic configuration (*d_xz_*)^1^(*d_yz_*)^2^(*d_xy_*)^2^($d_{x^{2}-y^{2}}$ )^1^($d_{z^{2}}$ )^1^ with contributions of 69 %, 76 % and 60 % for [Co(**1**–**3**)](ClO_4_)_2_. The *d_xy_* orbital thus has a significant population in the excited state and mainly controls the Q_1_‐Q_0_ gap. The stability of the *d*_xy_ orbitals is determined by the intramolecular interactions in the *xy* plane (Figure 5). For [Co(**1**–**2**)](ClO_4_)_2_, one of the *d* lobes is projected along a Co−N bond, increasing the energy of the *d*_xy_ orbital. Both complexes display similar angles and the energies of their *d_yz_* orbitals are similar (2.5 eV). The smaller Q_1_‐Q_0_ gap in [Co(**1**)](ClO_4_)_2_ in comparison with complex [Co(**2**)](ClO_4_)_2_ is due to a relatively large contribution of 20 % from the lowest energy configuration to Q_1_ for the former in contrast with only 7 % for the latter. For complex [Co(**3**)](ClO_4_)_2,_ the N‐Co‐N angles are closer to 90° and the *d*_xy_ orbital does not significantly overlap with any of the Co−N bonds (Figure 5). Consequently, the *d*_xy_ orbital is more stable in [Co(**3**)](ClO_4_)_2_ resulting in a significantly smaller Q_1_‐Q_0_ gap and a more negative *D* term.

Our calculations reveal the interplay between the geometry of the frameworks and the electronic structure in tuning the *D* value. For rotaxanes with similar SOCs, imposing geometrical constrains to control the stability of the *d*_xy_ orbital could be an effective strategy to tune the value of *D*.

## Conclusion

The results presented support the proposal that interlocked molecules can provide a ligand platform for the development of SIMs and that computational modelling can be used to direct this process. Although hybrid organic‐inorganic rotaxanes have been proposed as qubits,^[72]^ complexes [Co(**1**–**3**)](ClO_4_)_2_ are the first examples to show field‐induced SIM behaviour. Our combined EPR and magnetic measurement approach enabled us to determine the magnetic parameters, including the rhombic anisotropy (*E*/*D*=0.10–0.22) and HS:LS populations, and the magnitude of *D*, with confidence. [Co(**1**–**3**)](ClO_4_)_2_ exhibit remarkably large negative *D* values compared to other penta‐coordinated cobalt complexes reported (summarized in Table S4) that exhibit SIM behaviour, such as bis(imino)pyridine pincer cobalt complexes^[52]^ (*D*=−28 cm^−1^) and [Co(phen)(DMSO)Cl_2_]^[73]^ (*D*≈−17 cm^−1^). Although [Co(**1**–**3**)](ClO_4_)_2_ all exhibited slow relaxation of the magnetization in the presence of a magnetic field, there is clearly room for improvement in tuning the structures to achieve SIM behaviour at higher temperature and in the absence of a magnetic field.

We have shown that the zero‐field splittings may be predicted computationally with reasonable accuracy even using de novo models, thanks to the predictable coordination environment provided by the mechanical bond. Magnetostructural correlations with computational methods previously revealed that the size of *D* may be tuned through structural changes in [Co^II^(tbta)N_3_]^+^ complexes by varying the Lewis basicity of the axial ligand on the N_3_^−^ site.^[60]^ For the Co^II^ rotaxane‐based complexes presented here, our calculations suggest a new strategy to tune *D*, based on the control of the intramolecular angles that determine the stability and population of (in this case) the *d*_xy_ orbital. We have shown that the HS‐LS energy gap may be estimated even using simple DFT de novo models, although it should be noted that higher levels of theory are required to determine *D* in these complexes exhibiting multiconfigurational ground states. Using CASSCF, good agreement between calculated and experimentally obtained *D* values could be reached with the SCXRD‐derived truncated models, demonstrating that the enforced coordination geometry is more important than the exact chemical structure of the ligands. As we have recently demonstrated,^[22]^ the mechanical bond can enforce unusual coordination environments, suggesting there is scope for further ligand engineering. We are now investigating interlocked SIMs with larger total spin values and no nuclear spin to eliminate hyperfine coupling and so improve their efficiency.

## Conflict of interest

The authors declare no conflict of interest.

## Supporting information

As a service to our authors and readers, this journal provides supporting information supplied by the authors. Such materials are peer reviewed and may be re‐organized for online delivery, but are not copy‐edited or typeset. Technical support issues arising from supporting information (other than missing files) should be addressed to the authors.

Supplementary

Supplementary
