# Supplementary material for: Rotaxane CoII Complexes as Field‐Induced Single‐Ion Magnets
Source: Angew Chem Int Ed Engl. 2021 Jun 14;60(29):16051–8. doi: 10.1002/anie.202103596 (PMC8361961; doi:10.1002/anie.202103596)

checkCIF/PLATON report

Structure factors have been supplied for datablock(s) 2019gjt0001x

THIS REPORT IS FOR GUIDANCE ONLY. IF USED AS PART OF A REVIEW PROCEDURE FOR PUBLICATION, IT SHOULD NOT REPLACE THE EXPERTISE OF AN EXPERIENCED CRYSTALLOGRAPHIC REFEREE.

No syntax errors found. CIF dictionary Interpreting this report

Datablock: 2019gjt0001x

```
Bond precision:  C-C = 0.0073 A          Wavelength=1.54178

Cell:        a=16.0634(2)        b=21.8864(4)       c=25.3399(4)
             alpha=111.5424(16)  beta=101.7314(14)  gamma=95.4570(14)
Temperature: 100 K
```

	Calculated	Reported
Volume	7971.2(2)	7971.1(2)
Space group	P -1	P -1
Hall group	-P 1	-P 1
Moiety formula	C67 H77 Co N9 O5, Cl O4 [+ solvent]	C67 H77 Co N9 O5, Cl O4, 1[CLO4- + SOLVENT]
Sum formula	C67 H77 Cl Co N9 O9 [+ solvent]	C67 H77 Cl Co N9 O9
Mr	1246.76	1246.75
Dx,g cm-3	1.039	1.039
Z	4	4
Mu (mm-1)	2.409	2.409
F000	2632.0	2632.0
F000’	2631.34	
h,k,lmax	19,26,31	19,26,30
Nref	30588	29545
Tmin,Tmax	0.749,0.845	0.710,1.000
Tmin’	0.633	

```
Correction method= # Reported T Limits: Tmin=0.710 Tmax=1.000
AbsCorr = GAUSSIAN

Data completeness= 0.966          Theta(max)= 70.646

R(reflections)= 0.0871( 22277)    wR2(reflections)= 0.2634( 29545)

S = 1.043                  Npar= 1869
```

The following ALERTS were generated. Each ALERT has the format
test-name_ALERT_alert-type_alert-level.
Click on the hyperlinks for more details of the test.

Alert level B

```
PLAT220_ALERT_2_B NonSolvent   Resd 1  C   Ueq(max)/Ueq(min) Range        8.1 Ratio
PLAT242_ALERT_2_B Low    'MainMol' Ueq as Compared to Neighbors of       C04G Check
```

Alert level C

```
PLAT084_ALERT_3_C High wR2 Value (i.e. > 0.25) ...................       0.26 Report
PLAT213_ALERT_2_C Atom C6              has ADP max/min Ratio .....        3.1 prolat
PLAT213_ALERT_2_C Atom C04R            has ADP max/min Ratio .....        3.5 prolat
PLAT213_ALERT_2_C Atom C04T            has ADP max/min Ratio .....        3.4 prolat
PLAT213_ALERT_2_C Atom C02I            has ADP max/min Ratio .....        3.5 prolat
PLAT213_ALERT_2_C Atom C04S            has ADP max/min Ratio .....        3.7 prolat
PLAT221_ALERT_2_C Solv./Anion  Resd 2  C   Ueq(max)/Ueq(min) Range        5.6 Ratio
PLAT222_ALERT_3_C NonSolvent Resd 1  H   Uiso(max)/Uiso(min) Range        9.3 Ratio
PLAT223_ALERT_4_C Solv./Anion  Resd 2  H   Ueq(max)/Ueq(min) Range        7.0 Ratio
PLAT230_ALERT_2_C Hirshfeld Test Diff for    N00T     --N00W     .        5.5 s.u.
PLAT234_ALERT_4_C Large Hirshfeld Difference C04G     --C04Z     .       0.18 Ang.
PLAT241_ALERT_2_C High   'MainMol' Ueq as Compared to Neighbors of       O01J Check
PLAT242_ALERT_2_C Low    'MainMol' Ueq as Compared to Neighbors of       C03A Check
PLAT242_ALERT_2_C Low    'MainMol' Ueq as Compared to Neighbors of       C03N Check
PLAT260_ALERT_2_C Large Average Ueq of Residue Including      Cl00      0.172 Check
PLAT341_ALERT_3_C Low Bond Precision on  C-C Bonds ...............    0.00733 Ang.
PLAT410_ALERT_2_C Short Intra H...H Contact  H01E     ..H02A     .       1.99 Ang.
                                                      x,y,z  =      1_555 Check
PLAT430_ALERT_2_C Short Inter D...A Contact  O04B     ..N00K     .       2.90 Ang.
                                                      x,y,z  =      1_555 Check
PLAT911_ALERT_3_C Missing FCF Refl Between Thmin & STh/L=    0.600        316 Report
PLAT976_ALERT_2_C Check Calcd Resid. Dens.  0.76A   From O02W           -0.40 eA-3
```

Alert level G

```
FORMU01_ALERT_1_G  There is a discrepancy between the atom counts in the
            _chemical_formula_sum and _chemical_formula_moiety. This is
            usually due to the moiety formula being in the wrong format.
            Atom count from _chemical_formula_sum:   C67 H77 Cl1 Co1 N9 O9
            Atom count from _chemical_formula_moiety:C67 H77 Cl2 Co1 N9 O9 Os1
PLAT002_ALERT_2_G Number of Distance or Angle Restraints on AtSite         66 Note
PLAT072_ALERT_2_G SHELXL First  Parameter in WGHT  Unusually Large       0.15 Report
PLAT083_ALERT_2_G SHELXL Second Parameter in WGHT  Unusually Large       7.54 Why ?
PLAT176_ALERT_4_G The CIF-Embedded .res File Contains SADI Records         56 Report
PLAT187_ALERT_4_G The CIF-Embedded .res File Contains RIGU Records          2 Report
PLAT232_ALERT_2_G Hirshfeld Test Diff (M-X)  Co02     --N00S     .        5.2 s.u.
PLAT232_ALERT_2_G Hirshfeld Test Diff (M-X)  Co02     --N00U     .        5.4 s.u.
PLAT244_ALERT_4_G Low    'Solvent' Ueq as Compared to Neighbors of       Cl03 Check
PLAT244_ALERT_4_G Low    'Solvent' Ueq as Compared to Neighbors of       Cl00 Check
PLAT301_ALERT_3_G Main Residue  Disorder ..............(Resd  1  )        23% Note
PLAT302_ALERT_4_G Anion/Solvent/Minor-Residue Disorder (Resd  2  )        12% Note
PLAT410_ALERT_2_G Short Intra H...H Contact  H5       ..H16      .       1.77 Ang.
                                                      x,y,z  =      1_555 Check
PLAT412_ALERT_2_G Short Intra XH3 .. XHn     H05E     ..H04C     .       1.76 Ang.
                                                      x,y,z  =      1_555 Check
PLAT412_ALERT_2_G Short Intra XH3 .. XHn     H01O     ..H13      .       2.10 Ang.
                                                      x,y,z  =      1_555 Check
PLAT412_ALERT_2_G Short Intra XH3 .. XHn     H01O     ..H2C      .       1.85 Ang.
                                                      x,y,z  =      1_555 Check
PLAT412_ALERT_2_G Short Intra XH3 .. XHn     H02S     ..Ht       .       2.13 Ang.
```

```
                                                     x,y,z  =      1_555 Check
PLAT412_ALERT_2_G Short Intra XH3 .. XHn     H02S     ..H22      .       2.07 Ang.
                                                     x,y,z  =      1_555 Check
PLAT413_ALERT_2_G Short Inter XH3 .. XHn     H16D     ..H05I     .       2.05 Ang.
                                                   x,y,1+z  =      1_556 Check
PLAT606_ALERT_4_G Solvent Accessible VOID(S) in Structure ........          ! Info
PLAT720_ALERT_4_G Number of Unusual/Non-Standard Labels ..........        327 Note
PLAT794_ALERT_5_G Tentative Bond Valency for Co01      (II)      .       1.76 Info
PLAT794_ALERT_5_G Tentative Bond Valency for Co02      (II)      .       1.77 Info
PLAT802_ALERT_4_G CIF Input Record(s) with more than 80 Characters          3 Info
PLAT860_ALERT_3_G Number of Least-Squares Restraints .............       1893 Note
PLAT910_ALERT_3_G Missing # of FCF Reflection(s) Below Theta(Min).          4 Note
PLAT912_ALERT_4_G Missing # of FCF Reflections Above STh/L=  0.600        723 Note
PLAT933_ALERT_2_G Number of OMIT Records in Embedded .res File ...         27 Note
PLAT941_ALERT_3_G Average HKL Measurement Multiplicity ...........        4.5 Low
PLAT978_ALERT_2_G Number C-C Bonds with Positive Residual Density.          0 Info
```

```
   0 ALERT level A = Most likely a serious problem - resolve or explain
   2 ALERT level B = A potentially serious problem, consider carefully
  20 ALERT level C = Check. Ensure it is not caused by an omission or oversight
  30 ALERT level G = General information/check it is not something unexpected

   1 ALERT type 1 CIF construction/syntax error, inconsistent or missing data
  30 ALERT type 2 Indicator that the structure model may be wrong or deficient
   8 ALERT type 3 Indicator that the structure quality may be low
  11 ALERT type 4 Improvement, methodology, query or suggestion
   2 ALERT type 5 Informative message, check
```

It is advisable to attempt to resolve as many as possible of the alerts in all categories. Often the minor alerts point to easily fixed oversights, errors and omissions in your CIF or refinement strategy, so attention to these fine details can be worthwhile. In order to resolve some of the more serious problems it may be necessary to carry out additional measurements or structure refinements. However, the purpose of your study may justify the reported deviations and the more serious of these should normally be commented upon in the discussion or experimental section of a paper or in the "special_details" fields of the CIF. checkCIF was carefully designed to identify outliers and unusual parameters, but every test has its limitations and alerts that are not important in a particular case may appear. Conversely, the absence of alerts does not guarantee there are no aspects of the results needing attention. It is up to the individual to critically assess their own results and, if necessary, seek expert advice.

Publication of your CIF in IUCr journals

A basic structural check has been run on your CIF. These basic checks will be run on all CIFs submitted for publication in IUCr journals (*Acta Crystallographica*, *Journal of Applied Crystallography*, *Journal of Synchrotron Radiation*); however, if you intend to submit to *Acta Crystallographica Section C* or *E* or *IUCrData*, you should make sure that full publication checks are run on the final version of your CIF prior to submission.

Publication of your CIF in other journals

Please refer to the *Notes for Authors* of the relevant journal for any special instructions relating to CIF submission.

PLATON version of 18/09/2020; check.def file version of 20/08/2020

Datablock 2019gjt0001x - ellipsoid plot

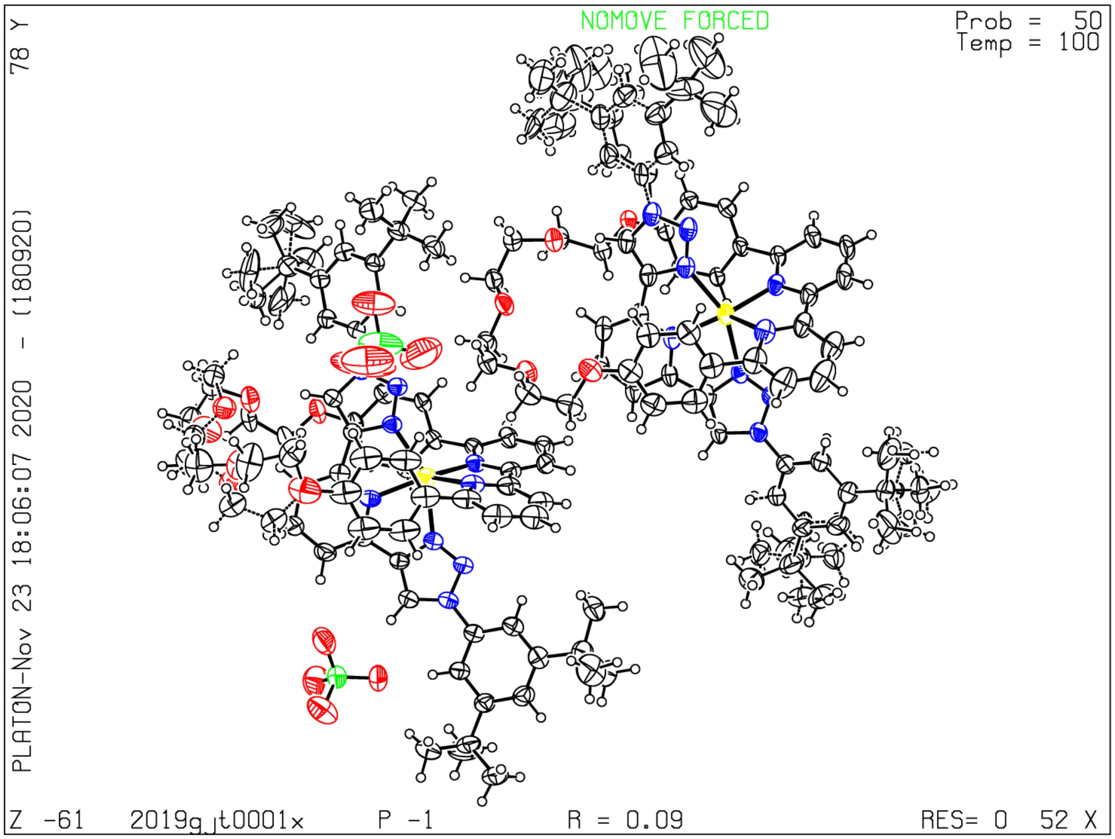

Supplement: Supplementary file 1 — Supplementary [file ANIE-60-16051-s001.zip › [Co(2)]2+(ClO4-)2_checkcif.pdf]
