# Supplementary material for: Rotaxane CoII Complexes as Field‐Induced Single‐Ion Magnets
Source: Angew Chem Int Ed Engl. 2021 Jun 14;60(29):16051–8. doi: 10.1002/anie.202103596 (PMC8361961; doi:10.1002/anie.202103596)

checkCIF/PLATON report

Structure factors have been supplied for datablock(s) 2019gjt0002v

THIS REPORT IS FOR GUIDANCE ONLY. IF USED AS PART OF A REVIEW PROCEDURE FOR PUBLICATION, IT SHOULD NOT REPLACE THE EXPERTISE OF AN EXPERIENCED CRYSTALLOGRAPHIC REFEREE.

No syntax errors found. CIF dictionary Interpreting this report

Datablock: 2019gjt0002v

```
Bond precision:  C-C = 0.0095 A          Wavelength=1.54184

Cell:           a=16.2078(7)      b=20.0541(7)     c=21.1503(9)
                alpha=88.396(3)   beta=71.007(4)   gamma=85.105(3)
Temperature:    100 K

                 Calculated                Reported
Volume           6476.6(5)                 6476.6(5)
Space group      P -1                      P -1
Hall group       -P 1                      -P 1
Moiety formula   C67 H77 Co N9 O4, 2(Cl O4)C67 H77 Co N9 O4, 2(Cl O4)
Sum formula      C67 H77 Cl2 Co N9 O12     C67 H77 Cl2 Co N9 O12
Mr               1330.21                   1330.20
Dx,g cm-3        1.364                     1.364
Z                4                         4
Mu (mm-1)        3.407                     3.407
F000             2796.0                    2796.0
F000'            2797.40
h,k,lmax         19,24,25                  19,24,25
Nref             23746                     23549
Tmin,Tmax        0.849,0.903               0.861,0.987
Tmin'            0.788

Correction method= # Reported T Limits: Tmin=0.861 Tmax=0.987
AbsCorr = GAUSSIAN

Data completeness= 0.992          Theta(max)= 68.249

R(reflections)= 0.0878( 12543)    wR2(reflections)= 0.2845( 23549)

S = 1.004                  Npar= 1844
```

The following ALERTS were generated. Each ALERT has the format
test-name_ALERT_alert-type_alert-level.
Click on the hyperlinks for more details of the test.

Alert level C

```
RINTA01_ALERT_3_C  The value of Rint is greater than 0.12
            Rint given   0.156
PLAT020_ALERT_3_C The Value of Rint is Greater Than 0.12 .........      0.156 Report
PLAT084_ALERT_3_C High wR2 Value (i.e. > 0.25) ...................       0.28 Report
PLAT112_ALERT_2_C ADDSYM Detects New (Pseudo) Symm. Elem       b/2         84 %Fit
PLAT213_ALERT_2_C Atom C04M            has ADP max/min Ratio .....        3.2 prolat
PLAT213_ALERT_2_C Atom O00{            has ADP max/min Ratio .....        3.2 prolat
PLAT213_ALERT_2_C Atom C2              has ADP max/min Ratio .....        3.8 prolat
PLAT220_ALERT_2_C NonSolvent   Resd 1  C   Ueq(max)/Ueq(min) Range        3.4 Ratio
PLAT220_ALERT_2_C NonSolvent   Resd 2  C   Ueq(max)/Ueq(min) Range        3.6 Ratio
PLAT230_ALERT_2_C Hirshfeld Test Diff for    C03D     --C041     .        6.0 s.u.
PLAT230_ALERT_2_C Hirshfeld Test Diff for    C03Z     --C04A     .        5.9 s.u.
PLAT230_ALERT_2_C Hirshfeld Test Diff for    C04U     --C04X     .        5.3 s.u.
PLAT234_ALERT_4_C Large Hirshfeld Difference C02T     --C02Y     .       0.16 Ang.
PLAT234_ALERT_4_C Large Hirshfeld Difference C034     --C1A      .       0.24 Ang.
PLAT234_ALERT_4_C Large Hirshfeld Difference C02U     --C052     .       0.17 Ang.
PLAT234_ALERT_4_C Large Hirshfeld Difference C04C     --C04L     .       0.16 Ang.
PLAT234_ALERT_4_C Large Hirshfeld Difference C04F     --C04I     .       0.17 Ang.
PLAT234_ALERT_4_C Large Hirshfeld Difference C04T     --C04X     .       0.22 Ang.
PLAT241_ALERT_2_C High   'MainMol' Ueq as Compared to Neighbors of       C04J Check
PLAT241_ALERT_2_C High   'MainMol' Ueq as Compared to Neighbors of       C04A Check
PLAT241_ALERT_2_C High   'MainMol' Ueq as Compared to Neighbors of       C04S Check
PLAT241_ALERT_2_C High   'MainMol' Ueq as Compared to Neighbors of       C04U Check
PLAT242_ALERT_2_C Low    'MainMol' Ueq as Compared to Neighbors of       C03M Check
PLAT242_ALERT_2_C Low    'MainMol' Ueq as Compared to Neighbors of       O00B Check
PLAT242_ALERT_2_C Low    'MainMol' Ueq as Compared to Neighbors of       C02Q Check
PLAT242_ALERT_2_C Low    'MainMol' Ueq as Compared to Neighbors of       C02U Check
PLAT242_ALERT_2_C Low    'MainMol' Ueq as Compared to Neighbors of       C04H Check
PLAT341_ALERT_3_C Low Bond Precision on  C-C Bonds ...............    0.00952 Ang.
PLAT790_ALERT_4_C Centre of Gravity not Within Unit Cell: Resd.  #          1 Note
              C67 H77 Co N9 O4
PLAT906_ALERT_3_C Large K Value in the Analysis of Variance ......      2.075 Check
PLAT911_ALERT_3_C Missing FCF Refl Between Thmin & STh/L=    0.600        155 Report
```

Alert level G

```
PLAT002_ALERT_2_G Number of Distance or Angle Restraints on AtSite         35 Note
PLAT072_ALERT_2_G SHELXL First  Parameter in WGHT  Unusually Large       0.15 Report
PLAT176_ALERT_4_G The CIF-Embedded .res File Contains SADI Records         31 Report
PLAT187_ALERT_4_G The CIF-Embedded .res File Contains RIGU Records          1 Report
PLAT244_ALERT_4_G Low    'Solvent' Ueq as Compared to Neighbors of       Cl03 Check
PLAT244_ALERT_4_G Low    'Solvent' Ueq as Compared to Neighbors of       Cl04 Check
PLAT244_ALERT_4_G Low    'Solvent' Ueq as Compared to Neighbors of       Cl05 Check
PLAT244_ALERT_4_G Low    'Solvent' Ueq as Compared to Neighbors of       Cl06 Check
PLAT301_ALERT_3_G Main Residue  Disorder ..............(Resd  1  )        17% Note
PLAT301_ALERT_3_G Main Residue  Disorder ..............(Resd  2  )         6% Note
PLAT398_ALERT_2_G Deviating  C-O-C    Angle From 120 for O0              99.8 Degree
PLAT398_ALERT_2_G Deviating  C-O-C    Angle From 120 for O016            99.0 Degree
PLAT398_ALERT_2_G Deviating  C-O-C    Angle From 120 for O00{            97.5 Degree
PLAT410_ALERT_2_G Short Intra H...H Contact  H11      ..H04P     .       2.09 Ang.
                                                      x,y,z  =     1_555 Check
PLAT410_ALERT_2_G Short Intra H...H Contact  H19      ..H7B      .       1.62 Ang.
                                                      x,y,z  =     1_555 Check
PLAT411_ALERT_2_G Short Inter H...H Contact  H6       ..H04      .       2.02 Ang.
                                                      x,y,z  =     1_555 Check
PLAT412_ALERT_2_G Short Intra XH3 .. XHn     H01Q     ..H04Z     .       2.01 Ang.
                                                      x,y,z  =     1_555 Check
PLAT412_ALERT_2_G Short Intra XH3 .. XHn     H028     ..H0B      .       2.09 Ang.
                                                      x,y,z  =     1_555 Check
PLAT413_ALERT_2_G Short Inter XH3 .. XHn     Hy       ..H1AA     .       1.95 Ang.
```

```
                                                     x,y,z  =      1_555 Check
PLAT432_ALERT_2_G Short Inter X...Y Contact  C11      ..C03A            3.11 Ang.
                                                   1+x,y,z  =      1_655 Check
PLAT720_ALERT_4_G Number of Unusual/Non-Standard Labels ..........       316 Note
PLAT773_ALERT_2_G Check long C-C Bond in CIF: C034     --C1A            1.72 Ang.
PLAT860_ALERT_3_G Number of Least-Squares Restraints .............       233 Note
PLAT912_ALERT_4_G Missing # of FCF Reflections Above STh/L=  0.600        43 Note
PLAT933_ALERT_2_G Number of OMIT Records in Embedded .res File ...         1 Note
PLAT978_ALERT_2_G Number C-C Bonds with Positive Residual Density.         0 Info
```

```
  0 ALERT level A = Most likely a serious problem - resolve or explain
  0 ALERT level B = A potentially serious problem, consider carefully
 31 ALERT level C = Check. Ensure it is not caused by an omission or oversight
 26 ALERT level G = General information/check it is not something unexpected

  0 ALERT type 1 CIF construction/syntax error, inconsistent or missing data
 33 ALERT type 2 Indicator that the structure model may be wrong or deficient
  9 ALERT type 3 Indicator that the structure quality may be low
 15 ALERT type 4 Improvement, methodology, query or suggestion
  0 ALERT type 5 Informative message, check
```

It is advisable to attempt to resolve as many as possible of the alerts in all categories. Often the minor alerts point to easily fixed oversights, errors and omissions in your CIF or refinement strategy, so attention to these fine details can be worthwhile. In order to resolve some of the more serious problems it may be necessary to carry out additional measurements or structure refinements. However, the purpose of your study may justify the reported deviations and the more serious of these should normally be commented upon in the discussion or experimental section of a paper or in the "special_details" fields of the CIF. checkCIF was carefully designed to identify outliers and unusual parameters, but every test has its limitations and alerts that are not important in a particular case may appear. Conversely, the absence of alerts does not guarantee there are no aspects of the results needing attention. It is up to the individual to critically assess their own results and, if necessary, seek expert advice.

Publication of your CIF in IUCr journals

A basic structural check has been run on your CIF. These basic checks will be run on all CIFs submitted for publication in IUCr journals (*Acta Crystallographica*, *Journal of Applied Crystallography*, *Journal of Synchrotron Radiation*); however, if you intend to submit to *Acta Crystallographica Section C* or *E* or *IUCrData*, you should make sure that full publication checks are run on the final version of your CIF prior to submission.

Publication of your CIF in other journals

Please refer to the *Notes for Authors* of the relevant journal for any special instructions relating to CIF submission.

PLATON version of 18/09/2020; check.def file version of 20/08/2020

Datablock 2019gjt0002v - ellipsoid plot

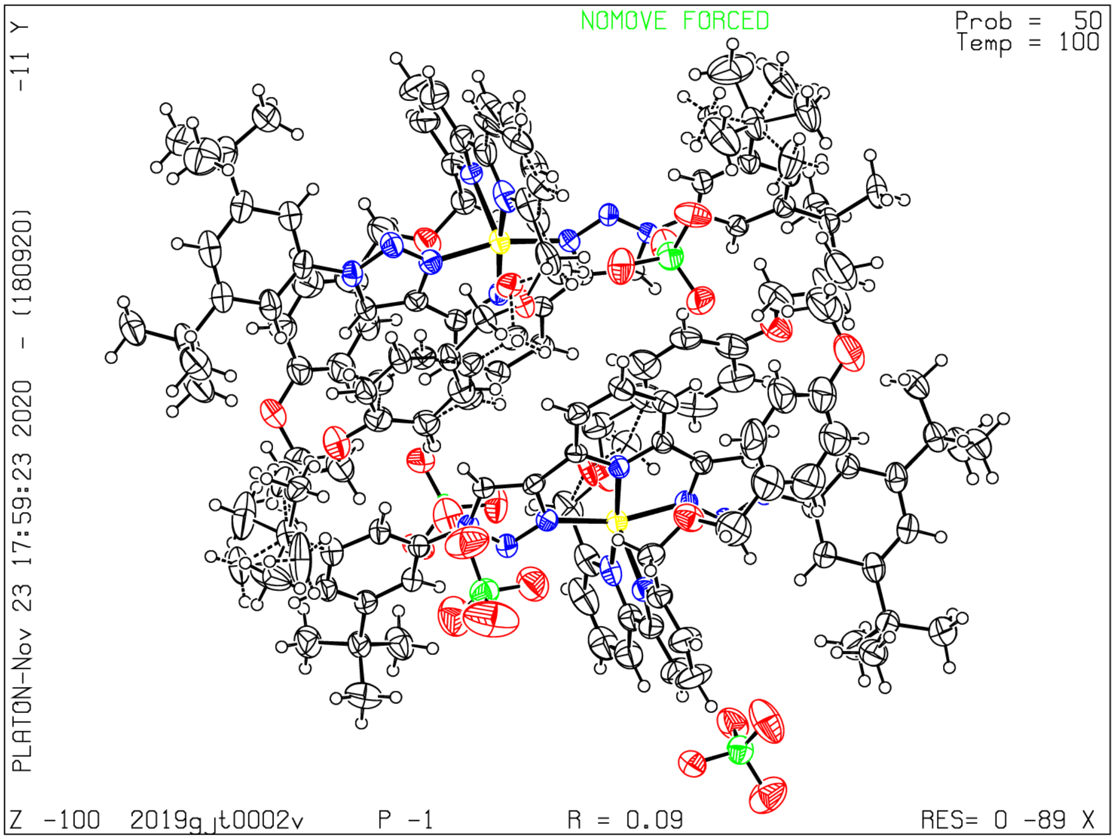

Supplement: Supplementary file 1 — Supplementary [file ANIE-60-16051-s001.zip › [Co(3)]2+(ClO4-)2_checkcif.pdf]
