# Supplementary material for: Rotaxane CoII Complexes as Field‐Induced Single‐Ion Magnets
Source: Angew Chem Int Ed Engl. 2021 Jun 14;60(29):16051–8. doi: 10.1002/anie.202103596 (PMC8361961; doi:10.1002/anie.202103596)
Supplement: Supplementary file 2 — Supplementary [file ANIE-60-16051-s002.pdf]

Angewandte Chemie
Eine Zeitschrift der Gesellschaft Deutscher Chemiker

Supporting Information

Rotaxane Co^{II} Complexes as Field-Induced Single-Ion Magnets

Martina Cirulli, Enrico Salvadori, Zhi-Hui Zhang, Michael Dommett, Floriana Tuna, Heiko Bamberger, James E. M. Lewis, Amanpreet Kaur, Graham J. Tizzard, Joris van Slageren, Rachel Crespo-Otero, Stephen M. Goldup,* and Maxie M. Roessler**

anie_202103596_sm_miscellaneous_information.pdf
anie_202103596_sm_cif.zip

Supporting Information

Contents

1 Synthesis of Rotaxane Compounds 3
1.1 General experimental procedures 3
1.2 Synthesis of rotaxane **2** 4
1.3 Synthesis of rotaxane **3** 7
1.4 General procedure for the preparation of Co^{II} complexes 10
1.5 Synthesis of complex [Co(**2**)]$(ClO_4)_2$ 10
1.6 Synthesis of complex [Co(**3**)]$(ClO_4)_2$ 11
2 Single-crystal X-ray Diffraction 12
2.1 X-ray crystallographic data for [Co(**1**)]$(ClO_4)_2$ 12
2.2 Single crystal X-ray crystallographic data for [Co(**2**)]$(ClO_4)_2$ 15
2.3 X-ray crystallographic data for [Co(**3**)]$(ClO_4)_2$ 18
2.4 Comparison between independent structures in the asymmetric units of [Co(**1-3**)]$(ClO_4)_2$ 21
3 EPR spectroscopy 22
3.1 EPR sample preparation 22
3.2 EPR measurements 22
3.3 EPR simulations 23
3.4 Frozen-solution X-band EPR spectra 24
3.5 Low-spin state EPR simulation parameters 24
3.6 Determination of negative zero-field splitting from high-spin EPR signals 25
3.7 HFEPR measurements 25

4 Magnetization ... 26

4.1 Sample preparation and magnetic measurements ... 26

4.2 Field-dependent magnetization measurements for [Co(**1-3**)](ClO_4)$_2$ ... 27

4.3 Assessing the magnitude of *D* for [Co(**2**)](ClO_4)$_2$ ... 29

4.4 Magnetic susceptibility from the Evan's method ... 29

4.5 Comparison of *D* with reported literature values ... 30

4.6 Field dependence of the magnetisation relaxation time extracted from ac susceptibility data at 2 K and several applied fields ... 31

4.7 Temperature dependence of the magnetic relaxation time extracted from ac susceptibility data recorded under the optimum applied field and fitting of the data ... 32

4.8 Fitting parameters for temperature-dependent relaxation (fixed U_{eff}) ... 36

5 Calculations ... 37

5.1 De novo modelling and comparison with the corresponding SCXRD structures ... 37

5.2 Comparison between the experimental SCXRD structures and optimised molecular models derived from them ... 38

5.3 Calculation of *D* values ... 42

1 Synthesis of Rotaxane Compounds

1.1 General experimental procedures

Unless otherwise stated, all reagents were purchased from commercial sources (Sigma Aldrich, Fisher Scientific, Alfa Aesar, Acros and Fluorochem) and used without further purification. [$Cu(MeCN)_4$][PF_6] was prepared as described by Pigorsch and Köckerling.[1] Anhydrous solvents were purchased from Acros. Experiments carried out in sealed vessels were performed in CEM microwave vials, with crimped caps, with PTFE septa. Unless otherwise stated, all reactions were carried out under an inert atmosphere of nitrogen. Flash column chromatography was performed using Biotage Isolera-4 or Isolera-1 automated chromatography system, employing Biotage SNAP or ZIP cartridges (50 µm, irregular silica, default flow rates). All azide waste was disposed of as described by Gardiner and co-workers.[2] Petrol refers to the fraction of petroleum ether boiling in the range 40-60 °C. TFA refers to trifluoroacetic acid. THF refers to tetrahydrofuran. DIPEA refers to *N,N*-diisopropylethylamine. Analytical TLC was performed on pre-coated silica gel plates on aluminum (0.25 mm thick, 60F254, Merck, Germany) and observed under UV light (254 nm). EDTA-NH_3 solution refers to an aqueous solution of NH_3 (17% w/w) saturated with sodium-ethylenediaminetetraacetate. All melting points were determined using a Griffin apparatus and are uncorrected. NMR spectra were recorded on Bruker AV400 or AV500 instrument, at a constant temperature of 298 K. Chemical shifts are reported in parts per million from low to high field and referenced to residual solvent. Coupling constants (*J*) are reported in Hertz (Hz). Standard abbreviations indicating multiplicity were used as follows: m = multiplet, quint = quintet, q = quartet, t = triplet, d = doublet, s = singlet, app. = apparent, br = broad, sept = septet. Signal assignment was carried out using 2D NMR methods (HSQC, HMBC, COSY, NOESY/ROESY, TOCSY etc) where necessary. In the case of some complex multiplets with contributions from more than proton signals, such as diastereoisomers, exact assignment was not possible. Here indicative either/or assignments (e.g. H_A or H_B) are provided. For clarity all proton signals corresponding to the axle components are in lower case, and all proton signals corresponding to the macrocycle components are in upper case. Low resolution mass spectrometry was carried out by the mass spectrometry services at University of Southampton (Waters TQD mass spectrometer equipped with a triple quadrupole analyser with UHPLC injection [BEH C_{18} column; MeCN-H_2O gradient {0.2% formic acid}]). High resolution mass spectrometry was carried out either by the mass spectrometry service at the University of Edinburgh (ThermoElectron MAT 900) or by the mass spectrometry services at the University of Southampton (MaXis, Bruker Daltonics, with a Time of Flight (TOF) analyser; samples were introduced to the mass spectrometer *via* a Dionex Ultimate 3000 autosampler and uHPLC pump in a gradient of 20% MeCN in hexane to 100% acetonitrile (0.2% formic acid) over 5-10 min at 0.6 mL/min; column: Acquity UPLC BEH C_{18} (Waters) 1.7 micron 50 × 2.1mm).

Macrocycles **M1**[3] and **M2**,[3] half axles **S1**[4] and **S2**,[5] rotaxane **1**,[6] complex [Co(**1**)](ClO_4)$_2$[6] were made according to literature procedures.

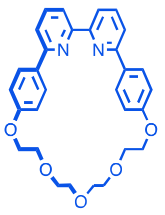

M1

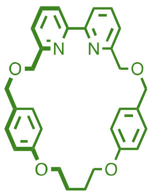

M2

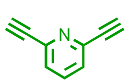

S1

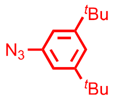

S2

1.2 Synthesis of rotaxane **2**

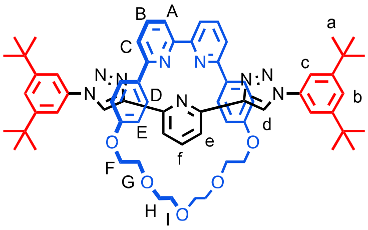

A solution of macrocycle **M1** (49.8, 0.10 mmol), azide **S2** (58.0 mg, 0.25 mmol) and bisalkyne pyridine **S1** (15.9 mg, 0.125 mmol) and $[Cu(MeCN)_4]PF_6$ (35.8 mg, 0.096 mmol) in CH_2Cl_2:EtOH (1 : 1, 2.5 mL) was added under N_2 to a sealed vial. A solution of DIPEA (70 μL, 0.4 mmol) was added to the reaction mixture and the red solution was stirred at 80 °C for 16 h. The solvent was removed *in vacuo*, the residue was dissolved in CH_2Cl_2 (7 mL) and 1 M EDTA/NH_3 (35 mL) was added, and the mixture was left stirring for 2 days. The phases were separated, and the aqueous phase was extracted with CH_2Cl_2 (3 × 7 mL). The combined organic phases were dried ($MgSO_4$) and the solvent removed in vacuo. The crude residue was purified by chromatography (gradient from 1 : 0 to 0 : 1 petrol-Et_2O with 1% NEt_3 throughout) to give rotaxane **2** as a pale-yellow foam (50 mg, 46%). ^1H NMR (400 MHz, $CDCl_3$) δ 9.20 (s, 1H, H_d), 7.82 (d, *J* = 7.8 Hz, 2H, H_A), 7.53 (m, 3H, H_B +H_f), 7.35 – 7.28 (m, 8H, H_C + H_b +H_c), 7.26 (d, *J* = 7.6 Hz, 2H, H_e), 7.16 – 7.13 (d, *J* = 8 Hz, 4H, H_E), 6.17 – 6.14 (d, *J* = 8 Hz, 4H, H_D), 3.96 – 3.80 (m, 12H, H_F +H_H +H_I), 3.56 (t, *J* = 6.0 Hz, 4H, H_G), 1.17 (s, 36H, H_a). ^{13}C NMR (101 MHz, $CDCl_3$) δ 159.7, 158.7, 157.4, 152.1, 149.7, 148.1, 137.1, 136.8, 136.6, 132.3, 128.9, 123.8, 122.3, 119.8, 119.1, 118.9, 115.1, 114.1, 69.8, 69.8, 68.5, 66.5, 35.0, 31.3. ESI-MS m/z = 1088.6 $[M+H]^+$ (calc. 1088.6 for $[C_{67}H_{77}N_9O_5]^+$).

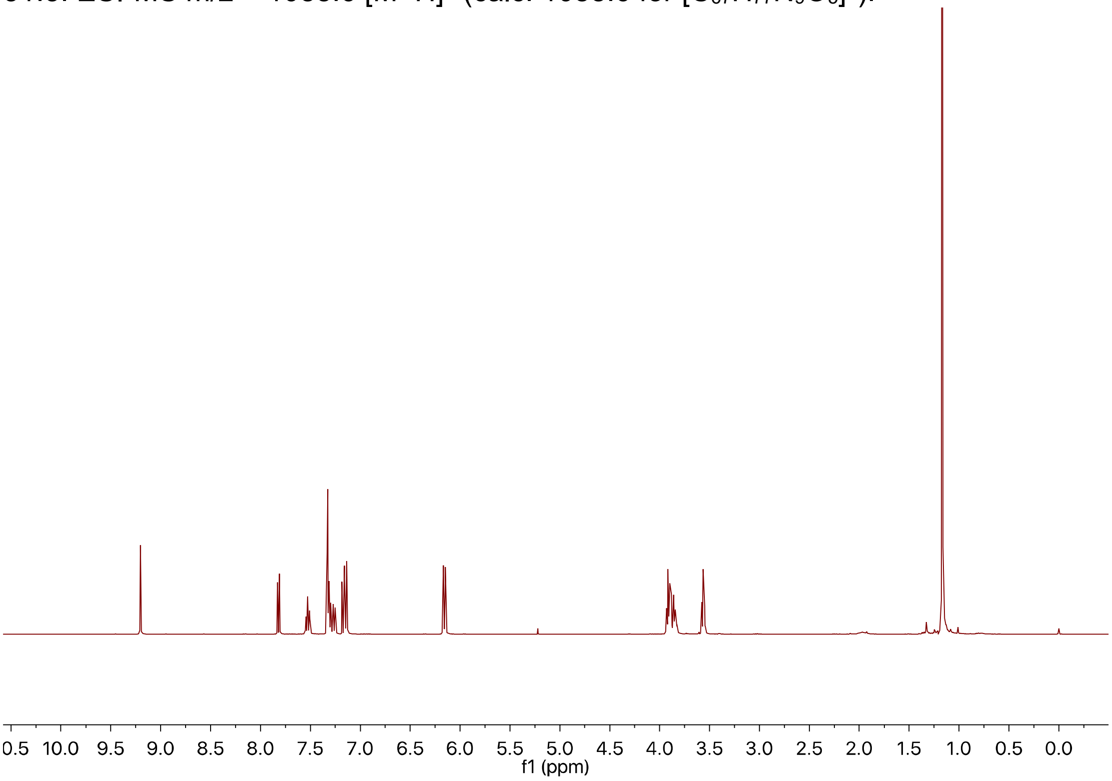

Figure S1. ^1H NMR ($CDCl_3$, 400 MHz) of **2**

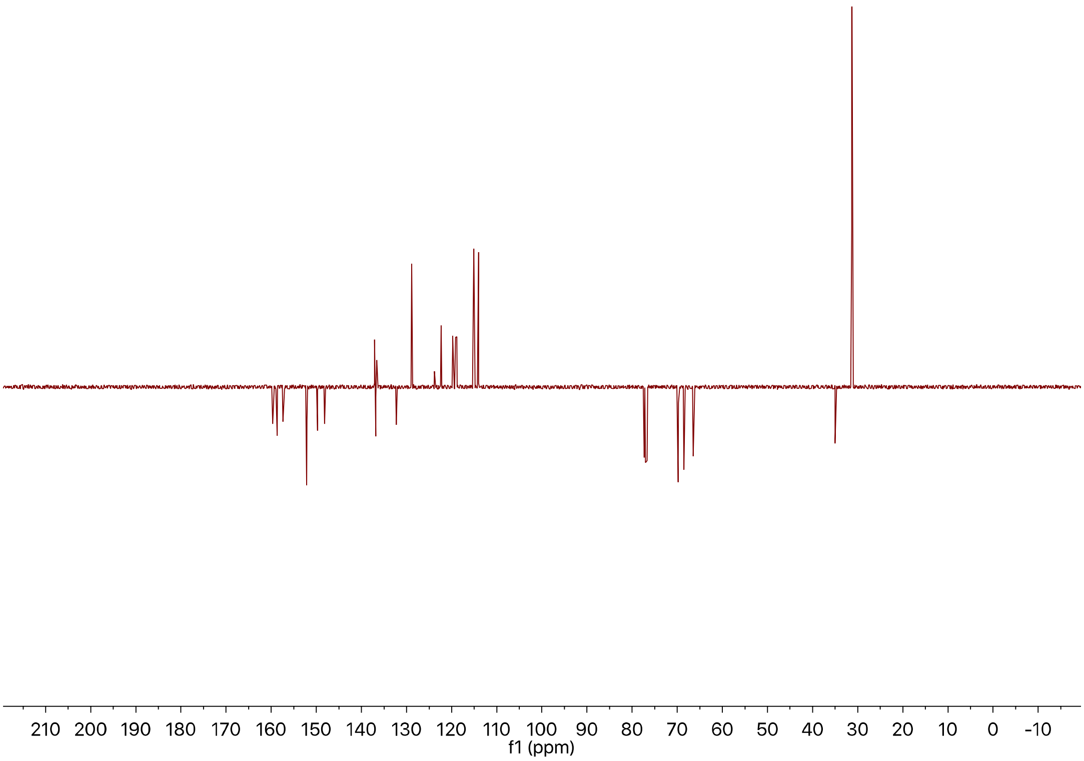

Figure S2. ^{13}C NMR ($CDCl_3$, 126 MHz) of **2**

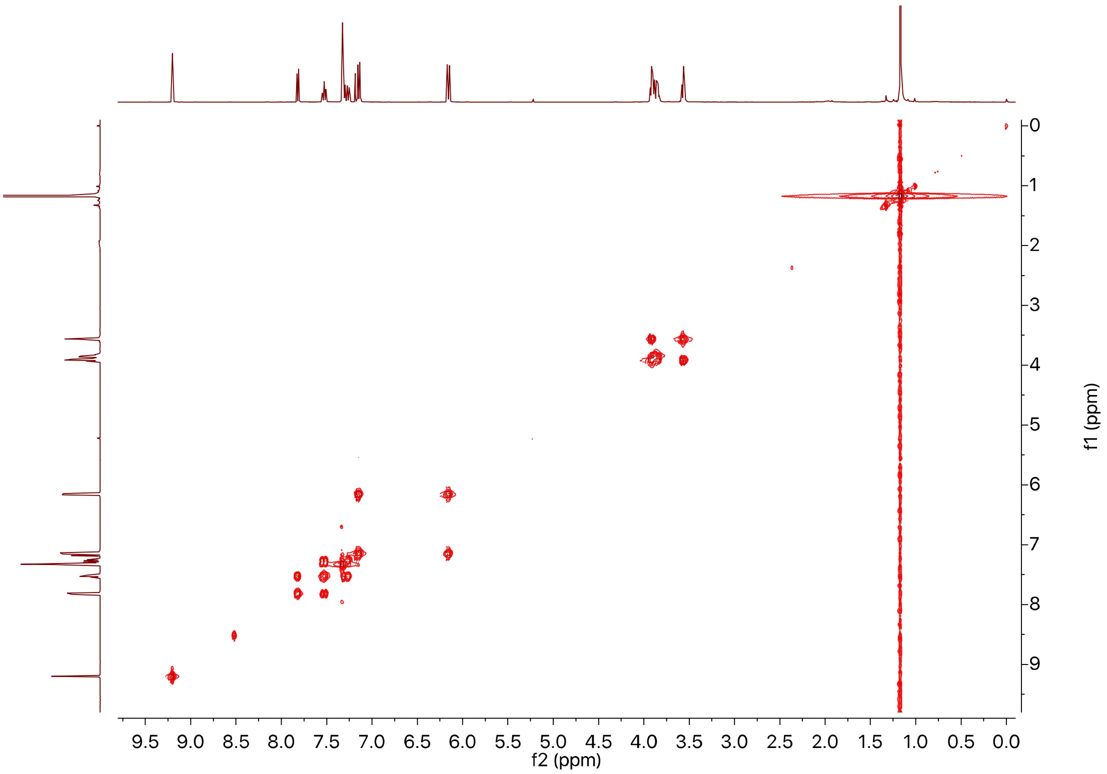

Figure S3. COSY NMR ($CDCl_3$) of **2**

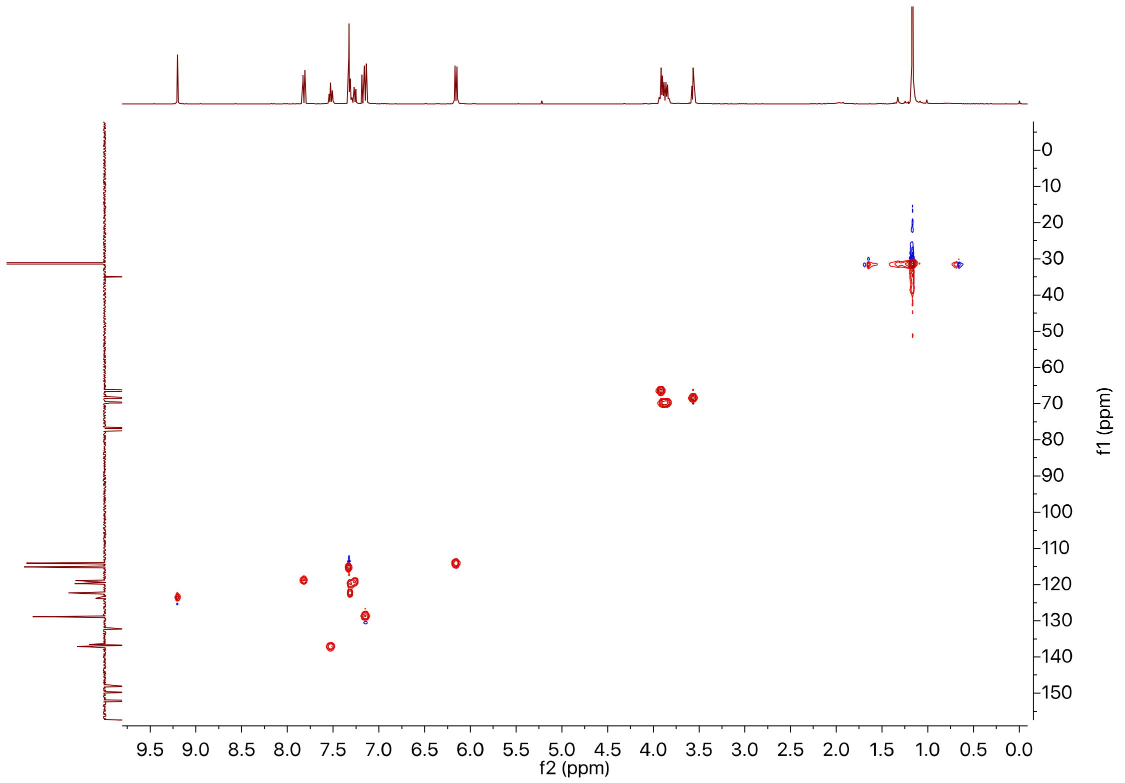

Figure S4. HSQC NMR ($CDCl_3$) of **2**

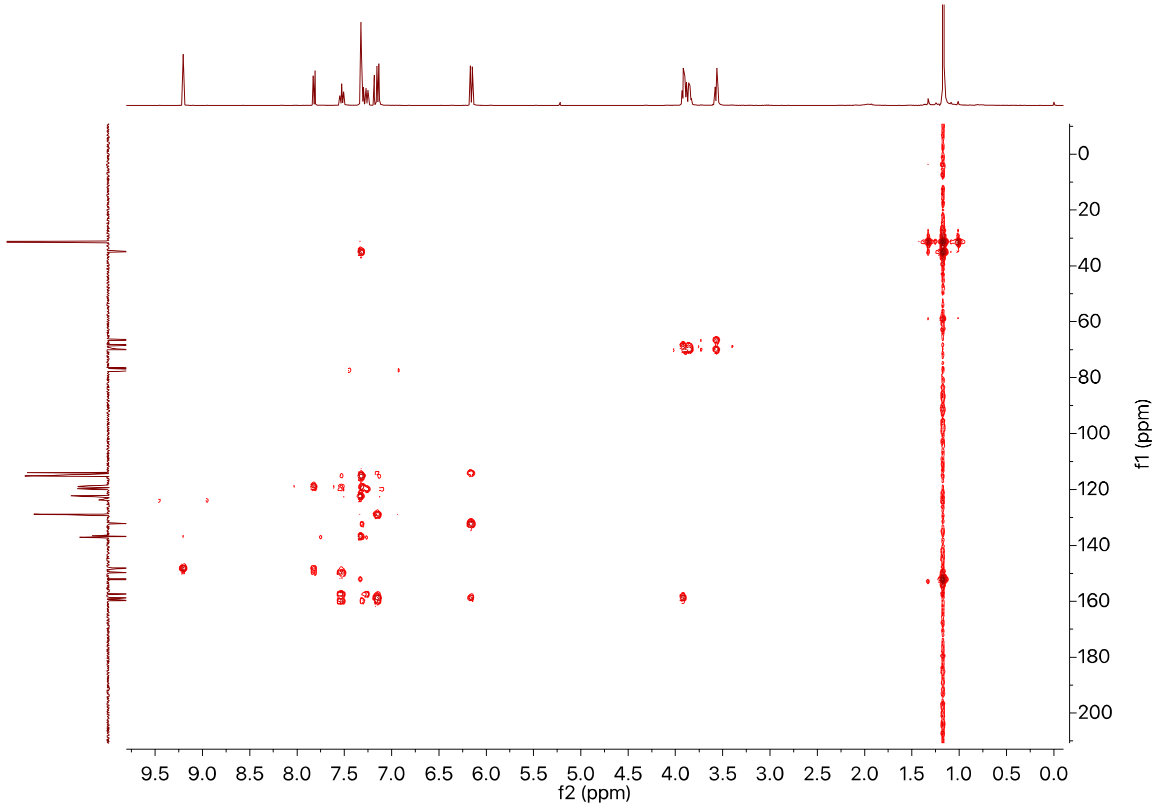

Figure S5. HMBC NMR ($CDCl_3$) of 2

1.3 Synthesis of rotaxane **3**

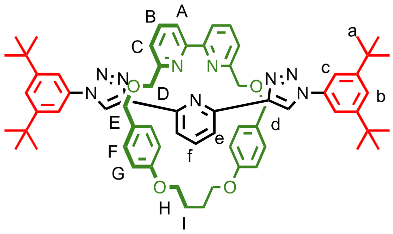

A solution of macrocycle **M2** (12.0, 0025 mmol), azide **S2** (14.3 mg, 0.062 mmol) and bisalkyne pyridine **S1** (3.94 mg, 0.031 mmol) and $[Cu(MeCN)_4]PF_6$ (8.94 mg, 0.024 mmol) in CH_2Cl_2:EtOH (1 : 1, 2 mL) was added under N_2 to a sealed vial. A solution of DIPEA (17 µL, 0.1 mmol) was added to the reaction mixture and the red solution was stirred at 80 °C for 18 h. The solvent was removed in vacuo, the residue was dissolved in CH_2Cl_2 (5 mL) and 1 M EDTA/NH_3 (50 mL) was added, and the mixture was left stirring overnight. The phases were separated, and the aqueous phase was extracted with CH_2Cl_2 (20 mL). The combined organic phases were dried (MgSO4) and the solvent removed in vacuo. The crude residue was purified by chromatography (gradient from 1 : 0 to 0 : 1 petrol-Et_2O with 1% NEt_3 throughout) to give rotaxane **3** as a pale-yellow foam (23 mg, 86%). ^1H NMR (400 MHz, Chloroform-*d*) δ 9.26 (s, 2H, H_d), 7.75 (d, *J* = 7.7 Hz, 2H, H_e), 7.61 (dd, *J* = 8.2, 7.4 Hz, 1H, H_f), 7.42 (d, *J* = 1.7 Hz, 4H, H_c), 7.34 – 7.28 (m, 4H, H_b + H_B), 7.25 (d, *J* = 7.1 Hz, 2H, H_C), 7.16 (d, *J* = 7.4 Hz, 2H, H_A), 6.63 (d, *J* = 8.6 Hz, 4H, H_F), 6.45 (d, *J* = 8.6 Hz, 4H, H_G), 4.43 (s, 4H, H_H), 4.28 (s, 4H, H_E), 3.82 (s, 4H, H_D), 2.19 (s, 4H, H_I), 1.21 (s, 36H, H_a). ^{13}C NMR (101 MHz, $CDCl_3$) δ 159.3, 159.2, 155.4, 151.9, 150.1, 147.8, 137.1, 136.8, 136.4, 129.2, 127.9, 123.3, 122.0, 120.9, 119.9, 118.9, 115.8, 115.0, 73.0, 70.2, 66.5, 35.1, 31.4, 24.9. ESI-MS m/z = 1072.6 $[M+H]^+$ (calc. 1072.6 for $[C_{67}H_{78}N_9O_4]^+$).

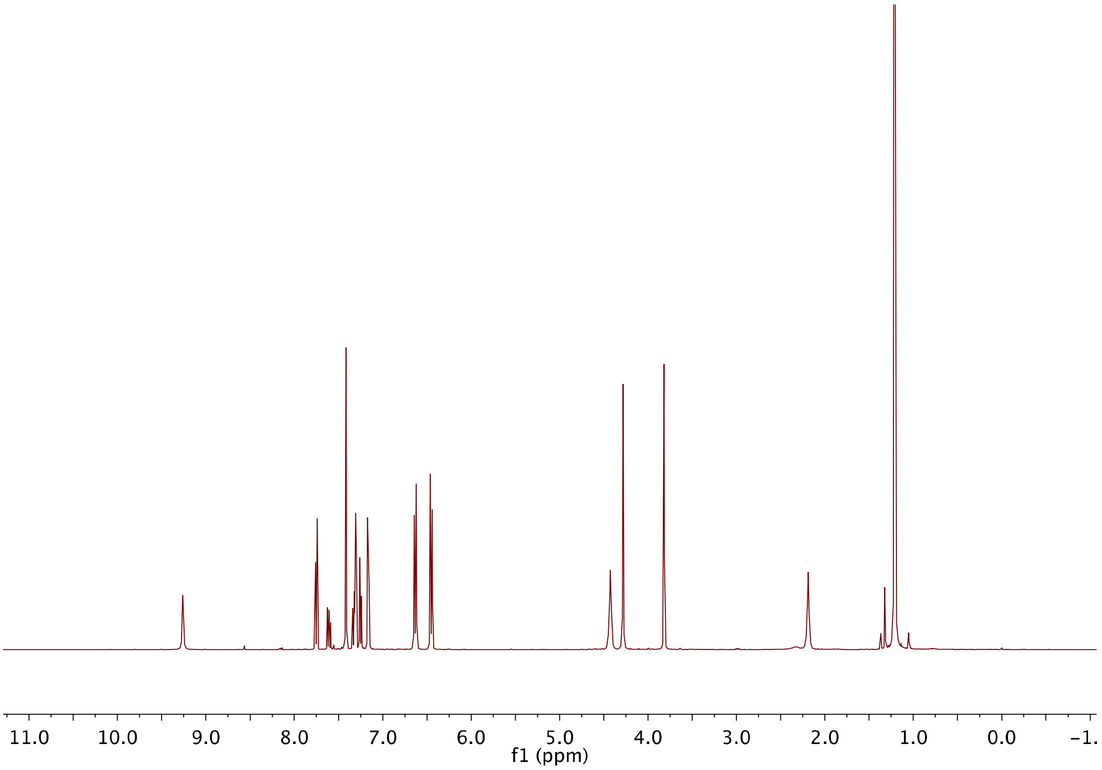

Figure S6. ^1H NMR ($CDCl_3$, 400 MHz) of **3**

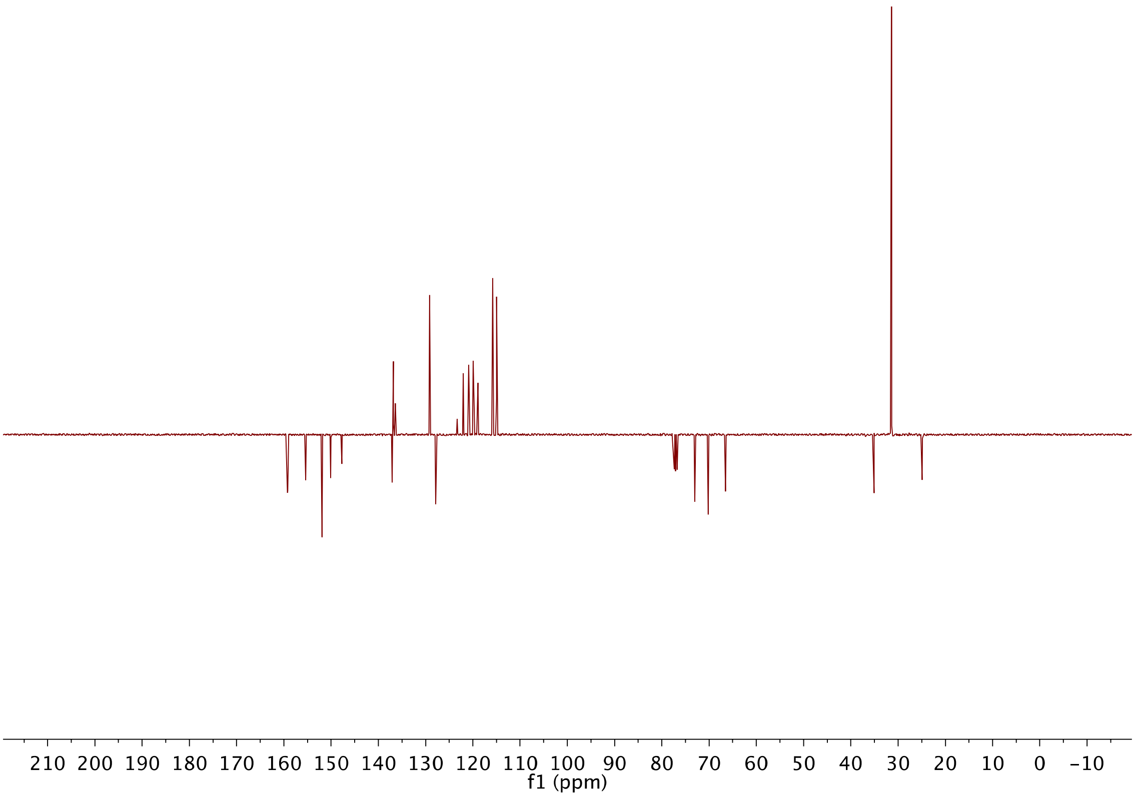

Figure S7. ^{13}C NMR ($CDCl_3$, 126 MHz) of **3**

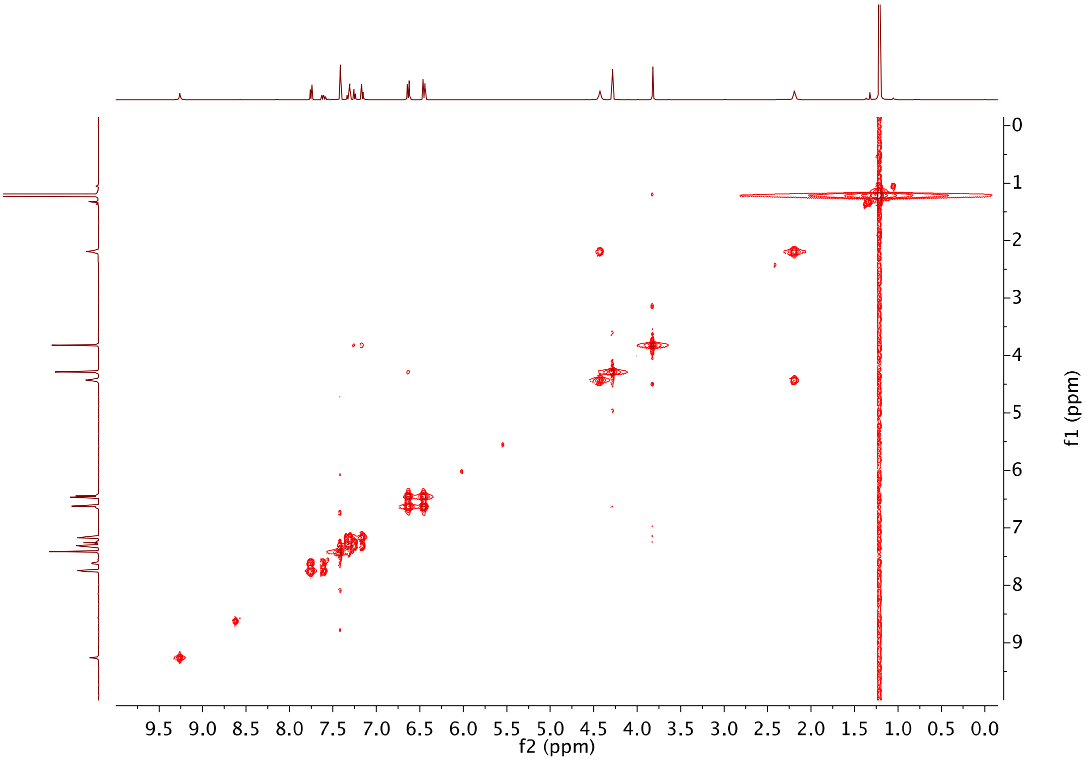

Figure S8. COSY NMR ($CDCl_3$) of **3**

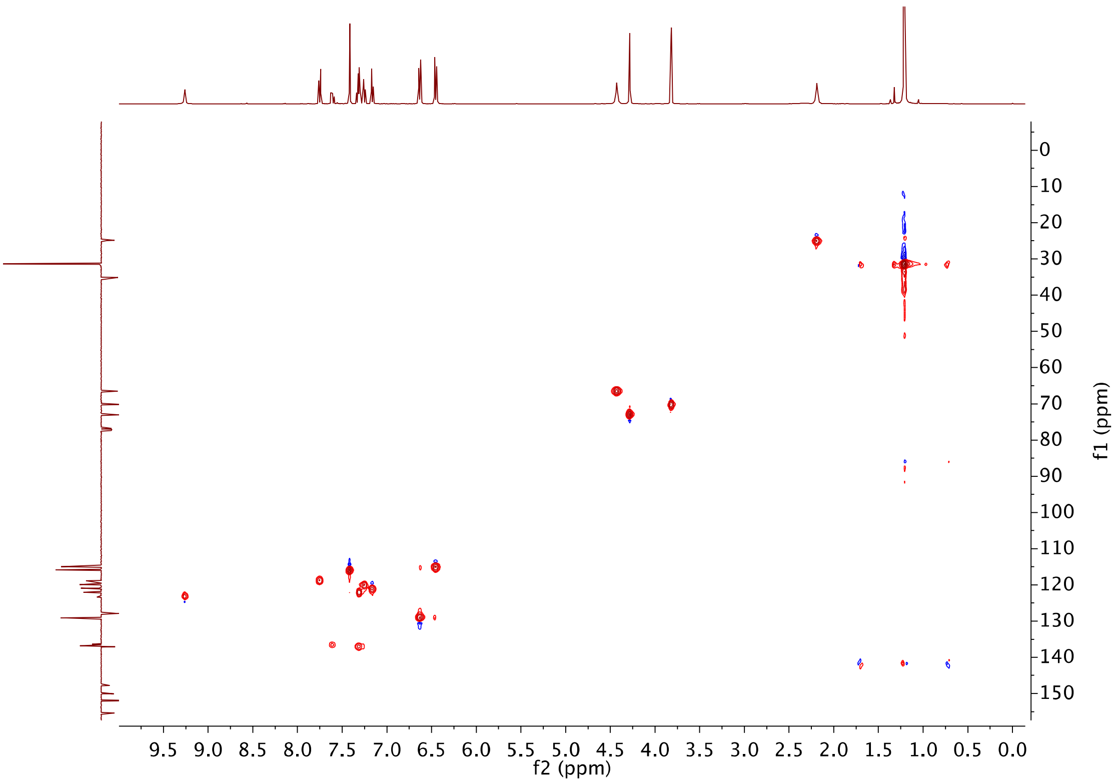

Figure S9. HSQC NMR ($CDCl_3$) of **3**

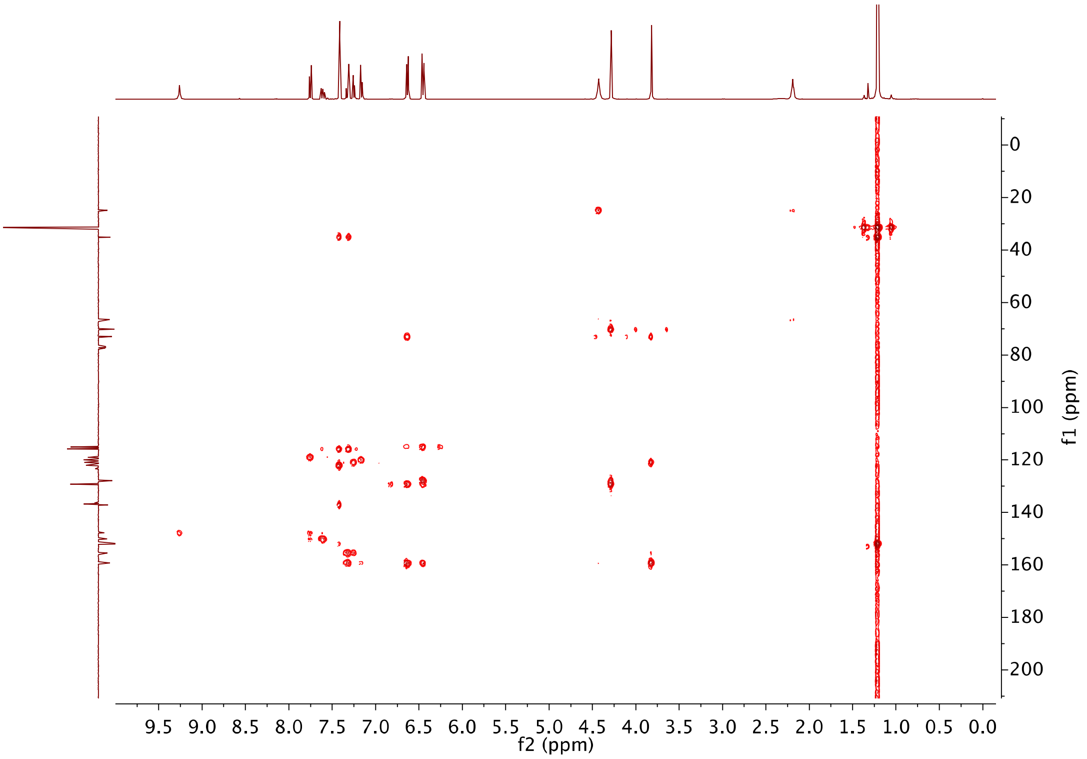

Figure S10. HMBC NMR ($CDCl_3$) of **3**

1.4 General procedure for the preparation of Co^{II} complexes

The rotaxane ligand (0.009 mmol) was dissolved in MeCN (4.1 mL) and a pale pink solution of $Co(ClO_4)_2.6H_2O$ (0.9 mL, 10 mM in MeCN) [NOTE: ClO_4 salts are potentially explosive and should be handled with care on small scales] was added. The pale-yellow solution was stirred at 80 °C for 1 h. The solvent was removed *in vacuo*, the orange-brown residue was redissolved in MeCN (~1 mL) and the product was precipitated by vapour diffusion with Et_2O to give the desired metal complex as an microcrystalline solid.

1.5 Synthesis of complex [Co(**2**)]$(ClO_4)_2$

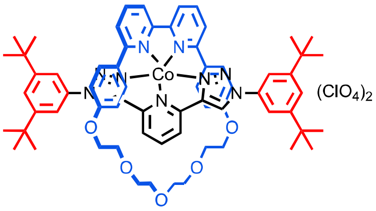

ESI-MS m/z = 573.3 [M – 2(ClO_4)]$^{2+}$ (calc. 573.3 for [$C_{67}H_{77}CoN_9O_5$]$^{2+}$).

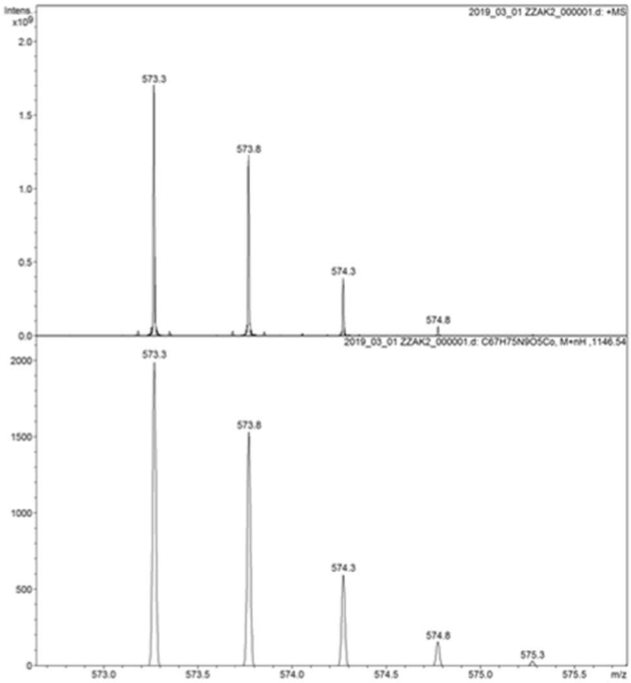

Figure S11. Comparison of the isotope pattern obtained by ESI-MS for [Co(**2**)]$(ClO_4)_2$ and a calculated model based on the molecular formula

1.6 Synthesis of complex [Co(**3**)](ClO_4)$_2$

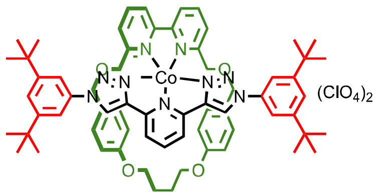

ESI-MS m/z = 565.3 [M – 2(ClO_4)]$^{2+}$ (calc. 565.3 for [$C_{67}H_{77}CoN_9O_4$]$^{2+}$).

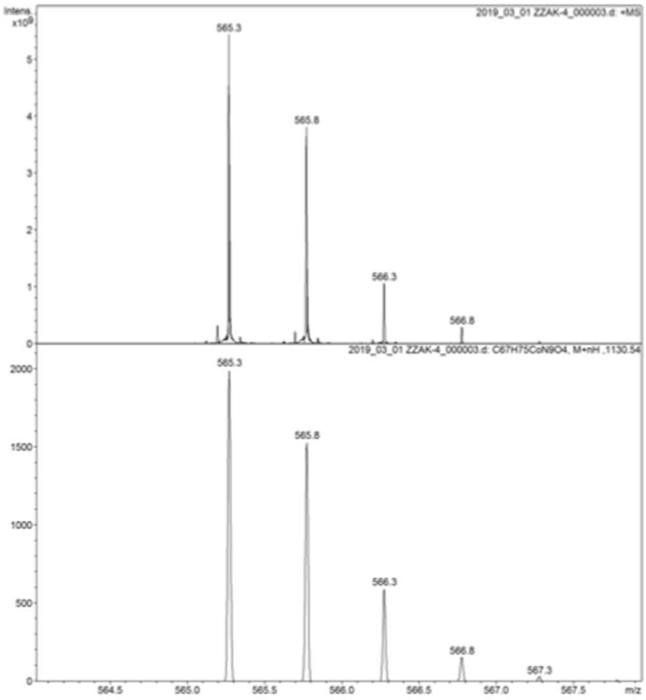

Figure S12. Comparison of the isotope pattern obtained by ESI-MS for [Co(**3**)](ClO_4)$_2$ and a calculated model based on the molecular formula

2 Single-crystal X-ray Diffraction

2.1 X-ray crystallographic data for [Co(**1**)]$(ClO_4)_2$

The solid state structure of [Co(**1**)](ClO4)$_2$ was reported in a previous publication and deposited in the CCDC (1863245). The data and related discussion are reproduced here for completeness.[7] The orange crystals were grown by vapour diffusion of Et_2O into a solution of the complex in MeCN with 5-10% CH_2Cl_2. There are two crystallographically independent $[Co(\mathbf{1})]^{2+}$ moieties in the asymmetric unit. Although superficially similar, the N-Co bond lengths differ between the two, with average N-Co bond lengths of 1.98 and 2.07 Å, suggesting the former is LS Co^{II} whilst the latter is HS Co^{II}. These values are consistent with bond lengths reported for LS and HS Co^{II} complexes with polypyridyl ligands.[8]

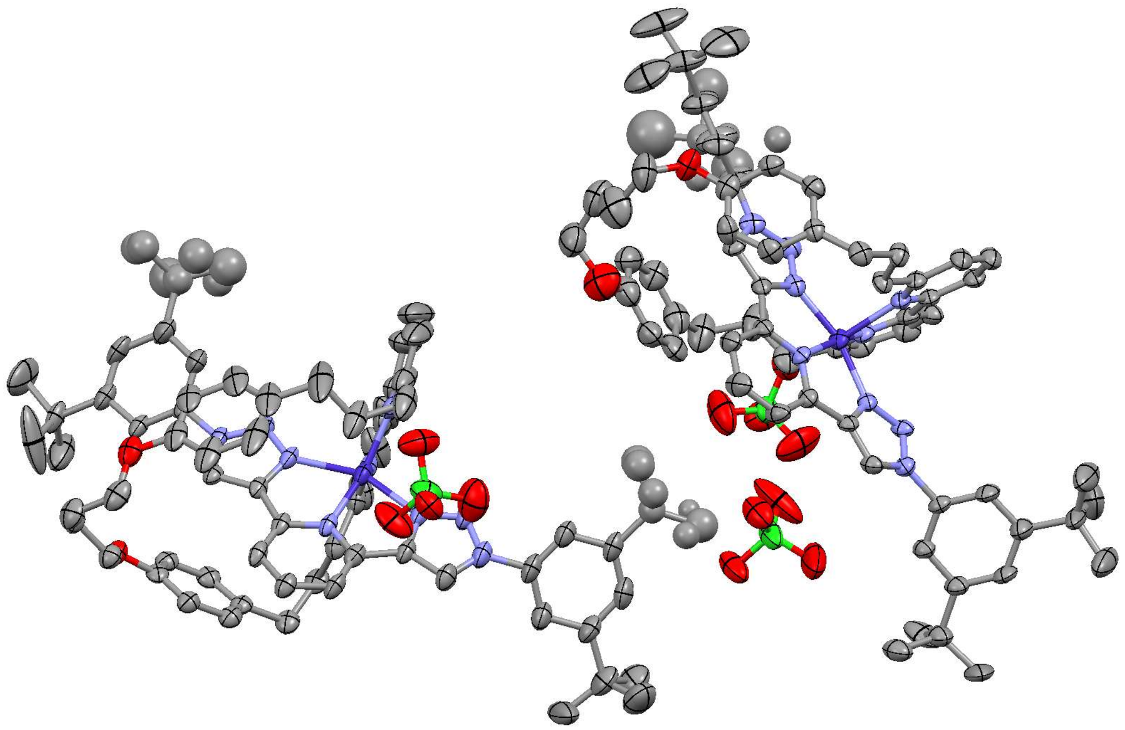

Figure S13. Ellipsoid plot of the asymmetric unit of [Co(**1**)]$(ClO_4)_2$. Ellipsoids are shown at the 50% probability level. Hydrogen atoms have been omitted for clarity. One of the four anions in the asymmetric unit was highly disordered and could not be located.

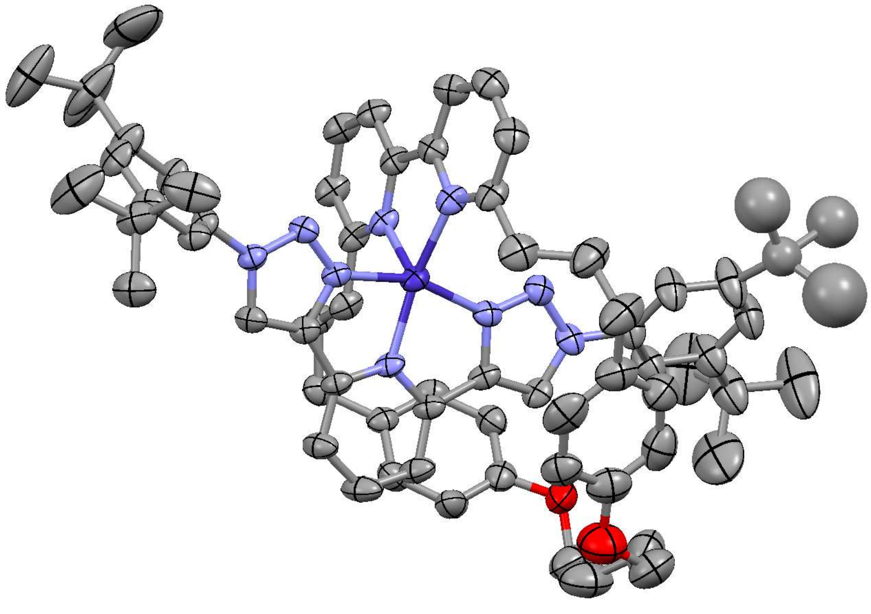

Figure S14. Ellipsoid plot of one of the crystallographically independent $[Co(\mathbf{1})]^{2+}$ units. Ellipsoids are shown at the 50% probability level. Hydrogen atoms and anions have been omitted for clarity.

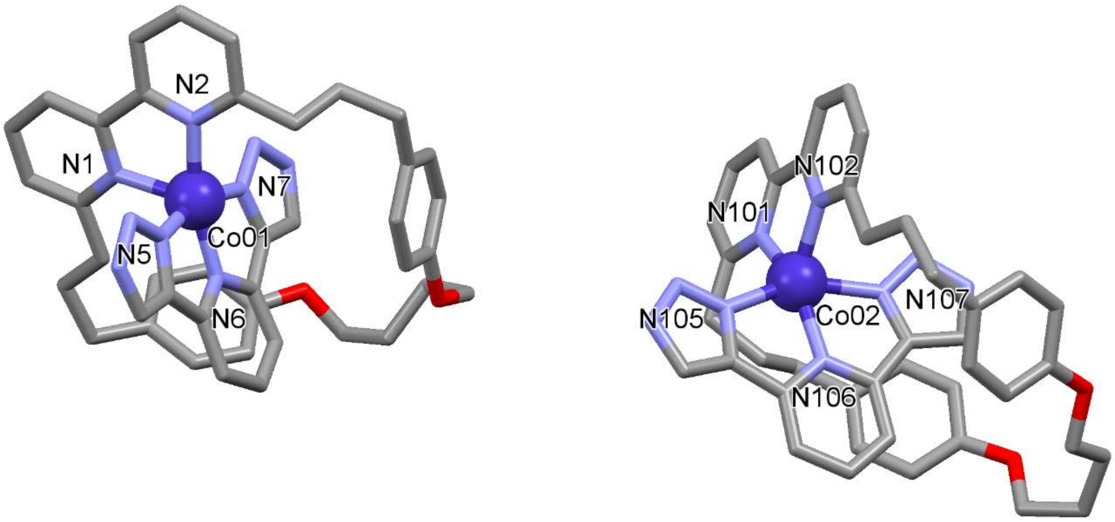

Figure S15. Stick plot of the two Co^{II} coordination environments in the asymmetric unit of $[Co(\mathbf{1})](ClO_4)_2$. Counterions, hydrogen atoms and stoppering groups have been omitted for clarity. Bond lengths (Å): N1-Co01 2.098(3); N2-Co01 1.958(3); N5-Co01 1.975(3); N6-Co01 1.926(3); N7-Co01 1.961(4); N101-Co02 2.047(3); N102-Co02 2.032(4); N105-Co02 2.071(4); N106-Co02 2.073(3); N107-Co02 2.135(4).

**Compound**	[Co(**1**)](ClO_4)$_2$
CCDC	1863245
Empirical formula	$C_{69}H_{80.5}Cl_{1.5}CoN_9O_8$
Formula weight	1276.02
Temperature/K	100.15
Crystal system	triclinic
Space group	P-1
a/Å	17.9110(4)
b/Å	19.5002(4)
c/Å	21.7320(5)
α/°	79.191(2)
β/°	78.964(2)
γ/°	85.424(2)
Volume/Å^3	7310.2(3)
Z	4
ρ_{calc}g/cm^3	1.159
μ/mm^1	0.345
F(000)	2696.0
Crystal size/mm^3	0.17 × 0.13 × 0.13
Radiation	Mo Kα (λ = 0.71073)
2Θ range for data collection/°	5.8 to 57.86
Index ranges	-24 ≤ h ≤ 23, -22 ≤ k ≤ 26, -28 ≤ l ≤ 24
Reflections collected	91166
Independent reflections	25700 [R_{int} = 0.0480,R_{sigma} = 0.1041]
Data/restraints/parameters	25700/5/1601
Goodness-of-fit on F^2	1.058
Final R indexes [I>=2σ (I)]	R_1 = 0.0806, wR_2 = 0.2039
Final R indexes [all data]	R_1 = 0.1172, wR_2 = 0.2321
Largest diff. peak/hole / e Å^{-3}	0.96/-0.71

2.2 Single crystal X-ray crystallographic data for [Co(**2**)](ClO_4)$_2$

Crystals of complex [Co(**2**)](ClO_4)$_2$ suitable for SCXRD were grown by vapour diffusion of Et_2O into a solution of the complex in 1 : 1 MeCN-CH_2Cl_2 to give single brown block crystals. A suitable crystal with dimensions 0.19 × 0.10 × 0.07 mm was selected and mounted on a Rigaku 007HF equipped with Varimax confocal mirrors and an AFC11 goniometer and HyPix 6000 detector diffractometer. The crystal was kept at a steady T = 100(2) K during data collection. The structure was solved with the ShelXT solution program[9] using dual methods and by using Olex2 1.3[10] as the graphical interface. The model was refined with ShelXL 2018/3[9] using full matrix least squares minimisation on F2. All non-hydrogen atoms were refined anisotropically. Hydrogen atom positions were calculated geometrically and refined using the riding model. Two of the four anions in the asymmetric unit were highly disordered and could not be located. Solvent masking has been applied to eliminate the electronic contribution of these diffuse counterions and solvent (2 × ClO_4^- per asymmetric unit and Et_2O and MeCN in unknown proportions). All disordered parts of the structure have 1,2 and 1,3 equal distance geometric restraints applied to equivalent pairs of atoms in each disorder component. Thermal restraints were applied to disordered ^{t}Bu groups.

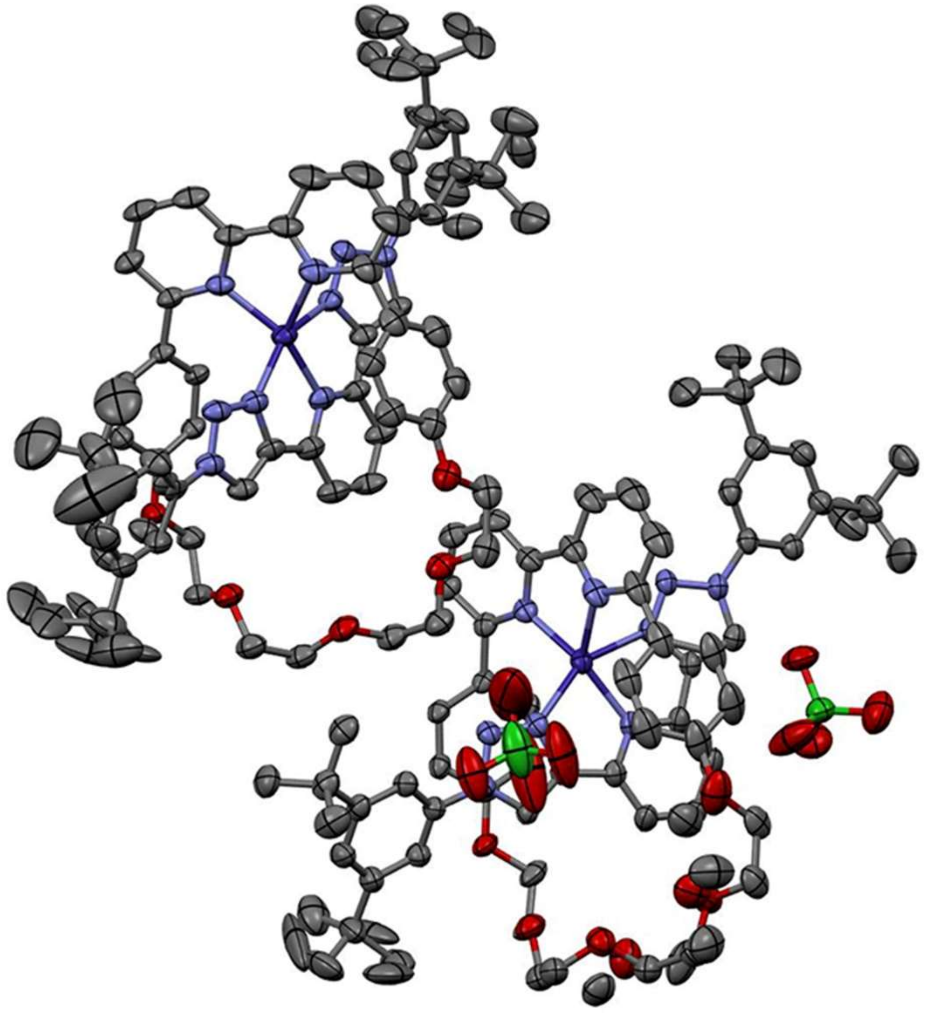

Figure S16. Ellipsoid plot of the asymmetric unit of [Co(**2**)](ClO_4)$_2$. Ellipsoids are shown at the 50% probability level. Hydrogen atoms have been omitted for clarity. Two of the four anions in the asymmetric unit were highly disordered and could not be located.

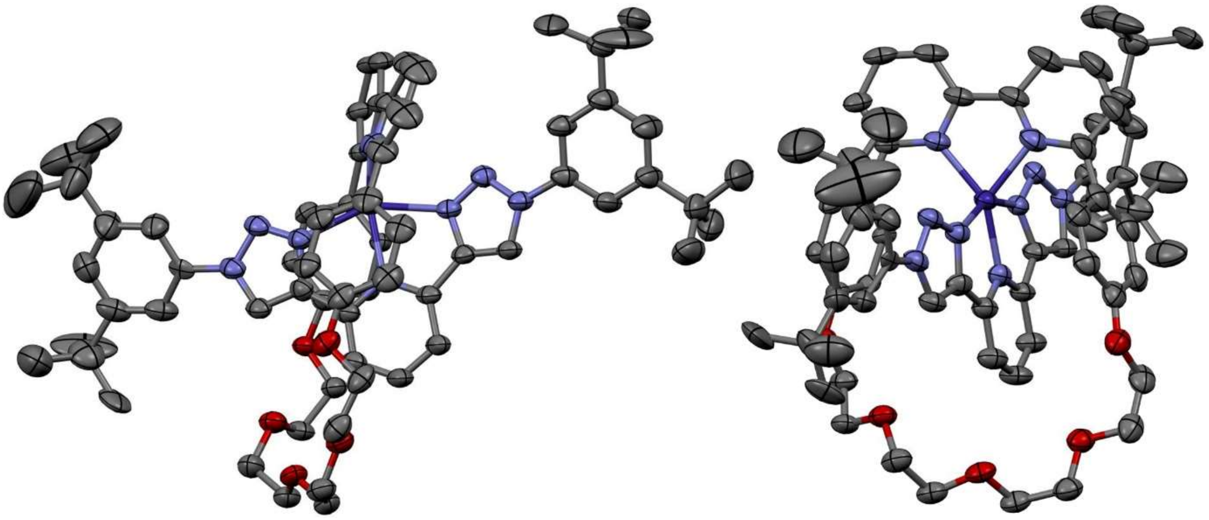

Figure S17. Ellipsoid plot of one of the crystallographically independent $[Co(\mathbf{2})]^{2+}$ units viewed from the side (left) and front (right). Ellipsoids are shown at the 50% probability level. Hydrogen atoms and anions have been omitted for clarity.

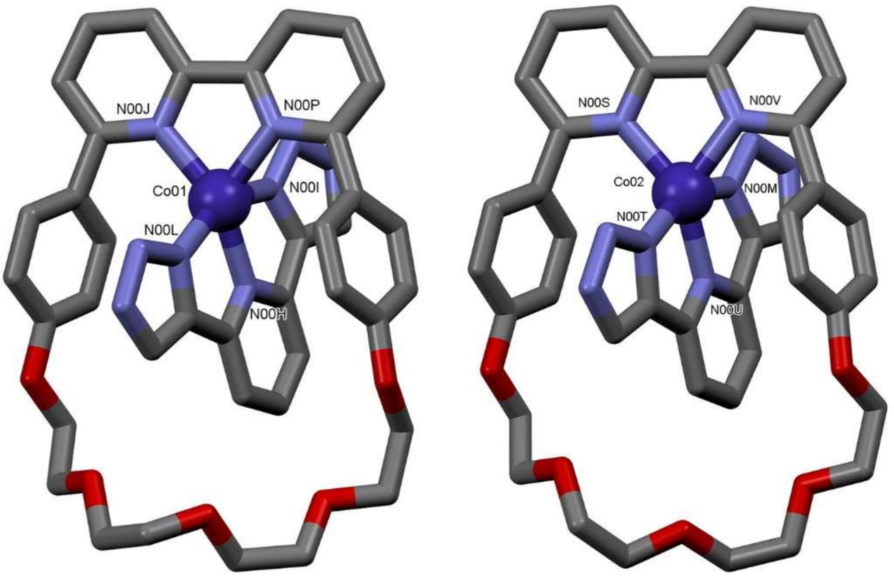

Figure S18. Stick plot of the two Co^{II} coordination environments in the asymmetric unit of $[Co(\mathbf{2})](ClO_4)_2$. Counterions, hydrogen atoms and stoppering groups have been omitted for clarity. Bond lengths (Å): Co01-N00J 2.071; Co01-N00H 2.071; Co01-N00P 2.072; Co01-N00L 2.137; Co01-N00I 2.198; Co02-N00V 2.060; Co02-N00S 2.067; Co02-N00U 2.077; Co02-N00T 2.135; Co02-N00M 2.199.

**Compound**	[Co(**2**)](ClO_4)$_2$
Formula	$C_{67}H_{77}ClCoN_9O_9$ + [ClO_4 + solvent]
$D_{calc.}$/ g cm^{-3}	1.039
μ/mm^{-1}	2.409
Formula Weight	1246.75
Colour	brown
Shape	block
Size/mm^3	0.19×0.10×0.07
T/K	100(2)
Crystal System	triclinic
Space Group	P-1
a/Å	16.0634(2)
b/Å	21.8864(4)
c/Å	25.3399(4)
α/°	111.5424(16)
β/°	101.7314(14)
γ/°	95.4570(14)
V/Å^3	7971.1(2)
Z	4
Z'	2
Wavelength/Å	1.54178
Radiation type	Cu K$_\alpha$
Θ_{min}/°	3.014
Θ_{max}/°	70.646
Measured Refl's.	134397
Indep't Refl's	29546
Refl's I≥2 σ(I)	22278
R_{int}	0.0783
Parameters	1869
Restraints	1893
Largest Peak	1.073
Deepest Hole	-0.773
GooF	1.040
wR_2 (all data)	0.2628
wR_2	0.2443
R_1 (all data)	0.1066
R_1	0.0872

2.3 X-ray crystallographic data for [Co(**3**)](ClO_4)$_2$

Crystals of complex [Co(**3**)](ClO_4)$_2$ suitable for SCXRD were grown by suspending a droplet of a DMF solution of the complex above a bath of 1 : 1 H_2O-MeOH to give single yellow block crystals of [Co(**3**)](ClO_4)$_2$. A suitable crystal with dimensions 0.07 × 0.04 × 0.03 mm was selected and mounted on a Rigaku 007HF equipped with Varimax confocal mirrors and an AFC11 goniometer and HyPix 6000 detector diffractometer. The crystal was kept at a steady T = 100(2) K during data collection. The structure was solved with the ShelXT solution program[9] using dual methods and by using Olex2 1.3[10] as the graphical interface. The model was refined with ShelXL 2018/3[9] using full matrix least squares minimisation on F2. All non-hydrogen atoms were refined anisotropically. Hydrogen atom positions were calculated geometrically and refined using the riding model. All disordered parts of the structure have 1,2 and 1,3 equal distance geometric restraints applied to equivalent pairs of atoms in each disorder component. Thermal restraints were applied to disordered atoms of the rotaxane ring. The diffraction pattern indicates commensurate modulation along the b-axis with a q-vector of 0 0.5 0. This has been modelled using the supercell approach with a doubled b-axis giving rise to a Z' = 2 structure.

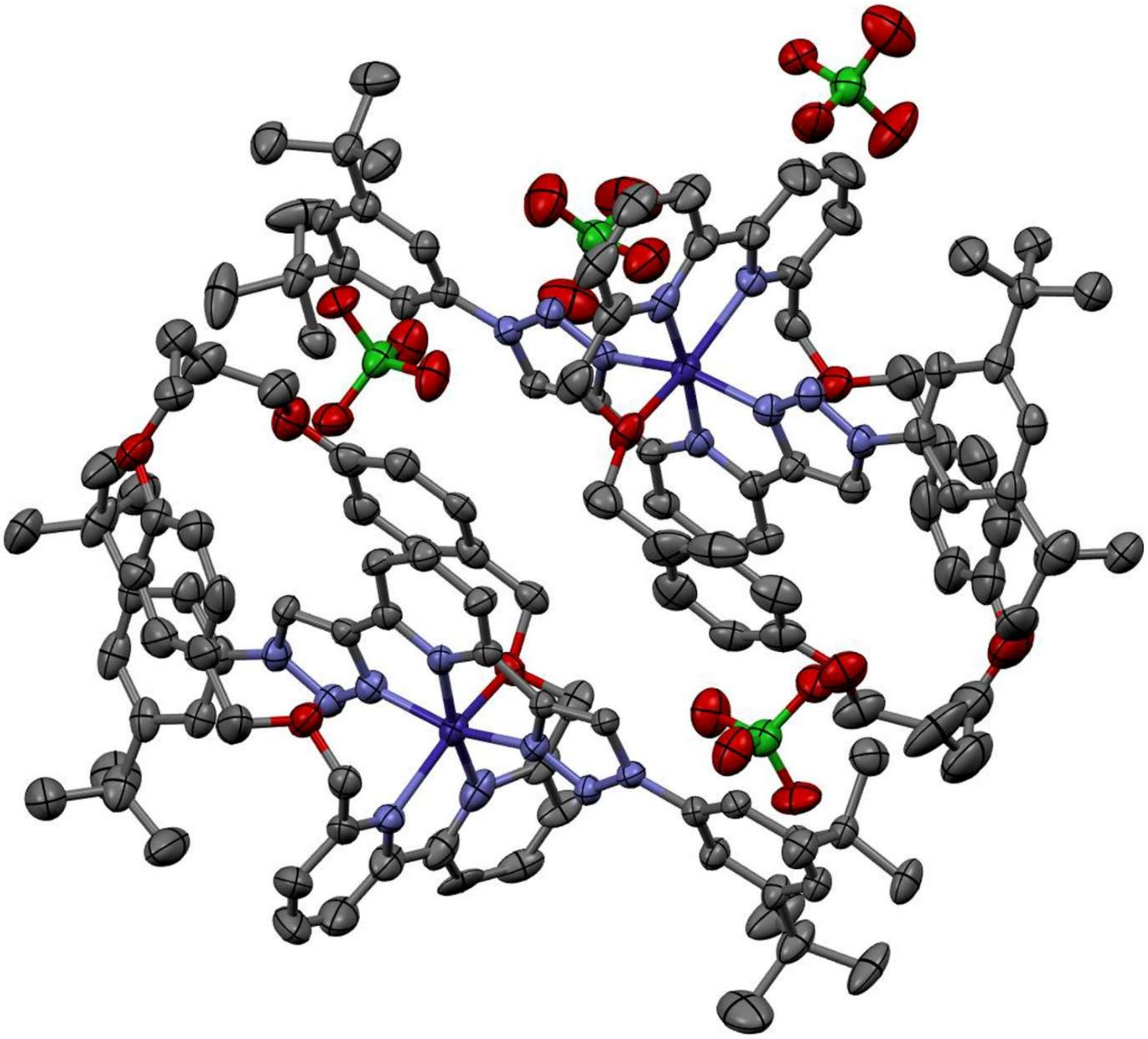

Figure S19. Ellipsoid plot of the asymmetric unit of [Co(**3**)](ClO_4)$_2$. Ellipsoids are shown at the 50% probability level. Hydrogen atoms have been omitted for clarity.

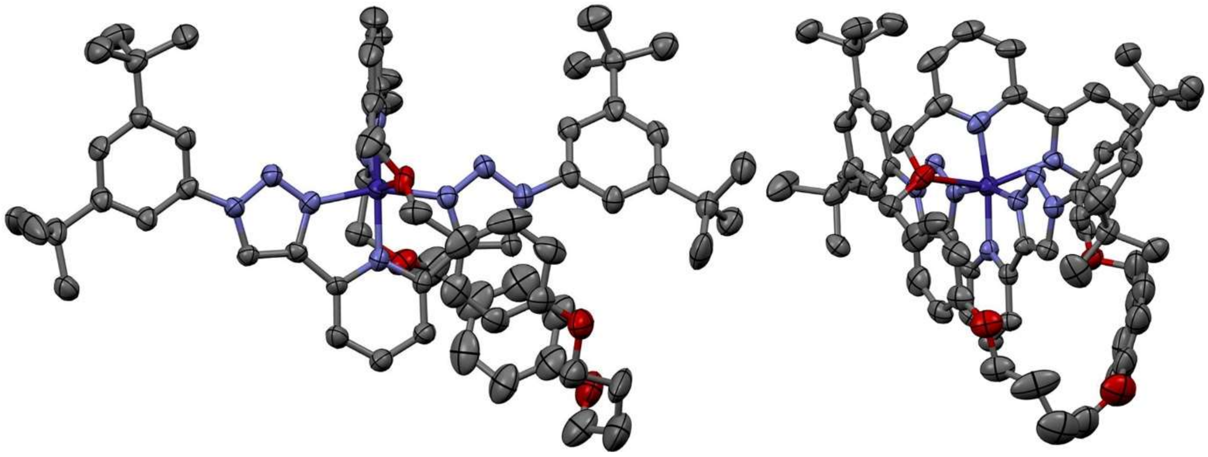

Figure S20. Ellipsoid plot of one of the crystallographically independent [Co(**3**)]$^{2+}$ units viewed from the side (left) and front (right). Ellipsoids are shown at the 50% probability level. Hydrogen atoms and anions have been omitted for clarity.

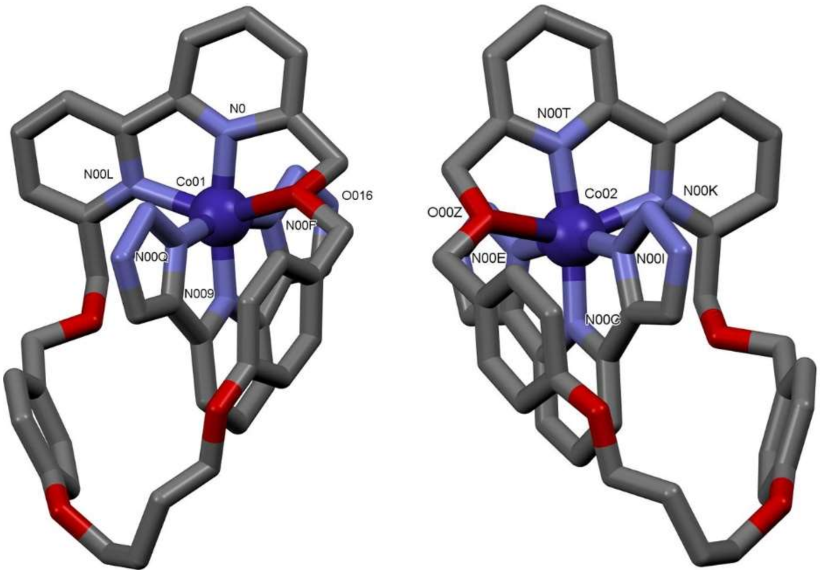

Figure S21. Stick plot of the two Co^{II} coordination environments in the asymmetric unit of [Co(**3**)](ClO_4)$_2$. Counterions, hydrogen atoms and stoppering groups have been omitted for clarity. Bond lengths (Å): Co01-N009 1.898; Co01-N0 1.912; Co01-N00F 1.922; Co01-N00Q 1.926; Co01-N00L 2.166; Co01-O016 2.378; Co02-N00C 1.912; Co02-N00T 1.920; Co02-N00E 1.922; Co02-N00I 1.931; Co02-N00K 2.207; Co02-O00Z 2.412.

**Compound**	[Co(**3**)]$(ClO_4)_2$
Formula	$C_{67}H_{77}Cl_2CoN_9O_{12}$
$D_{calc.}$/ g cm^{-3}	1.364
μ/mm^{-1}	3.407
Formula Weight	1330.20
Colour	yellow
Shape	block
Size/mm^3	0.07×0.04×0.03
T/K	100(2)
Crystal System	triclinic
Space Group	P-1
a/Å	16.2078(7)
b/Å	20.0541(7)
c/Å	21.1503(9)
α/°	88.396(3)
β/°	71.007(4)
γ/°	85.105(3)
V/Å^3	6476.6(5)
Z	4
Z'	2
Wavelength/Å	1.54178
Radiation type	Cu K_α
Θ_{min}/°	2.209
Θ_{max}/°	68.249
Measured Refl's.	116933
Indep't Refl's	12543
Refl's I≥2σ(I)	23549
R_{int}	0.1561
Parameters	1844
Restraints	233
Largest Peak	0.874
Deepest Hole	-1.057
GooF	1.004
wR_2 (all data)	0.2845
wR_2	0.2258
R_1 (all data)	0.1490
R_1	0.0878

2.4 Comparison between independent structures in the asymmetric units of [Co(**1-3**)](ClO_4)$_2$

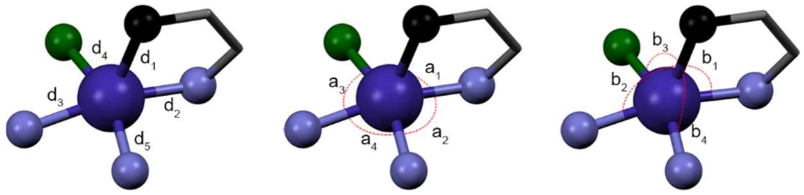

Figure S22. Bond distances and angles used when comparing structures. The link between the bipyridine Ns in included to improve clarity. The apical N of the square-based pyramid is shown in black. The triazole N with the shorter N-Co distance is shown in green.

The unit cells of the three experimental crystal structures contain two independent molecules. In the case of [Co(**2**)]$^{2+}$ and [Co(**3**)]$^{2+}$, both structures are very similar and can be assigned to HS spin configurations by comparison with modelled structures. The standard deviations between the bond angles and distances of the two independent structures in the asymmetric unit for [Co(**1-3**)]$^{2+}$ are given in Tables S1 and S2 respectively. For [Co(**1**)]$^{2+}$, each molecule can be assigned to a different electronic configuration. These observations are broadly in line with the calculated LS-HS gaps ([Co(**1**)]$^{2+}$: 2.7 kJ/mol, [Co(**2**)]$^{2+}$: 46 kJ/mol, [Co(**3**)]$^{2+}$: 11.8 kJ/mol).

Table S1. Bond angles (°) of the independent molecules in the asymmetric units of [Co(**1-3**)]$^{2+}$

	**a_1**	**a_2**	**a_3**	**a_4**	**b_1**	**b_2**	**b_3**	**b_4**	**RMSD**
[Co(**1**)]$^{2+}$	98.50	98.66	80.12	80.56	81.07	115.71	96.89	95.56	7.9
	102.50	100.67	77.92	74.93	81.11	130.33	109.63	87.75	
[Co(**2**)]$^{2+}$	104.68	96.74	77.52	74.64	78.46	128.56	121.53	80.21	1.7
	102.25	99.32	77.42	74.67	78.57	131.05	120.25	81.48	
[Co(**3**)]$^{2+}$	97.22	100.13	80.59	81.34	80.12	108.9	101.92	87.01	0.5
	97.31	100.2	80.79	81.11	79.54	107.65	101.78	87.24	

Table S2. Co-N bond lengths (Å) of the independent molecules in the asymmetric units of [Co(**1-3**)]$^{2+}$

**Molecules**	**d_1**	**d_2**	**d_3**	**d_4**	**d_5**	**RMSD**
[Co(**1**)]$^{2+}$	2.098	1.958	1.926	1.961	1.975	0.120
	2.032	2.047	2.073	2.071	2.135	
[Co(**2**)]$^{2+}$	2.073	2.071	2.071	2.137	2.198	0.007
	2.06	2.067	2.077	2.135	2.199	
[Co(**3**)]$^{2+}$	2.166	1.912	1.898	1.922	1.926 (2.378)[a]	0.002
	2.207	1.92	1.912	1.922	1.931 (2.412)[a]	

[a] Distance O-Co for [Co(**3**)]$^{2+}$ complex.

2.5 Geometric parameters for [Co(**1**-**3**)](ClO_4)$_2$

τ_5 values[11] for the crystallographically independent structures of both [Co(**1**)]$^{2+}$ (τ_5 = 0.05 for LS, 0.10 for HS) and [Co(**2**)]$^{2+}$ (τ_5 = 0.07, 0.09) indicate the Co(II) ions are best described as possessing pseudo-square-based bipyramidal geometries. For the hexacoordinate [Co(**3**)]$^{2+}$ species common octahedral distortion parameters were calculated using the OctaDist programme[12] (ζ = 0.95 Å, 1.03 Å; Σ = 128°, 115°; Θ = 420°, 377°) and found to be commensurate with values observed for HS Co(II)N_6 centres in SCO-active mononuclear complexes assembled from *fac*-tridentate ligands.[13]

3 EPR spectroscopy

3.1 EPR sample preparation

Frozen solution EPR samples were prepared aerobically by transferring ~ 10 µl of a 10 mM solution of the complex in 1,2-dichloroethane to a 1.6 mm (O.D.) EPR tube (Goss Scientific) using a 100 µl Hamilton syringe (SGE Analytical Service) and flash-freezing the tube in liquid nitrogen. Powder EPR samples were prepared transferring finely ground powder of the complexes into the EPR tubes to reach a height of 6 mm of powder in the tube.

3.2 EPR measurements

X-band EPR. EPR spectra were scaled by power, receiver gain, number of scans and frequency. All X-band EPR measurements were performed using an X/Q-band Bruker Elexsys E580 Spectrometer (Bruker BioSpin GmbH, Germany) equipped with a close-cycle cryostat (Cryogenic Ltd, UK). The EPR measurement temperature was calibrated with an external Cernox thermometer (LakeShore cryotronics). All X-band measurements were carried out in an X-band split ring resonator module with 2 mm sample access (Bruker, ER 4118X-MS-2W) operated in continuous wave (CW) mode. Unless otherwise stated, measurement conditions for CW measurements were as follows: 2.016 mW microwave power, 0.7 mT modulation amplitude, 10-minute acquisition time. The acquisition temperature is specified in Figure legends.

High-frequency EPR measurements. High-frequency EPR (HFEPR) spectra at 4.5 K and frequencies between 95 GHz and 375 GHz were recorded on pelletized samples of **1**, **2** and **3** on a home-built spectrometer at Stuttgart University, featuring an Anritsu signal generator, a VDI amplifier-multiplier chain, a Thomas Keating quasi-optical bridge, an Oxford Instruments 15/17 T solenoid cryomagnet and a QMC Instruments InSb hot electron bolometer.

3.3 EPR simulations

Low-spin X-band EPR spectra were simulated with Easyspin[14] using the following spin Hamiltonian:

$$H = \hat{S} \cdot \boldsymbol{D} \cdot \hat{S} + \beta \hat{B} \cdot \boldsymbol{g} \cdot \hat{S} + \hat{S} \cdot \boldsymbol{A} \cdot \hat{I}, \qquad \text{eq. 1}$$

where the terms are ordered in magnitude of energy: zero-field splitting interaction, electron Zeeman interaction, hyperfine coupling. The quadrupole and nuclear Zeeman interactions are not explicitly considered since they their contribution is negligible with respect to the other terms.

High spin signals at X-band and HFEPR spectra were simulated with PHI[15] in order to use the same software for both the EPR and the magnetic data. Moreover, reasonable intensities of the high-spin signals could be achieved only in simulations performed with PHI; however, low-spin signals in which the hyperfine splitting is visible could not be reproduced with this software due to the impossibility of including hyperfine interactions. For this reason, the zero-field splitting and the Zeeman interactions were solely considered in simulations carried out with PHI. Despite being relevant to the case – as it gives rise to the ZFS interactions – the SOC is neither considered in the fitting of the magnetic data nor in the simulation of the EPR spectra; its contribution is thought to be of the second order, making its inclusion as an explicit term unnecessary. Even though results and parameters extracted from them are presented here in a sequential progression that goes from X-band EPR to HFEPR to SQUID measurements, the final fits and simulations were obtained considering all data simultaneously.

Scheme S1 shows the approach used in the evaluation of the magnetic parameters:

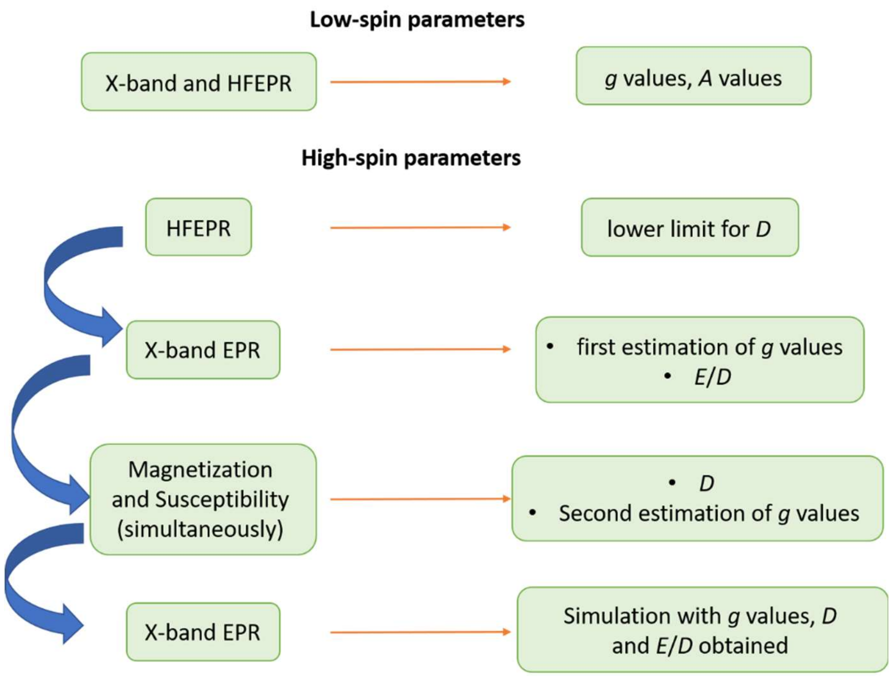

Scheme S1. Diagram of the approach followed to obtain the magnetic parameters for [Co(**1-3**)](ClO_4)$_2$ from X-band, HFEPR and SQUID experiments.

3.4 Frozen-solution X-band EPR spectra

Solution state X-band experiments were carried out on 10 mM solutions of [Co(**1-3**)](ClO_4)$_2$ in dichloroethane at 10 and 100 K (Figure S23). The most striking difference with respect to the powder samples (Figure 2A & B in the main paper) is the absence of the transition at high fields for both **1** and **3**, and the appearance of another resonant line (Figure S2, left) for **2**, whose nature is still unclear. The splitting on the low field transition observed in the solution spectra allowed the determination of the hyperfine coupling of the high-spin state of **2**, which was found to be 375 MHz. In order to reproduce this splitting, Easyspin[14] was used to simulate the high-spin solution spectra; both spin configurations were taken into account, weighted arbitrarily so that the experimental signal intensities matched the experimental lines, and the explicit addition of the temperature as one of the experimental parameters resulted in a reasonably good simulation of the saturated low-spin signal at 10 K. Overall, the high similarity of these experiments to the spectra obtained for powdered samples shows the shielding effect provided by the rotaxane structure, since no coupling of the complexes occurs in the solid state due to the bulkiness of the ligands.

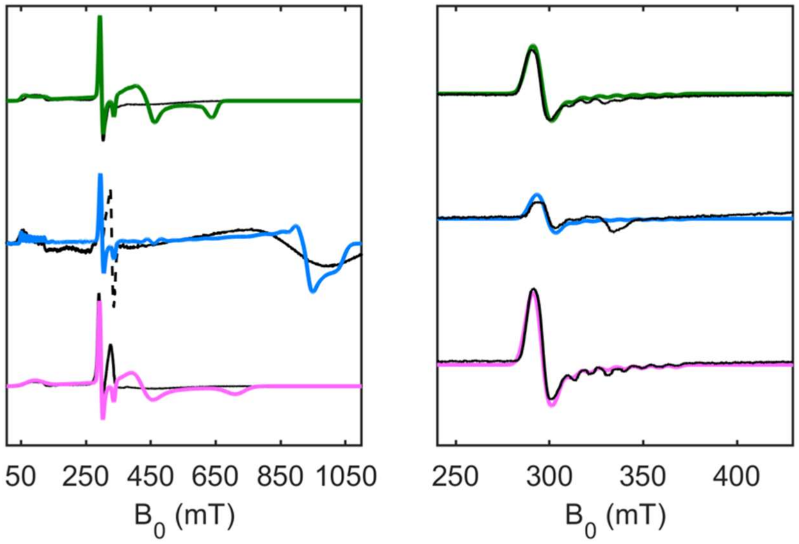

Figure S23. Frozen-solution X-band EPR spectra (in black) at 10 K (left) and at 100 K (right) and simulations in green, blue and magenta for [Co(**1-3**)](ClO_4)$_2$, respectively.

3.5 Low-spin state EPR simulation parameters

Table S3. EPR parameters of the low-spin fraction in [Co(**1-3**)](ClO_4)$_2$ obtained from simulations of X-band spectra of powder samples.

	g_1	g_2	g_3	A_1	A_2	A_3	H_1	H_2	H_3
[Co(**1**)](ClO_4)$_2$	2.292	2.292	2.013	30	30	260	120	120	50
[Co(**2**)](ClO_4)$_2$	2.274	2.274	2.012	30	30	260	120	120	50
[Co(**3**)](ClO_4)$_2$	2.272	2.215	2.015	30	30	260	120	120	50

3.6 Determination of negative zero-field splitting from high-spin EPR signals

The effective g value of low field resonance of the high-spin signals in Figure 2A in the main paper suggests a negative zero-field splitting term and a M_s = ± 3/2 ground state. This assignment is supported by the g^{eff}-g^{true} formulae[16]:

$$g_x^{eff} = g_x\left(1 \pm \frac{1-3\lambda}{\sqrt{1+3\lambda^2}}\right) \quad \text{eq. 2}$$

$$g_y^{eff} = g_y\left(1 \pm \frac{1+3\lambda}{\sqrt{1+3\lambda^2}}\right) \quad \text{eq. 3}$$

$$g_z^{eff} = g_z\left(1 \mp \frac{2}{\sqrt{1+3\lambda^2}}\right) \quad \text{eq. 4}$$

where the upper sign refers to M_s = ± 1/2 (and $D > 0$), the lower sign refers to M_s = ± 3/2 (and $D < 0$) and $\lambda = |E/D|$. These equations show that signals having effective g = 7.9 are only possible for transitions occurring within the M_s = ± 3/2 manifold and characterized by true g_z values > 2.5.

Whereas an accurate estimation of D cannot be obtained by X-band measurements, the presence of more than one transition – in contrast to HFEPR spectra (see Section 3.7 below) - allowed the precise evaluation of the rhombicity of both the g and the D tensors for all rotaxanes (Table 1, main paper). The presence of two transitions at high fields observed for **1** and **3** is then explained in terms of a larger value of $|E/D|$ for **1** (0.21) and **3** (0.22) compared to **2** (0.11), where D values of –78, –59 and – 90 cm^{-1} (obtained from fitting the magnetization and susceptibility data) were used for **1-3**, respectively.

3.7 HFEPR measurements

Satisfactory simulations of X-band (Figure 2C in the main paper) and HFEPR spectra (Figure S24) were obtained using S = 3/2 and by fixing an arbitrarily large D. The linear frequency dependence of the low-field transition (Figure S24D) shows that the zero-field splitting ($|2D|$) is much larger than the Zeeman splitting and the intercept at zero confirms the intra-doublet nature of the g_{eff} transition. The lack of the second transition visible in the simulations at 95 and 125 GHz, may be due to the field-induced alignment phenomenon,[17] which occurs when microcrystallites align to the external magnetic field and that has been previously observed for other high-spin cobalt complexes.[18,19]

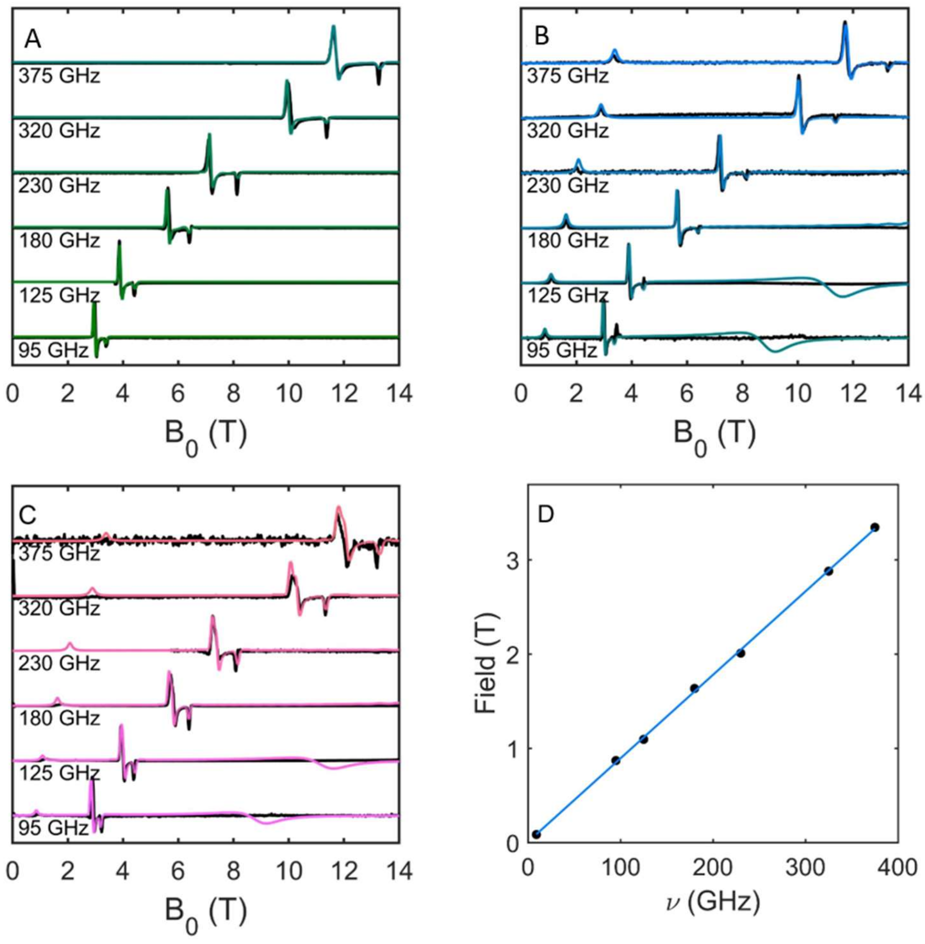

Figure S24. HFEPR spectra and simulations of [Co(**1**)]$(ClO_4)_2$ (A, green), [Co(**2**)]$(ClO_4)_2$ (B, blue) and [Co(**3**)]$(ClO_4)_2$ (C, magenta) acquired at 4.5 K at variable frequency. D shows the field position of the g_{eff} = 7.9 of **2** as a function of the measurement frequency. The intercept of the linear regression at 0 implies that there is no zero-field splitting term at zero frequency, which is only true for intra-doublet transitions and hence allows assignment of the g_{eff} = 7.9 peak to an intra-doublet transition.

4 Magnetization

4.1 Sample preparation and magnetic measurements

The magnetic susceptibility measurements were performed using a Quantum Design MPMS-XL SQUID magnetometer equipped with a 7 T magnet. Powder pellet samples were slightly pressed and wrapped with Teflon (mass = 20.1 mg, 23.9 mg and 21.0 mg for [Co(**1-3**)]$(ClO_4)_2$, respectively). Temperature-dependent susceptibility experiments were carried out under a static magnetic field of 0.1 T and in the temperature range 2-300 K. Field dependent/reduced magnetization measurements were performed in a field range of 0-7 T and over the temperature range 1.8-16 K. Alternating current (ac) susceptibility measurements were carried out under an oscillating ac field of 1.55 Oe and ac frequencies ranging from 1 to 1500 Hz, at an applied magnetic field of 2500 Oe (determined to be the optimal field for magnetic relaxation).

4.2 Field-dependent magnetization measurements for [Co(**1-3**)]$(ClO_4)_2$

The decrease of the susceptibility observed at low temperatures (manuscript Figure 3) was attributed to the presence of significant magnetic anisotropy rather than (strongly-coupled) antiferromagnetic impurities or interactions between the spins, as the field dependent magnetization data collected at 100 K show a perfectly linear trend for all compounds (**Figure S25**).

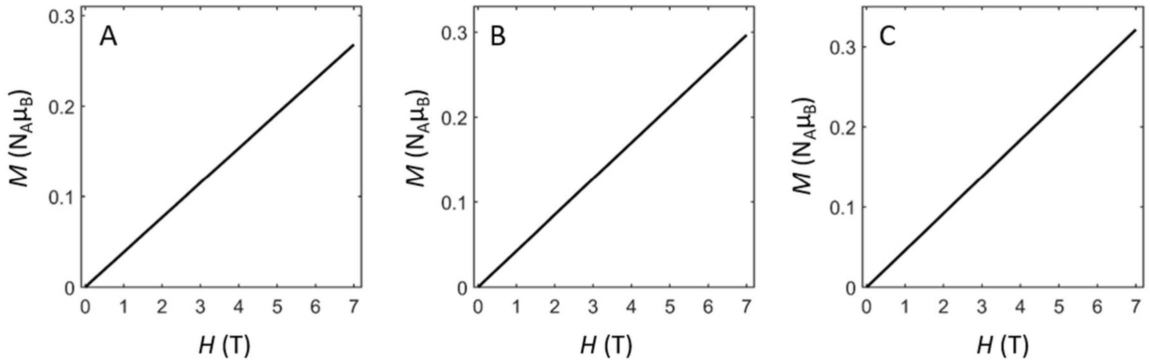

Figure S25. Magnetization (*M*) vs field (*H*) at 100 K for [Co(**1**)]$(ClO_4)_2$ (A), [Co(**2**)]$(ClO_4)_2$ (B) and [Co(**3**)]$(ClO_4)_2$ (C).

The reduced magnetization plots (**Figure S26**) also indicate magnetic anisotropy due to the absence of a single master curve (i.e. no exact superposition of the curves obtained at different temperatures is observed in the plot).[20,21]

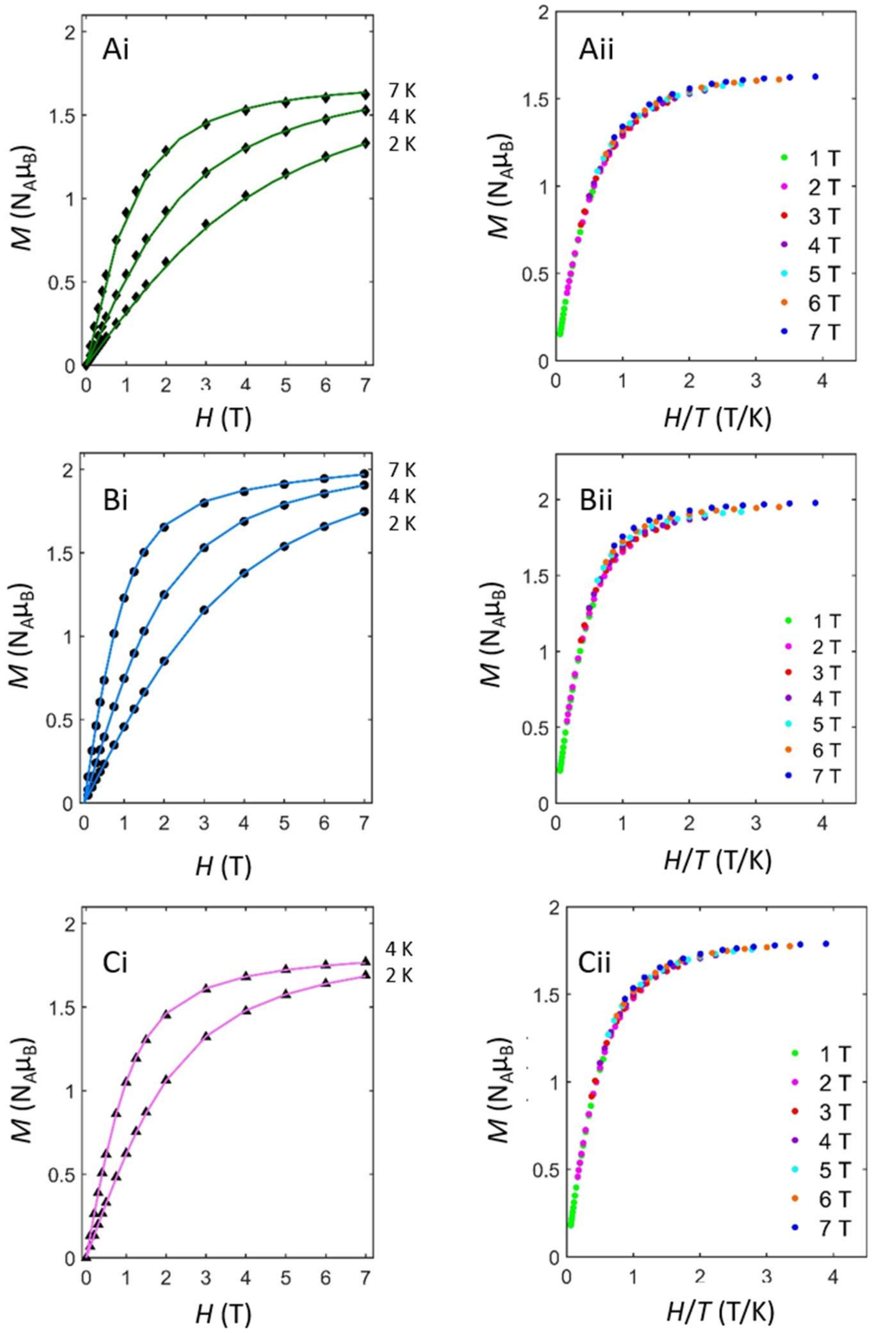

Figure S26. Magnetization vs *H* (**left)** and Reduced Magnetization (*M* vs *H*/*T*) (**right)** for [Co(**1-3**)]$(ClO_4)_2$, recorded at 2, 4 and 7 K for [Co(**1**)]$(ClO_4)_2$ (green) and [Co(**2**)]$(ClO_4)_2$ (blue) and at 2 and 4 K for [Co(**3**)]$(ClO_4)_2$ (magenta). Fits of the magnetization data were obtained with *PHI*[15] (see Table 1 in the main manuscript for parameters), where the best fits were obtained including the contribution of the low-spin species that was set to 0.45, 0.05 and 0.10 for [Co(**1-3**)]$(ClO_4)_2$, respectively.

4.3 Assessing the magnitude of *D* for [Co(**2**)](ClO_4)$_2$

In order to illustrate the validity of the large values of *D* estimated, fits with different *D* values (–20, –40 and –80 cm^{-1}) are shown in Figure S27 for [Co(**2**)](ClO_4)$_2$.

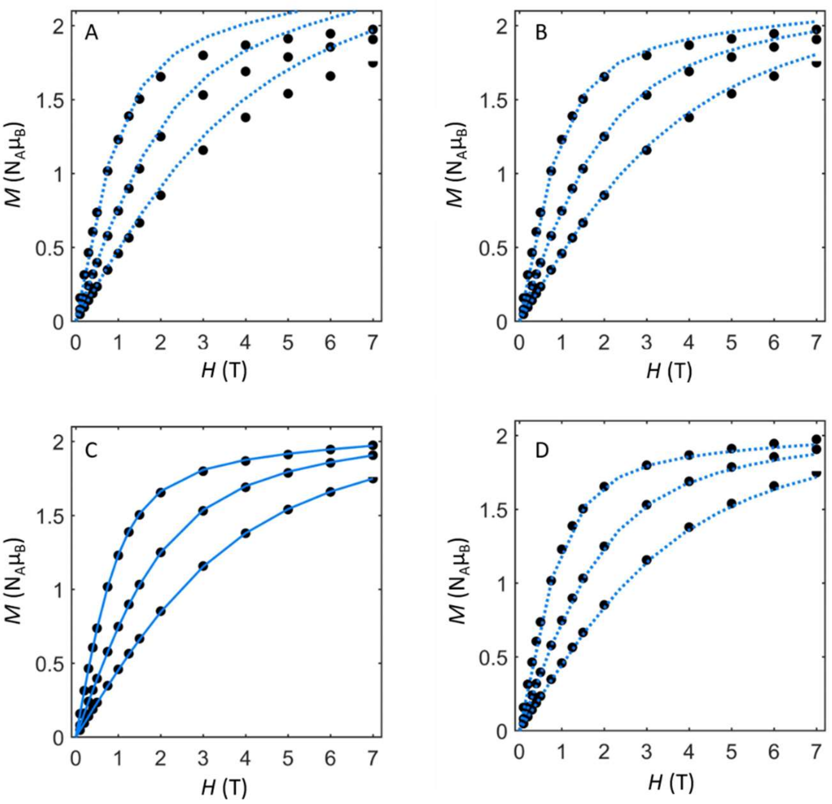

Figure S27. Fits of Magnetization vs *H* for [Co(**2**)](ClO_4)$_2$ obtained with different *D* values. *D* = –20 cm^{-1} (A), *D* = –40 cm^{-1} (B), *D* = –59 cm^{-1} (C), *D* = –80 cm^{-1} (D).

4.4 Magnetic susceptibility from the Evan's method

The Evan's method was used to determine the magnetic susceptibility of [Co(**1-3**)](ClO_4)$_2$ in solution at room temperature. A mixture of $CDCl_3$/$CHCl_3$ 50:1 (volume) was added to a coaxial tube insert and the same solvent mixture was used to prepare a 10 mM solution of the complexes. The coaxial insert was then placed in a NMR tube filled with the paramagnetic sample solution and the magnetic susceptibility were determined from the chemical shift difference of the solvent signal inside and outside the capillary tube.[22] χT product obtained with this method are 2.05 $cm^3\cdot K\cdot mol^{-1}$, 2.56 $cm^3\cdot K\cdot mol^{-1}$, and 2.29 $cm^3\cdot K\cdot mol^{-1}$ for [Co(**1-3**)](ClO_4)$_2$, respectively.

4.5 Comparison of *D* with reported literature values

Table S4. Zero-field splitting, energy barrier and τ_0 values for pentacoordinate Co^{II} complexes reported to exhibit negative *D* values and slow magnetic relaxation. See also ref. 23 for an overview of four-, five-, six-, seven- and eight-coordinate Co^{II} SIMs.

	D (cm^{-1})	U_{eff} (K)	τ_0 (s)
[{ArN=CMe}$_2$(NPh)]Co(NCS)$_2$[a]	– 28	16	$3.6 \cdot 10^{-6}$
[{ArN=CPh}$_2$(NPh)]Co(NCS)$_2$[a]	– 28	24	$5.1 \cdot 10^{-7}$
Co(terpy)Cl$_2$[b]		28	$1.1 \cdot 10^{-6}$
Co(terpy)(NCS)$_2$[b]		17	$5.9 \cdot 10^{-7}$
Co(phen)(DMSO)Cl$_2$[c]	– 17	10.4	$5.7 \cdot 10^{-9}$
[Co^{II}(tbta)N$_3$]$^{+}$[d]	– 10	14.2	$1.6 \cdot 10^{-8}$
[Co(NS$_3^{tBu}$)X]ClO$_4$ (X=Cl, Br, NCS)[e]	– 21, – 20, – 11	28.8	$2 \cdot 10^{-9}$
[Co(**1**)](ClO$_4$)$_2$ [f]	– 78	224	$0.031 \cdot 10^{-3}$
[Co(**2**)](ClO$_4$)$_2$ [f]	– 59	170	$0.140 \cdot 10^{-3}$
[Co(**3**)](ClO$_4$)$_2$ [f]	– 95	273	$0.011 \cdot 10^{-3}$
[Co(3,4-lutidine)$_4$Br]$^{-}$ [g]	not reported	40.9	$8.3 \cdot 10^{-6}$

a) Taken from reference 24. *D* values are obtained from the energy barriers rather than from magnetization/EPR data. b) Taken from reference 25. *D* values were not obtained from experimental work and the separation between energy levels was determined based on computational methods. c) Taken from reference 26 and d) reference 27. e) Experimental *D* values given (see reference 28 for calculated values). f) This work (note that U_{eff} was fixed based on the values obtained for *D*). g) Taken from reference 29.

4.6 Field dependence of the magnetisation relaxation time extracted from ac susceptibility data at 2 K and several applied fields

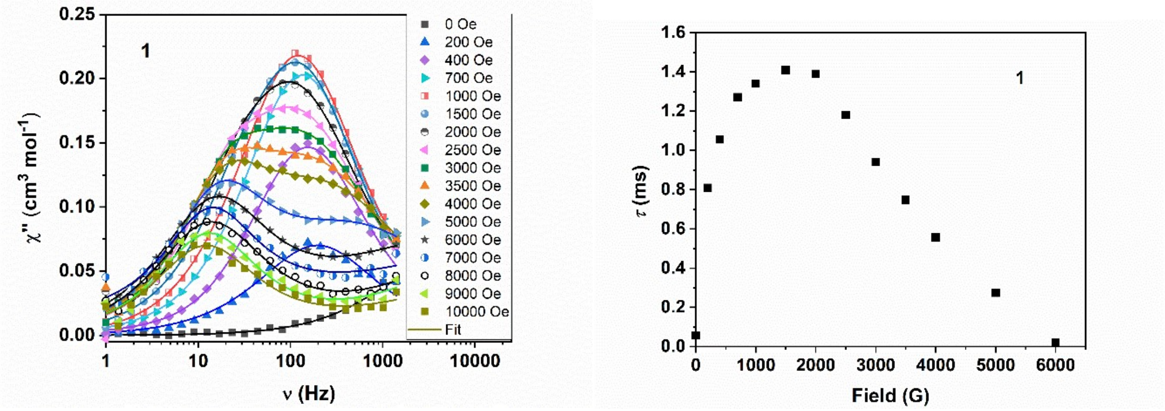

Figure S28. *Left.* Frequency dependence of the out-of-phase χ'' component of the a.c. magnetic susceptibility collected for [Co(**1**)](ClO_4)$_2$ under different applied d.c. fields at 2 K. Solid lines represent the best fit of the data using an extended Debye model. Note that the peak at ~10 Hz under high magnetic fields is temperature independent and noticeable below 2.4 K only. *Right.* Field dependence of the relaxation time extracted from the same data, and associated with the out-of-phase maxima appearing at higher frequency.

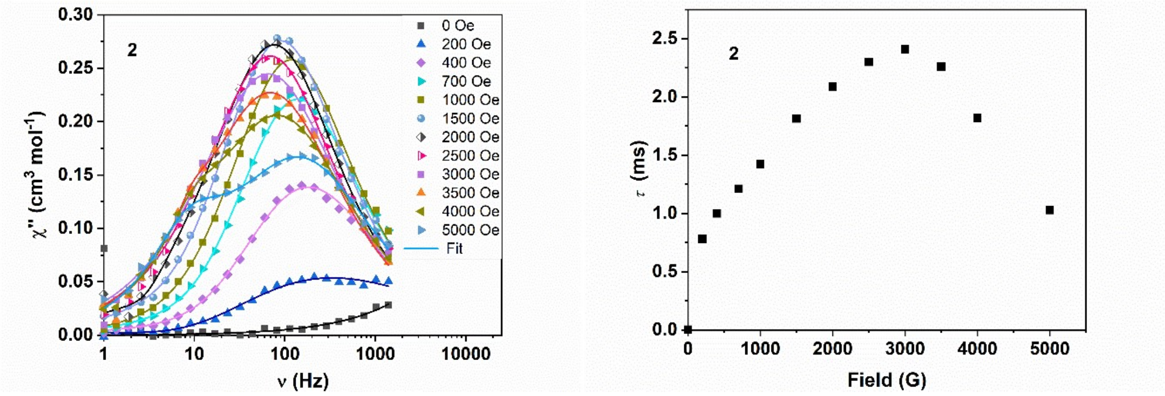

Figure S29. *Left.* Frequency dependence of the out-of-phase χ'' component of the a.c. magnetic susceptibility collected for [Co(**2**)](ClO_4)$_2$ under different applied d.c. fields at 2 K. Solid lines are best fits of the data using an extended Debye model. *Right.* Field dependence of the relaxation time extracted from the same data, and associated with the out-of-phase maxima appearing at higher frequency.

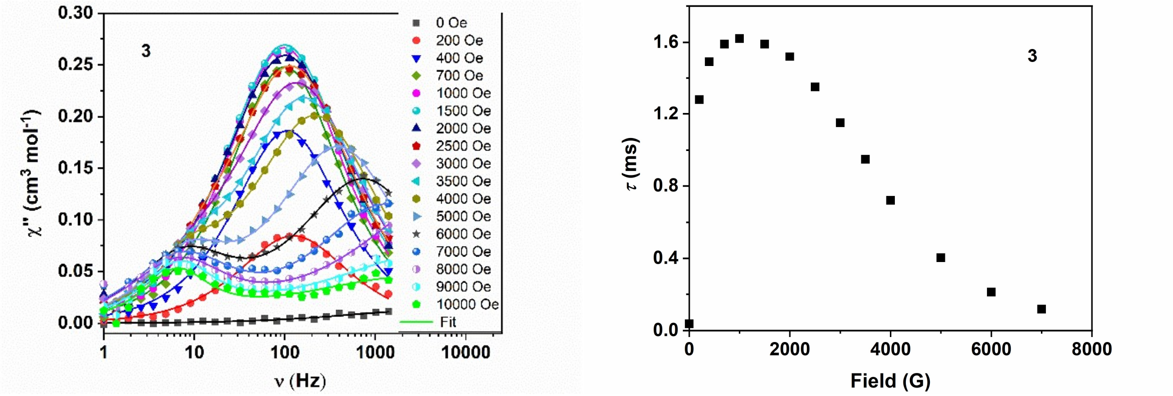

Figure S30. *Left.* Frequency dependence of the out-of-phase χ'' component of the a.c. magnetic susceptibility collected for [Co(**3**)](ClO_4)$_2$ under different applied d.c. fields at 2 K. Solid lines are best fits of the data using an extended Debye model. We note that the peak at ~10 Hz under high magnetic fields is temperature independent and noticeable below 2.4 K only. *Right.* Field dependence of the relaxation time extracted from the same data, and associated with the out-of-phase maxima appearing at higher frequency.

4.7 Temperature dependence of the magnetic relaxation time extracted from ac susceptibility data recorded under the optimum applied field and fitting of the data

The extended Debye model was used to fit the in-phase susceptibility (χ', equation 1) and the out-of-phase susceptibility (χ'', equation 2) at each temperature, where ω = 2πν (Hz). The free parameters χ_S (adiabatic susceptibility), χ_T (isothermal susceptibility), α (distribution parameter), and τ (relaxation time) were extracted from the fit. α and τ values are listed in Tables 1-3 for complexes [Co(**1-3**)](ClO_4)$_2$ below.

The χ' and χ'' values estimated from the fit were then plotted to generate the Cole-Cole plot (**Figure S31**).

$$\chi'(\omega) = \chi_S + \frac{(\chi_T-\chi_S)[1+(\omega\tau)^{1-\alpha}\sin(\pi\alpha/2)]}{1+2(\omega\tau)^{1-\alpha}\sin(\pi\alpha/2)+(\omega\tau)^{2-2\alpha}} \quad (1)$$

$$\chi''(\omega) = \frac{(\chi_T-\chi_S)[(\omega\tau)^{1-\alpha}\cos(\pi\alpha/2)]}{1+2(\omega\tau)^{1-\alpha}\sin(\pi\alpha/2)+(\omega\tau)^{2-2\alpha}} \quad (2)$$

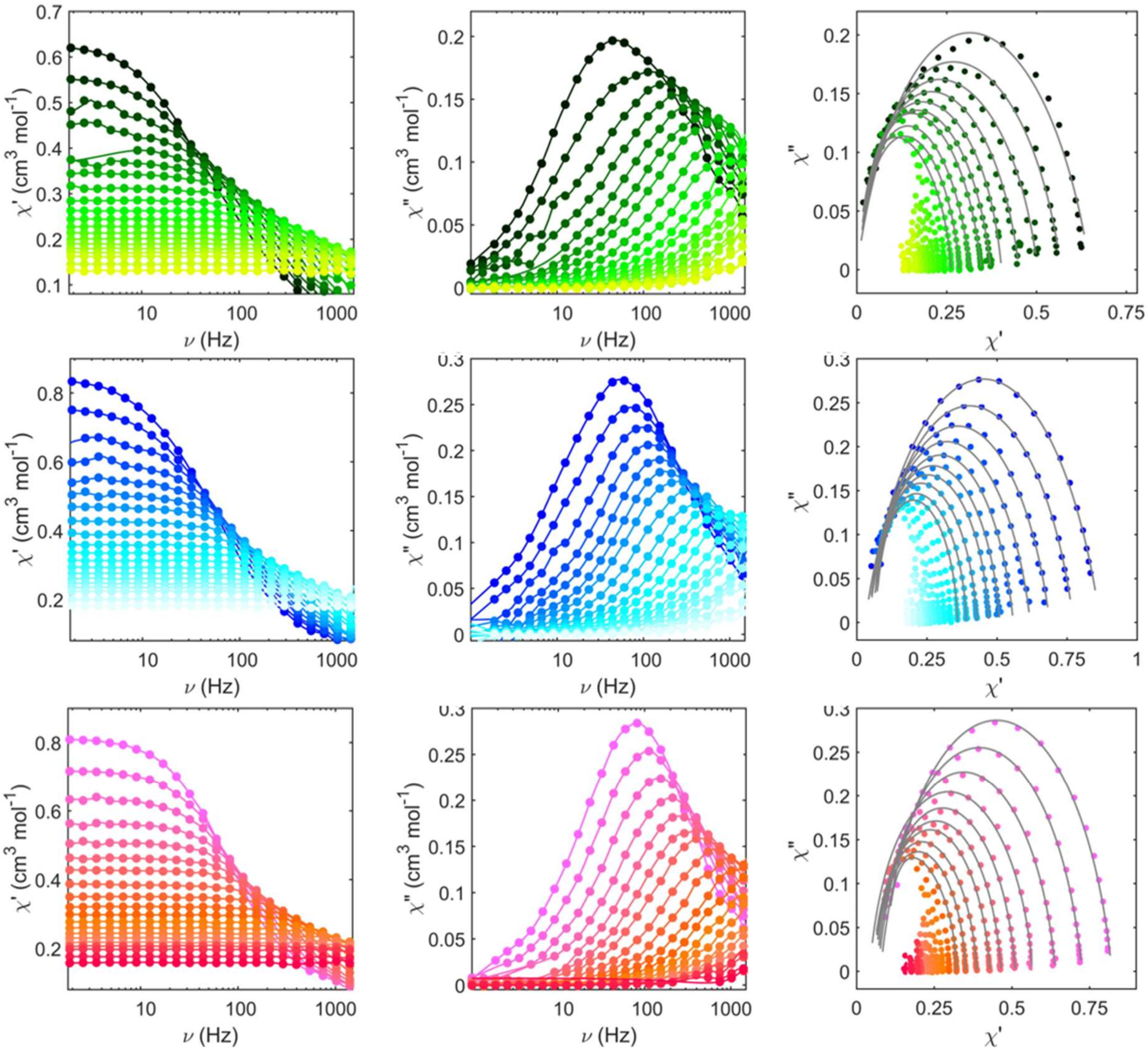

Figure S31. Frequency dependence of in-phase (left column) and out-of-phase (middle column) susceptibility and Cole-Cole plots (right column, fits in grey) for complexes [Co(**1**)]$(ClO_4)_2$ (green), [Co(**2**)]$(ClO_4)_2$ (blue), and [Co(**3**)]$(ClO_4)_2$ (magenta). Measurements were carried out under an applied field of 2500 Oe for [Co(**1**)]$(ClO_4)_2$ and [Co(**2**)]$(ClO_4)_2$ and 1000 Oe for [Co(**3**)]$(ClO_4)_2$ in the temperature range of 1.8-10 K.

Table S5. Parameters of the extended Debye model for [Co(**1**)]$(ClO_4)_2$.See **Figure 28** for the resulting fit.

T (K)	τ *10^{-2} s (from χ')	τ *10^{-2} s (from χ'')	α (from χ')	α (from χ'')
1.8	2.777	2.606	0.285	0.291
2.1	1.759	1.572	0.284	0.317
2.4	1.154	0.9834	0.265	0.307
2.7	0.788	0.675	0.252	0.289
3	0.5534	0.473	0.213	0.267
3.3	0.402	0.344	0.215	0.246
3.6	0.304	0.251	0.202	0.231
4	0.210	0.179	0.200	0.210
4.4	0.143	0.129	0.188	0.200
4.8	0.104	0.081	0.165	0.198

Table S6. Parameters of the extended Debye model for $[Co(\mathbf{2})](ClO_4)_2$. See **Figure 28** for the resulting fit.

T (K)	τ *10^{-2} s (from χ')	τ *10^{-2} s (from χ'')	α (from χ')	α (from χ'')
1.8	2.810	2.842	0.258	0.253
2.1	2.177	2.213	0.245	0.251
2.4	1.535	1.533	0.237	0.237
2.7	1.225	1.160	0.214	0.215
3	0.937	0.937	0.188	0.193
3.3	0.798	0.735	0.155	0.183
3.6	0.657	0.596	0.129	0.169
4	0.487	0.438	0.119	0.143
4.4	0.384	0.378	0.092	0.110
4.8	0.281	0.257	0.075	0.096

Table S7. Parameters of the extended Debye model for [Co(**3**)](ClO_4)$_2$. See **Figure 28** for the resulting fit.

T (K)	τ *10^{-2} s (from χ')	τ *10^{-2} s (from χ'')	α (from χ')	α (from χ'')
1.8	1.958	1.968	0.258	0.253
2.1	1.449	1.435	0.245	0.251
2.4	1.071	1.031	0.237	0.237
2.7	0.801	0.765	0.214	0.215
3	0.602	0.574	0.188	0.193
3.3	0.444	0.433	0.155	0.183
3.6	0.345	0.336	0.129	0.169
4	0.260	0.235	0.119	0.143
4.4	0.194	0.178	0.092	0.110
4.8	0.157	0.128	0.075	0.096

4.8 Fitting parameters for temperature-dependent relaxation (fixed U_{eff})

Relaxation times in Figure 4B in the main paper were fitted with following equation:

$$\tau^{-1} = a \cdot T + b \cdot T^{n} + \tau_0^{-1} e^{-Uef}/_{kT}. \quad (3)$$

The curvature of the plot log(τ) vs T^{-1} (Figure 4B) pointed to the presence of several relaxation pathways that cannot be fitted with an Orbach process (third term in equation 3) alone and contributions from additional relaxation pathways were therefore considered. To avoid overparameterization, QTM was set to zero (and hence is not included in Equation 3): this assumption is validated by the dependence of QTM rate to H^{-2}, which causes suppression of this relaxation phenomenon under an applied field. For the same reason, the direct process (first term in equation 3) was considered in the fitting, due to the dependence of the direct coupling between the |+3/2> and |–3/2> states to H^4. The exponent of the Raman mechanism (second term in equation 3), *n*, was set here as fit parameter. U_{eff} was not treated as a variable and calculated using the experimentally obtained *D* values (2*D*).

Table S8. Parameters obtained from fitting of the relaxation times of [Co(**1-3**)](ClO_4)$_2$.

	[Co(**1**)](ClO_4)$_2$	[Co(**2**)](ClO_4)$_2$	[Co(**3**)](ClO_4)$_2$
a ($K^{-1}\cdot s^{-1}$)	113.01±800	185.81±785	33.35±520
b ($K^{-n}\cdot s^{-1}$)	18.415±33	6.536±36	69.576±264
n	3.95±0.97	3.82±3	2.87±1.7
τ_0 (ms)	0.0312±0.07	0.1401±0.3	0.0109±0.02
U_{eff} (K)	224	170	273

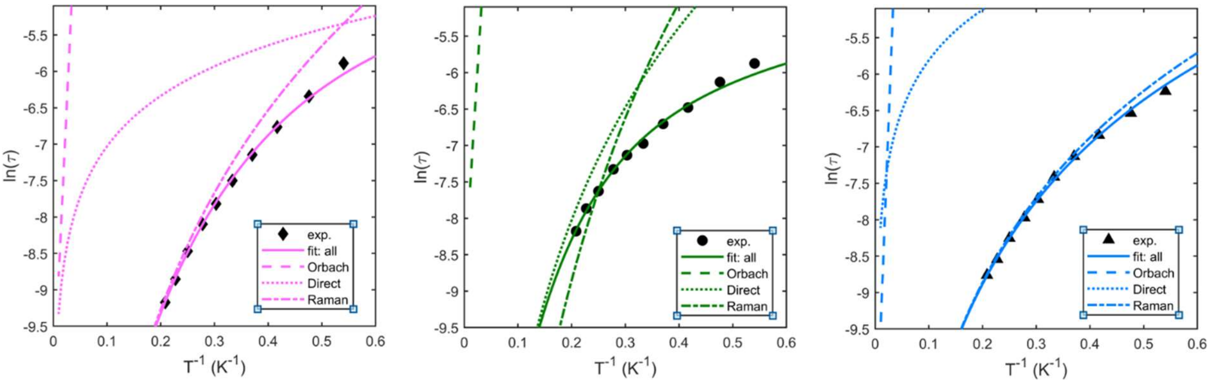

Figure S32. Temperature dependence of the relaxation times (τ for complexes **1** (♦), **2** (●) and **3** (▲) with fits of individual relaxation processes as indicated (see Table S8 for fitting parameters).

5 Calculations

5.1 De novo modelling and comparison with the corresponding SCXRD structures

A trigonal bipyramidal model of the bipy-Co-pyridine bis triazole core was prepared (Spartan '10, Wavefunction Inc.) and the coordination sphere frozen. The rest of the rotaxane framework was added and a mechanical (MMF) minimisation applied first with the coordination sphere frozen, then with the bipyridine and pyridine bis-triazole units constrained to be perpendicular and the bipyridine unit constrained to be planar. The constraints were then removed, and the system minimised first using MMF, then using PM6. In the case of [Co(**3**)]$^{2+}$, an O-Co bond was added after the constraints had been removed and the system re-minimized (MMF). The resulting models were fully optimised using Gaussian '09[30] with the CAMB3LYP density functional, first with 3-21G basis set for light atoms and the LAND2ZP pseudopotential and basis set for Co and then with the 6-31G(d) basis set for light atoms. The models were initially optimised in the LS configuration then in HS configuration.

Comparison of the coordination sphere of the *de novo* modelled structures with those obtained by SCXRD revealed a reasonable level of agreement (Tables S9 and S10). The coordinates of the models obtained are supplied as additional electronic supporting information.

Table S9. Bond angles (°) of the calculated (HS) *de novo* structures and their root-mean-square deviation (RMSD) from the corresponding angles in the SCXRD structures of $[Co(\mathbf{1\text{-}3})](ClO_4)_2$.

	a_1	a_2	a_3	a_4	b_1	b_2	b_3	b_4	RMSD
$[Co(\mathbf{1})]^{2+}$ (HS)[a]	96.18	107.17	76.16	76.01	80.30	137.58	103.76	95.24	5.4[a]
$[Co(\mathbf{1})]^{2+}$ (LS)[a]	99.02	97.60	80.88	80.95	80.40	113.38	96.84	94.26	1.1[a]
$[Co(\mathbf{2})]^{2+}$	104.65	98.18	77.28	75.17	78.28	130.57	111.17	88.86	4.3
$[Co(\mathbf{3})]^{2+}$	95.14	112.9	75.70	76.26	77.00	109.56	99.80	88.28	5.4

[a]RMSD of the calculated HS and LS structures are reported with respect the corresponding experimental set of values (see Table S1) found by SCXRD. Distances and angles defined in **Figure S22**.

Table S10. Bond lengths (Å) of the calculated (HS) *de novo* structures and their root-mean-square deviation (RMSD) from the corresponding distances in the SCXRD structures of $[Co(\mathbf{1\text{-}3})](ClO_4)_2$.

Molecules	d_1	d_2	d_3	d_4	d_5	RMSD
$[Co(\mathbf{1})]^{2+}$ (HS)	2.073	2.08	2.101	2.132	2.152	0.039[a]
$[Co(\mathbf{1})]^{2+}$ (LS)	2.206	1.932	1.934	1.952	1.961	0.050[a]
$[Co(\mathbf{2})]^{2+}$	2.093	2.08	2.094	2.124	2.185	0.016
$[Co(\mathbf{3})]^{2+}$	2.265	2.049	2.106	2.129	2.180 (2.323)[b]	0.169

[a]The standard deviations of the calculated HS and LS structures are reported with respect the corresponding experimental set of values (see Table S1) found by SCXRD. [b]Distance Co-O in the octahedral $[Co(\mathbf{3})]^{2+}$ complex. Distances and angles defined in **Figure S22**.

5.2 Comparison between the experimental SCXRD structures and optimised molecular models derived from them

Single molecules were extracted from the SCXRD structures and fully optimised in both high spin (HS) and low spin (LS) configurations using the Gaussian program.[30] These calculations were performed using the CAMB3LYP density functional, the 6-31G(d) basis set for light atoms and the LAND2ZP pseudopotential and basis set for Co. Based on this modelling a HS-LS energy gaps were obtained as follows: $[Co(\mathbf{1})]^{2+}$ = 2.7 kJ/mol, $[Co(\mathbf{2})]^{2+}$ = 46 kJ/mol and $[Co(\mathbf{3})]^{2+}$ = 11.8 kJ/mol. $[Co(NH_3)_5]^{2+}$ and $[Co(NH_3)_5(H_2O)]^{2+}$ models were also considered, they were fully or partially optimised keeping the angles and intramolecular distances fixed to the experimental data with full relaxation of the hydrogens.

The coordinates of the models obtained are supplied as additional electronic supporting information.

Complexes [Co(**1**)]$^{2+}$ and [Co(**2**)]$^{2+}$ display distorted squared pyramidal geometries (**Figure S33** and

Table S11). In the case of [Co(**2**)]$^{2+}$, there are close contacts between the metal and the π clouds from the phenyl groups located at 3.19 and 3.26 Å. There are also π-π interactions between the phenyl and the triazole rings. In the case of [Co(**1**)]$^{2+}$, there is close contact between one C-H and the Co centre (261 Å). These intramolecular interactions determine the distortion of the coordination sphere and consequently the splitting of the d-orbitals. In contrast with [Co(**1**)]$^{2+}$ and [Co(**2**)]$^{2+}$, [Co(**3**)]$^{2+}$ shows distorted octahedral geometry with direct coordination of one of the O from the macrocycle. In [Co(**3**)]$^{2+}$, the axial Co-O and Co-N bonds (2.3 Å) are elongated with respect to the equatorial Co-N bonds 2.1 (**Figure S23** and **Table S12**).

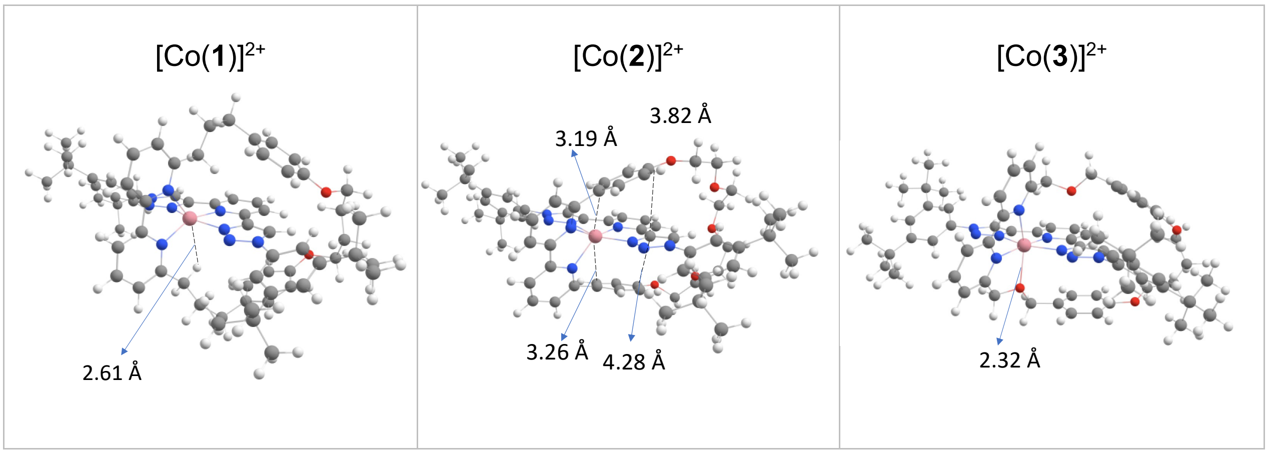

Figure S33. Geometry of the HS complexes.

To address the role of intramolecular interactions on the distortion of the Co coordination sphere, we build a simplified model containing the interlocked structure common to all complexes (**model A**). We also considered a model with an octahedral geometry and coordination to O, which is closest to [Co(**3**)]$^{2+}$ (**model B**). These models were completely optimised at the same level of theory. The obtained geometry of **model A** resembles quite closely the geometry of [Co(**1**)]$^{2+}$, suggesting that C-H··· Co interaction does not significantly contribute to the distortion of the geometry.

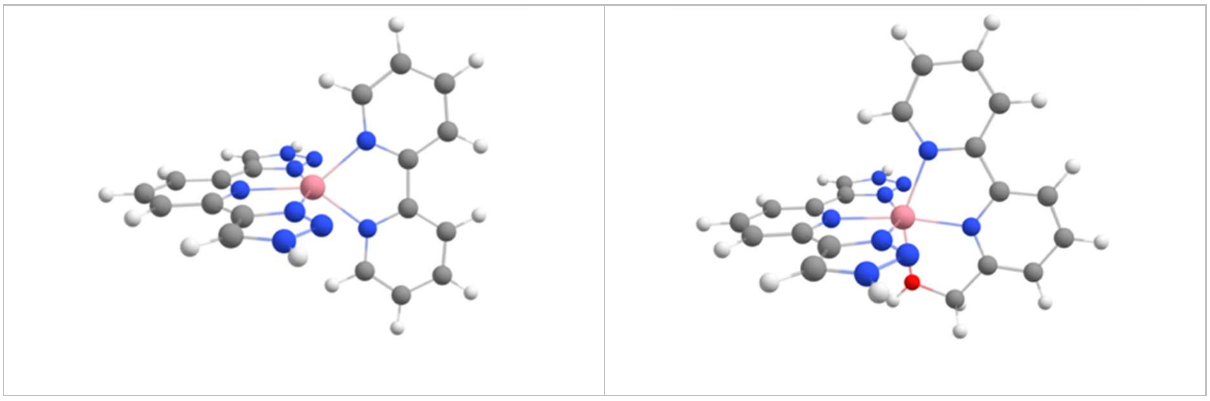

Figure S34. Optimised structure for **model A** (left) and **model B** (right).

Table S11. Bond angles (°) of the calculated (HS) structures of [Co(**1**-**3**)](ClO_4)$_2$ derived from the SCXRD data and truncated models **A** and **B** and their root-mean-square deviation (RMSD) from the corresponding angles of the parent SCXRD structures.

	a_1	a_2	a_3	a_4	b_1	b_2	b_3	b_4	RMSD
[Co(**1**)]$^{2+}$	104.19	97.5	75.89	77.15	80.12	127.68	104.58	95.49	3.8[a]
[Co(**2**)]$^{2+}$	105.08	98.07	77.62	74.37	78.66	124.65	114.38	85.9	3.6
[Co(**3**)]$^{2+}$	98.17	109.46	75.7	76.55	76.72	112.66	101.18	88.26	4.5
**model A**	103.44	98.14	75.93	75.93	79.37	140.31	103.45	98.14	4.6[b]
**model B**	101.9	108.87	74.79	74.89	77.54	111.02	94.5	91.44	3.1[c]

[a]Reported with respect the experimental values corresponding to a high spin electronic structure. [b] The smallest deviation (shown) with respect to the optimised structures was obtained for [Co(**1**)]$^{2+}$ ([Co(**2**)]$^{2+}$: 8.1°, [Co(**3**)]$^{2+}$: 11.4°). [c]RMSD with respect to the calculated structure of [Co(**3**)]$^{2+}$. Angles as defined in **Figure S22**.

Table S12. Bond lengths (°) of the calculated (HS) structures of [Co(**1**-**3**)](ClO_4)$_2$ derived from the SCXRD data and truncated models **A** and **B** and their root-mean-square deviation (RMSD) from the corresponding distances in the parent SCXRD structures.

Molecules	d_1	d_2	d_3	d_4	d_5	RMSD
[Co(**1**)]$^{2+}$	2.077	2.064	2.09	2.105	2.157	0.029
[Co(**2**)]$^{2+}$	2.103	2.081	2.098	2.125	2.214	0.021
[Co(**3**)]$^{2+}$	2.25	2.05	2.102	2.104	2.183 (2.315)[a]	0.164
**model A**	2.066	2.066	2.105	2.153	2.153	0.023[b]
**model B**	2.136	2.063	2.142	2.176	2.192 (2.178)[a]	0.084[c]

[a]Distance Co-O in the octahedral [Co(**3**)]$^{2+}$ complex. [b] The smallest deviation with respect to the optimised structures was obtained for [Co(**1**)]$^{2+}$ ([Co(**2**)]$^{2+}$: 0.035, [Co(**3**)]$^{2+}$: 0.087). [c]RMSD with respect to the calculated [Co(**3**)]$^{2+}$ structure. Distances as defined in **Figure S22**.

In general, the agreement between the calculated and experimental structures is very good. The larger deviations are obtained for the complex [Co(**3**)]$^{2+}$, which may in part be related to the lower resolution of the corresponding molecular crystal.

5.3 Calculation of *D* values

Excited states calculations were performed using state average CASSCF and TZVP basis set as implemented in the Orca program.[31] The active space considered 7 electrons in 5 orbitals (d orbitals). Five roots were included in the state average (SA-5-CASSCF(7,5)), relativistic corrections and spin-orbit couplings were taken into account. NEVPT2 calculations were done for smaller model systems. For the SCXRD-derived models, we also perform CASSCF and NEVPT2 calculations considering ten quartets for the state-average (SA-10-CASSCF(7,5)).

Prediction of *D* values is a challenging task. These complexes have small gaps between the ground and excited state, consequently TDDFT methods are not the best choice to represent their multiconfigurational electronic structures. To provide a more accurate description, we used CASSCF method for the calculation of the excited states and *D* values (

Table **S13**). The chosen active space (7,5), which only includes the d orbitals from the metal should be good enough to describe the lower energy excited states and provide qualitative values of *D*.

The values obtained for $[Co(\mathbf{1})]^{2+}$ and $[Co(\mathbf{2})]^{2+}$ are overestimated with respect to the experimental ones, while in the case of $[Co(\mathbf{3})]^{2+}$ the *D* value is underestimated by 20 cm^{-1}. The calculations do not reproduce the experimental trend for the *D* values ($[Co(\mathbf{3})]^{2+} > [Co(\mathbf{1})]^{2+} > [Co(\mathbf{2})]^{2+}$), however in line with the experiments, all molecules have relatively large negative values of *D*. Large negative values of *D* are associated with significant magnetic anisotropy, which is a requirement for single-molecule magnets.[32]

Table S13. *D* values obtained using the effective Hamiltonian spin orbit coupling approach, ground state-excited state energy gaps and average SOC matrix elements at the SA-5-CASSCF/TZVP level of theory. All values are given in cm^{-1}. The values for the *de novo* complexes are given in parenthesis.

	**D**	**Largest contribution to D**	**Experimental values**	**Ground-first excited state gap**	**Average SOC matrix element (ground/first excited state)**
$[Co(\mathbf{1})]^{2+}$	-106.1 (-80.0)	-129.2 (-109.2)	-78	443.6 (508.3)	324.5 (297.1)
$[Co(\mathbf{2})]^{2+}$	-72.9 (-71.2)	-92.0 (-111.7)	-59	742.0 (780.3)	305.2 (304.1)
$[Co(\mathbf{3})]^{2+}$	-73.3 (-80.0)	-95.7 (-100.8)	-95	677.5 (627.6)	298.7 (301.4)

In all cases, the largest contribution to the *D* tensor comes from the first excited state. The calculated values *D* correlate with the ground-first excited state gaps and the corresponding SOC matrix elements. The *D* value obtained for **model A** is similar to that obtained for $[Co(\mathbf{1})]^{2+}$. This suggests that the *D* values are determined by the deformation of geometry in the Co environment. To further confirm this hypothesis, we created a series of models based on **model A**, but keeping the distances and angles from the calculated or experimental values (*SCXRD-derived models*,

Table S14). The use of smaller models also allows for considering the effect of dynamic correlation, we compare the *D* values of small models obtained with CASSCF and NEVPT2 calculations. Both sets of values are very similar showing that the deviations from the experimental data are not related to the lack of dynamic correlation in CASSCF.

Table S14. *D* (cm^{-1}) values calculated for the reduced models (**model A** keeping the geometrical parameters as in the original optimised geometries).

	SA-5-CASSCF	NEVPT2
optimised geometries		
$[Co(\mathbf{1})]^{2+}$	-108.1	-95.2
$[Co(\mathbf{2})]^{2+}$	-89.3	-89.9
$[Co(\mathbf{3})]^{2+}$	-78.1	-78.7
**model A**	-93.9	-79.9
$\mathbf{[Co(NH_3)_5]^{2+a}}$	6.9	9.3
$\mathbf{[Co(NH_3)_5]^{2+b}}$	-99.1	-98.4

[a] $\mathbf{[Co(NH_3)_5]^{2+}}$ complex was optimised without symmetry restrictions.[b] $\mathbf{[Co(NH_3)_5]^{2+}}$ complex with the geometry of **model A**.

We have also considered the complex $\mathbf{[Co(NH_3)_5]^{2+}}$, which has been previously used to analyse the effect of geometry distortion in singlet magnets.[21] If the angles and distances in complex $[Co(NH_3)_5]^{2+}$ are kept as those in **model A**, the obtained value of *D* is -99.1 cm^{-1} which is close to the value obtained for **model A** (-93.1 cm^{-1}). If we modify the geometry of the $\mathbf{[Co(NH_3)_5]^{2+}}$ and $\mathbf{[Co(NH_3)_5(H_2O)]^{2+}}$ complexes and use the experimental geomerical parameters only optimising the positions of the hydrogens, the agreement between the experimental and calculated D values is very good and we even obtain the right trend ($[Co(\mathbf{3})]^{2+} > [Co(\mathbf{1})]^{2+} > [Co(\mathbf{2})]^{2+}$).

Table S15. *D* (cm^{-1}) values calculated for the *SCXRD-derived models*. The values obtained using 10 roots in the state average are shown in parentheses.

	CASSCF	NEVPT2	Experimental
$\mathbf{[Co(NH_3)_5]^{2+}}$ **model**			
$[Co(\mathbf{1})]^{2+}$	-65.3<br>(-66.5)	-61.9<br>(-63.3)	-78
$[Co(\mathbf{2})]^{2+}$	-58.1<br>(-59.4)	-54.5<br>(-54.8)	-59
$\mathbf{[Co(NH_3)_5(H_2O)]^{2+}}$ **model**			
$[Co(\mathbf{3})]^{2+}$	-95.9	-90.3	-95

	(-92.0)	(-88.0)	

The disagreement between the experimental and predicted values of D for the larger models is due to the use optimised geometries in the gas phase while experiments were performed in the solid state (

Table **S13** and

Table S14). Our calculations show that the *D* values are mainly determined by the geometry of Co coordination sphere and their distortion from the ideal square pyramidal/octahedral geometry. These simplified models also allowed the role of dynamic correlation to be examined; comparison of the results of the CASSCF calculations with NEVPT2 modelling revealed similar values, suggesting that the lower level of theory is sufficient to describe these systems. The values of D obtained using 10 or 5 roots in the state-average are very similar. To be consistent with the results reported for the rotaxane complexes, we report the D values obtained with SA-5-CASSCF(7,5) in the main text.

For the analysis of the values of D, we analysed the wavefunctions of the ground (Q_0) and the first excited states (Q_1) obtained with CASSCF. These complexes are highly multiconfigurational, the contributions of the main electronic configurations for both states are reported below.

Table S12. Contributions of most important configurations to the ground (Q_0) and excited state configurations (Q_1) of the *SCXRD derived models*.

	Weight (%)		
**Q_0**	[Co(**1**)]$^{2+}$	[Co(**2**)]$^{2+}$	[Co(**3**)]$^{2+}$
$(d_{xz})^2(d_{yz})^2(d_{xy})^1(d_{x^2-y^2})^1(d_{z^2})^1$	50.2	50.3	7.5
$(d_{xz})^1(d_{yz})^2(d_{xy})^2(d_{x^2-y^2})^1(d_{z^2})^1$	20.2	14.3	35.5
$(d_{xz})^2(d_{yz})^1(d_{xy})^2(d_{x^2-y^2})^1(d_{z^2})^1$	16.6	21.2	52.2
$(d_{xz})^1(d_{yz})^1(d_{xy})^2(d_{x^2-y^2})^2(d_{z^2})^1$	8.3	8.6	7.9
**Q_1**			
$(d_{xz})^2(d_{yz})^2(d_{xy})^1(d_{x^2-y^2})^1(d_{z^2})^1$	20.3	7.1	3.5
$(d_{xz})^1(d_{yz})^2(d_{xy})^2(d_{x^2-y^2})^1(d_{z^2})^1$	68.5	76.0	59.6
$(d_{xz})^1(d_{yz})^2(d_{xy})^2(d_{x^2-y^2})^1(d_{z^2})^1$	0.0	0.4	30.1
$(d_{xz})^1(d_{yz})^2(d_{xy})^1(d_{x^2-y^2})^1(d_{z^2})^2$	5.5	6.3	0.0

References

(1) Pigorsch, A.; Köckerling, M. The Crystallization of Extended Niobium-Cluster Framework Compounds: A Novel Approach Using Ionic Liquids. *Cryst. Growth Des.* **2016**, *16* (8), 4240–4246. https://doi.org/10.1021/acs.cgd.6b00225.

(2) Potter, G. T.; Jayson, G. C.; Miller, G. J.; Gardiner, J. M. An Updated Synthesis of the Diazo-Transfer Reagent Imidazole-1-Sulfonyl Azide Hydrogen Sulfate. *J. Org. Chem.* **2016**, *81* (8), 3443–3446. https://doi.org/10.1021/acs.joc.6b00177.

(3) Lewis, J. E. M.; Bordoli, R. J.; Denis, M.; Fletcher, C. J.; Galli, M.; Neal, E. A.; Rochette, E. M.; Goldup, S. M. High Yielding Synthesis of 2,2′-Bipyridine Macrocycles, Versatile Intermediates in the Synthesis of Rotaxanes. *Chem. Sci.* **2016**, *7* (5), 3154–3161. https://doi.org/10.1039/c6sc00011h.

(4) Yuan, J.; Fang, X.; Zhang, L.; Hong, G.; Lin, Y.; Zheng, Q.; Xu, Y.; Ruan, Y.; Weng, W.; Xia, H.; Chen, G. Multi-Responsive Self-Healing Metallo-Supramolecular Gels Based on "Click" Ligand. *J. Mater. Chem.* **2012**, *22* (23), 11515. https://doi.org/10.1039/c2jm31347b.

(5) Li, Y.; Flood, A. H. Pure C□H Hydrogen Bonding to Chloride Ions: A Preorganized and Rigid Macrocyclic Receptor. *Angew. Chemie Int. Ed.* **2008**, *47* (14), 2649–2652. https://doi.org/10.1002/anie.200704717.

(6) Cirulli, M.; Kaur, A.; Lewis, J. E. M.; Zhang, Z.; Kitchen, J. A.; Goldup, S. M.; Roessler, M. M. Rotaxane-Based Transition Metal Complexes: Effect of the Mechanical Bond on Structure and Electronic Properties. *J. Am. Chem. Soc.* **2019**, *141* (2), 879–889. https://doi.org/10.1021/jacs.8b09715.

(7) Cirulli, M.; Kaur, A.; Lewis, J. E. M.; Zhang, Z.; Kitchen, J. A.; Goldup, S. M.; Roessler, M. M. Rotaxane-Based Transition Metal Complexes: Effect of the Mechanical Bond on Structure and Electronic Properties. *J. Am. Chem. Soc.* **2019**, *141* (2). https://doi.org/10.1021/jacs.8b09715.

(8) Hayami, S.; Komatsu, Y.; Shimizu, T.; Kamihata, H.; Lee, Y. H. Spin-Crossover in Cobalt(II) Compounds Containing Terpyridine and Its Derivatives. *Coordination Chemistry Reviews*. Elsevier September 1, 2011, pp 1981–1990. https://doi.org/10.1016/j.ccr.2011.05.016.

(9) Sheldrick, G. M. SHELXT - Integrated Space-Group and Crystal-Structure Determination. *Acta Crystallogr. Sect. A* **2015**, *71* (1), 3–8. https://doi.org/10.1107/S2053273314026370.

(10) Dolomanov, O. V; Bourhis, L. J.; Gildea, R. J.; Howard, J. A. K.; Puschmann, H. OLEX2: A Complete Structure Solution, Refinement and Analysis Program. *J. Appl. Crystallogr.* **2009**, *42* (2), 339–341. https://doi.org/10.1107/S0021889808042726.

(11) Stoll, S.; Schweiger, A. EasySpin, a Comprehensive Software Package for Spectral Simulation and Analysis in EPR. *J. Magn. Reson.* **2006**, *178* (1), 42–55. https://doi.org/10.1016/j.jmr.2005.08.013.

(12) Addison, A. W.; Rao, T. N.; Reedijk, J.; van Rijn, J.; Verschoor, G. C., Synthesis, Structure, and Spectroscopic Properties of Copper(Ii) Compounds Containing Nitrogen–Sulphur Donor Ligands; the Crystal and Molecular Structure of Aqua[1,7-Bis(N-Methylbenzimidazol-2′-Yl)-2,6-Dithiaheptane]Copper(Ii) Perchlorate. *J. Chem. Soc., Dalton Trans.* **1984**, 1349-1356. https://doi.org/10.1039/DT9840001349.

(13) Ketkaew, R.; Tantirungrotechai, Y.; Harding, P.; Chastanet, G.; Guionneau, P.; Marchivie, M.; Harding, D. J., Octadist: A Tool for Calculating Distortion Parameters in Spin Crossover and Coordination Complexes. *Dalton Trans* **2021**, *50*, 1086-1096. https://doi.org/10.1039/D0DT03988H.

(14) Palion-Gazda, J.; Machura, B.; Kruszynski, R.; Grancha, T.; Moliner, N.; Lloret, F.; Julve, M., Spin Crossover in Double Salts Containing Six- and Four-Coordinate Cobalt(Ii) Ions. *Inorg Chem* **2017**, *56*, 6281-6296. https://doi.org/10.1021/acs.inorgchem.7b00360.

(15) Chilton, N. F.; Anderson, R. P.; Turner, L. D.; Soncini, A.; Murray, K. S. PHI: A Powerful New Program for the Analysis of Anisotropic Monomeric and Exchange-Coupled Polynuclear d- and f-Block Complexes. *J. Comput. Chem.* **2013**, *34* (13), 1164–1175. https://doi.org/10.1002/jcc.23234.

(16) Yamane, T.; Sugisaki, K.; Nakagawa, T.; Matsuoka, H.; Nishio, T.; Kinjyo, S.; Mori, N.; Yokoyama, S.; Kawashima, C.; Yokokura, N.; Sato, K.; Kanzaki, Y.; Shiomi, D.; Toyota, K.; Dolphin, D. H.; Lin, W.-C.; McDowell, C. A.; Tadokoro, M.; Takui, T. Analyses of Sizable ZFS and Magnetic Tensors of High Spin Metallocomplexes. *Phys. Chem. Chem. Phys.* **2017**, *19* (36), 24769–24791. https://doi.org/10.1039/C7CP03850J.

(17) Harvey, J. D.; Ziegler, C. J.; Telser, J.; Ozarowski, A.; Krzystek, J. High-Frequency and -Field EPR Investigation of a Manganese(III) N-Confused Porphyrin Complex, [Mn(NCTPP)(Py)2]. *Inorg. Chem.* **2005**, *44* (13), 4451–4453. https://doi.org/10.1021/ic0506759.

(18) Sottini, S.; Poneti, G.; Ciattini, S.; Levesanos, N.; Ferentinos, E.; Krzystek, J.; Sorace, L.; Kyritsis, P. Magnetic Anisotropy of Tetrahedral CoIISingle-Ion Magnets: Solid-State Effects. *Inorg. Chem.* **2016**, *55* (19), 9537–9548. https://doi.org/10.1021/acs.inorgchem.6b00508.

(19) Ruamps, R.; Batchelor, L. J.; Guillot, R.; Zakhia, G.; Barra, A.-L.; Wernsdorfer, W.; Guihéry, N.; Mallah, T. Ising-Type Magnetic Anisotropy and Single Molecule Magnet Behaviour in Mononuclear Trigonal Bipyramidal Co(Ii) Complexes. *Chem. Sci.* **2014**, *5* (9), 3418–3424. https://doi.org/10.1039/C4SC00984C.

(20) Song, Y.; Zhang, P.; Ren, X. M.; Shen, X. F.; Li, Y. Z.; You, X. Z. Octacyanometallate-Based Single-Molecule Magnets: CoII9MV6 (M = W, Mo). *J. Am. Chem. Soc.* **2005**, *127* (11), 3708–3709. https://doi.org/10.1021/ja042334k.

(21) Ritchie, C.; Ferguson, A.; Nojiri, H.; Miras, H. N.; Song, Y.-F.; Long, D.-L.; Burkholder, E.; Murrie, M.; Kögerler, P.; Brechin, E. K.; Cronin, L. Polyoxometalate-Mediated Self-Assembly of Single-Molecule Magnets: $\{[XW_9O_{34}]_2[Mn^{III}_4Mn^{II}_2O_4(H_2O)_4]\}^{12-}$. *Angew. Chemie Int. Ed.* **2008**, *47* (30), 5609–5612. https://doi.org/10.1002/anie.200801281.

(22) Evans, D. F. The Determination of the Pararnagnetic Susceptibility. *J. Chem. Soc.* **1959**, *0*, 2003–2005.

(23) Sarkar, A.; Dey, S.; Rajaraman, G. Role of Coordination Number and Geometry in Controlling the Magnetic Anisotropy in FeII, CoII, and NiII Single-Ion Magnets. *Chem. – A Eur. J.* **2020**, *26* (62), 14036–14058. https://doi.org/https://doi.org/10.1002/chem.202003211.

(24) Jurca, T.; Farghal, A.; Lin, P. H.; Korobkov, I.; Murugesu, M.; Richeson, D. S. Single-Molecule Magnet Behavior with a Single Metal Center Enhanced through Peripheral Ligand Modifications. *J. Am. Chem. Soc.* **2011**, *133* (40), 15814–15817.

https://doi.org/10.1021/ja204562m.

(25) Habib, F.; Luca, O. R.; Vieru, V.; Shiddiq, M.; Korobkov, I.; Gorelsky, S. I.; Takase, M. K.; Chibotaru, L. F.; Hill, S.; Crabtree, R. H.; Murugesu, M. Influence of the Ligand Field on Slow Magnetization Relaxation versus Spin Crossover in Mononuclear Cobalt Complexes. *Angew. Chemie Int. Ed.* **2013**, *52* (43), 11290–11293. https://doi.org/10.1002/anie.201303005.

(26) Nemec, I.; Marx, R.; Herchel, R.; Neugebauer, P.; Van Slageren, J.; Trávníček, Z. Field-Induced Slow Relaxation of Magnetization in a Pentacoordinate Co(II) Compound [Co(Phen)(DMSO)Cl2]. *Dalt. Trans.* **2015**, *44* (33), 15014–15021. https://doi.org/10.1039/c5dt02162f.

(27) Schweinfurth, D.; Sommer, M. G.; Atanasov, M.; Demeshko, S.; Hohloch, S.; Meyer, F.; Neese, F.; Sarkar, B. The Ligand Field of the Azido Ligand: Insights into Bonding Parameters and Magnetic Anisotropy in a Co(Ii)-Azido Complex. *J. Am. Chem. Soc.* **2015**, *137* (5), 1993–2005. https://doi.org/10.1021/ja512232f.

(28) Shao, F.; Cahier, B.; Rivière, E.; Guillot, R.; Guihéry, N.; Campbell, V. E.; Mallah, T. Structural Dependence of the Ising-Type Magnetic Anisotropy and of the Relaxation Time in Mononuclear Trigonal Bipyramidal Co(II) Single Molecule Magnets. *Inorg. Chem.* **2017**, *56* (3), 1104–1111. https://doi.org/10.1021/acs.inorgchem.6b01966.

(29) Chen, L.; Song, J.; Zhao, W.; Yi, G.; Zhou, Z.; Yuan, A.; Song, Y.; Wang, Z.; Ouyang, Z. W., A Mononuclear Five-Coordinate Co(Ii) Single Molecule Magnet with a Spin Crossover between the S = 1/2 and 3/2 States. *Dalton Trans* **2018**, *47*, 16596-16602. https://doi.org/ 10.1039/c8dt03783c.

(30) M. J. Frisch, G. W. Trucks, H. B. Schlegel, G. E. Scuseria, M. A. Robb, J. R. Cheeseman, G. Scalmani, V. Barone, G. A. Petersson, H. Nakatsuji, X. Li, M. Caricato, A. V. Marenich, J. Bloino, B. G. Janesko, R. Gomperts, B. Mennucci, H. P. Hratchian, J. V., D. J. F. Gaussian 16, Revision C.01. *Gaussian, Inc., Wallingford CT* **2016**.

(31) Neese, F. The ORCA Program System. *WIREs Comput. Mol. Sci.* **2012**, *2* (1), 73–78. https://doi.org/10.1002/wcms.81.

(32) Cirera, J.; Ruiz, E.; Alvarez, S.; Neese, F.; Kortus, J. How to Build Molecules with Large Magnetic Anisotropy. *Chem. – A Eur. J.* **2009**, *15* (16), 4078–4087. https://doi.org/10.1002/chem.200801608.
